# Convergent and Efficient Total Synthesis of (+)-Heilonine Enabled by C–H Functionalizations

**DOI:** 10.1021/jacs.3c13492

**Published:** 2024-01-16

**Authors:** Yuan Jin, Sovanneary Hok, John Bacsa, Mingji Dai

**Affiliations:** † Department of Chemistry, Emory University, Atlanta, Georgia 30322, United States; ‡ Department of Pharmacology and Chemical Biology, Emory University, Atlanta, Georgia 30322, United States

## Abstract

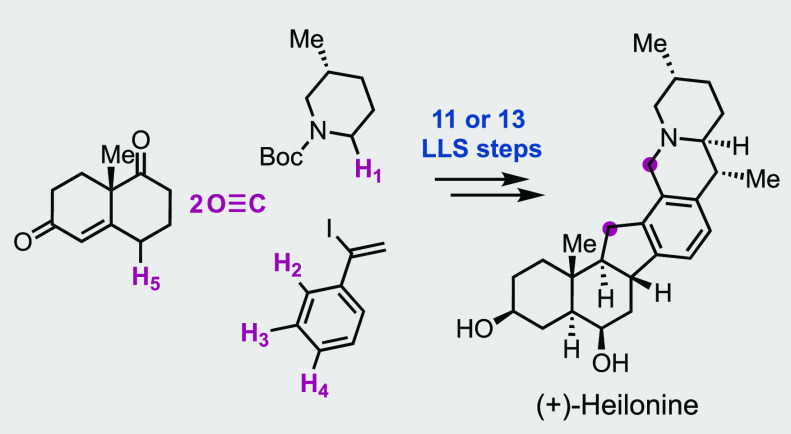

We report a convergent and efficient total synthesis of the C-*nor* D-*homo* steroidal alkaloid (+)-heilonine with a hexacyclic ring system, nine stereocenters, and a *trans*-hydrindane moiety. Our synthesis features four selective C–H functionalizations to form key C–C bonds and stereocenters, a Stille carbonylative cross-coupling to connect the AB ring system with the DEF ring system, and a Nazarov cyclization to construct the five-membered C ring. These enabling transformations significantly reduced functional group manipulations and delivered (+)-heilonine in 11 or 13 longest linear sequence (LLS) steps.

Heilonine (**1**, Figure 1) was isolated by Kaneko et al. in 1989 from *Fritillaria ussuriensis* Maxim. cultivated in the Hei-Long-Jiang province in China, from which its name was given.^1^*Fritillaria ussuriensis* Maxim. (also called Ping-bei-mu) is part of the Chinese herbal drug “Bei-mu”, which has been used as an effective antitussive, sedative, and expectorant. “Bei-mu” is a rich source of steroidal alkaloids with broad therapeutic potential.^2^ Heilonine belongs to the *Veratrum* steroidal alkaloid family whose members feature a common C-*nor* D-*homo* steroidal skeleton (Figure 1A).^3^ Based on the connectivity patterns around the piperidine E ring, the *Veratrum* steroidal alkaloids can be further divided into three subfamilies: cevanine (cf. **1**–**5**), jervine (cf. **6**, **7**), and veratramine (cf. **8**, **9**). Among them, cyclopamine (**7**) is arguably the most notable and investigated one.^4^ It was identified as a Hedgehog signaling pathway inhibitor, and its analogue patidegib is currently in human clinical trials for cancer treatment.

**Figure 1:**
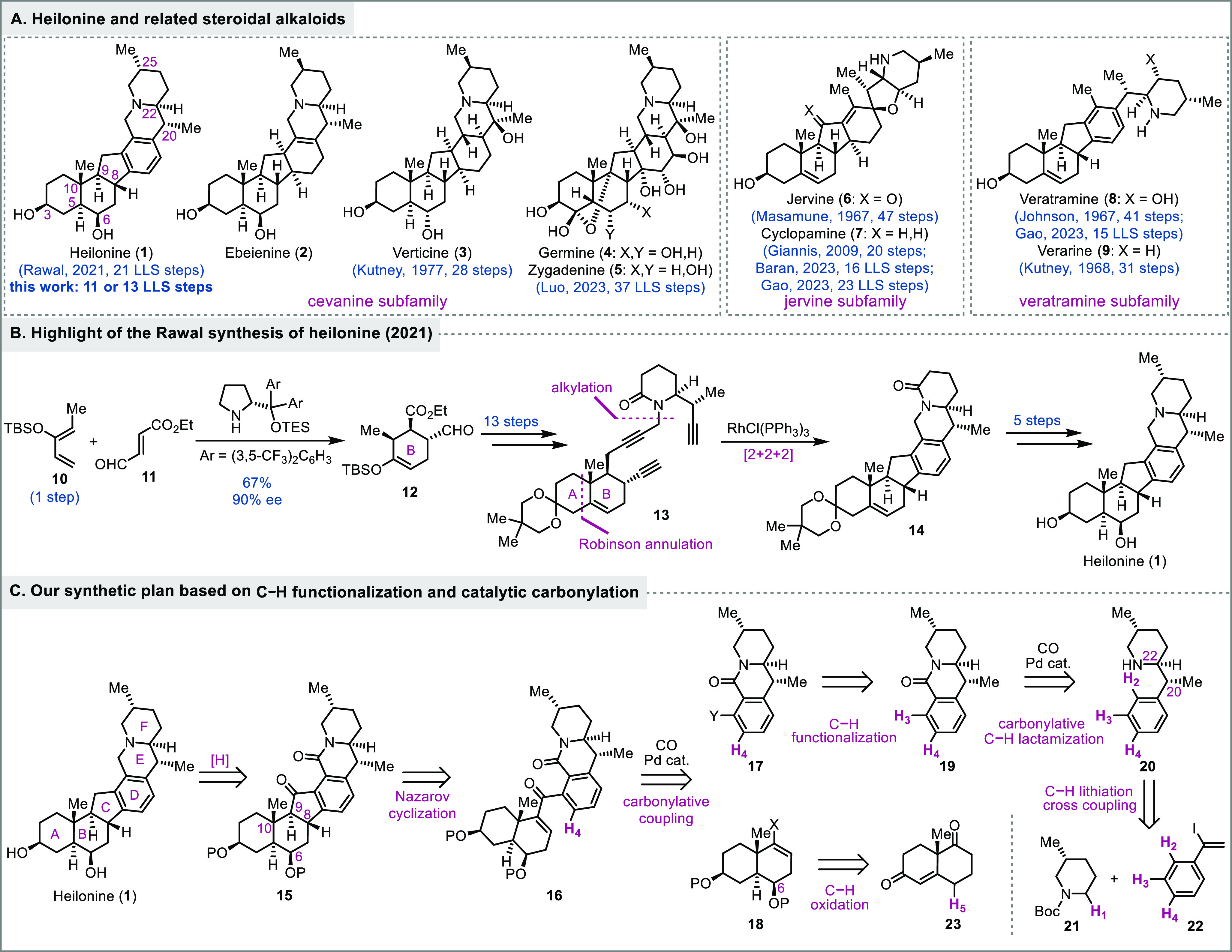
Heilonine and related steroidal alkaloids, prior synthesis, and our retrosynthetic analysis.

Due to the structural diversity, complexity, and therapeutic potential of the *Veratrum* steroidal alkaloids, they have attracted significant synthetic attention since the 1960s.^5,6^ In 1967, the Masamune group^5a^ and the Johnson group^5b^ reported breakthrough total syntheses of jervine (**6**) in 47 steps and veratramine (**8**) in 41 steps, respectively. Both syntheses could be traced back to Hagemann’s ester. Following these two total syntheses, Kutney et al. disclosed their syntheses of several *Veratrum* steroidal alkaloids including verarine (**9**) in 31 steps in 1968 and verticine (**3**) in 28 steps in 1977 from hecogenin acetate.^5c−5g^ While there was continued interest in the chemical synthesis of the *Veratrum* steroidal alkaloids,^6^ the next completed synthesis came about 30 years later when Giannis et al. disclosed a semisynthesis of cyclopamine from dehydroepiandrosterone in 20 steps in 2009.^5h^ In 2023, the Baran group^5i^ reported their convergent and enantioselective total synthesis of cyclopamine in 16 LLS steps followed by Gao and co-workers’ total synthesis in 23 LLS steps.^5j^ In the same year, Luo et al. achieved the first total synthesis of the highly oxygenated zygadenine (**5**) in 37 LLS steps.^5k^

Our continued interest in using carbonylation chemistry^7^ and C–H functionalization^8^ to streamline total synthesis of medicinally important natural products prompted us to embark on the total synthesis of heilonine with the goal to establish a general and efficient approach to access both natural and synthetic analogs for comprehensive biological evaluations. So far, there is only one total synthesis of heilonine, which was reported by Rawal and Cassaidy in 2021 in 21 LLS steps (30 steps total; Figure 1B).^9^ Their elegant synthesis features an enantioselective organocatalyzed Diels–Alder reaction to build the B ring with three chiral centers (**10** + **11** → **12**) and a Rh-catalyzed [2 + 2 + 2] cycloaddition to form the aromatic D-ring (**13** → **14**). Herein, we report a convergent total synthesis of (+)-heilonine from the Wieland–Miescher ketone and phenylacetylene in 11 or 13 LLS steps.

Heilonine is the first example of a cevanine alkaloid with an aromatic D-ring. It contains a hexacyclic ring system including a *trans-*hydrindane (BC ring). It has nine stereocenters, which reside in two different regions. Thus, independent stereochemical control of these two regions is required. It also has a basic tertiary amine and two secondary alcohols, one of which occupies an axial position. All of these structural features make heilonine a challenging target and offer an opportunity for chemistry innovation.

Retrosynthetically, we first added two carbonyl functionalities (one ketone and one lactam) to heilonine and proposed compound **15** as an advanced intermediate. The addition of the ketone group on the five-membered C ring would enable a Nazarov cyclization^10^ of **16** to close the five membered C ring and a highly convergent carbonylative cross-coupling strategy to bring together the AB (cf. **18**) and DEF (cf. **17**) ring systems and access **16** rapidly with carbon monoxide as a one-carbon linchpin. We were expecting that the angular methyl group at C10 and the protected axial secondary alcohol at C6 would force the aryl group to approach the enone from the bottom face, yielding the desired stereochemistry at C8. At the planning stage, it is difficult to predict the stereochemical outcome at C9, but it is epimerizable, which offers an opportunity to correct it if the undesired one is produced. Compound **18** could be traced back to the chiral pool molecule Wieland–Miescher ketone (**23**) with a selective C–H hydroxylation at C6. The lactam of **17** could serve both as a protected form of the tertiary amine and as a directing group for selective C–H functionalization at its *ortho* position to introduce handles (cf. **19** to **17**) for the carbonylative cross-coupling. We then proposed an amine-directed palladium-catalyzed carbonylative C–H lactamization to synthesize **19** from amine **20**.^11^ To quickly access **20** with three chiral centers, we proposed a Boc-directed and diastereoselective C–H lithiation of **21** followed by palladium-catalyzed Negishi cross-coupling of the in situ generated organozinc reagent with vinyl iodide **22** to forge the key C–C bond between C20 and C22.^12^ A subsequent diastereoselective *exo*-methylene reduction is needed to introduce the C20 stereocenter. Compound **21** can be synthesized via Boc protection of the corresponding amine,^13^ and compound **22** can be obtained via regioselective iodination of phenylacetylene.^14^

Our synthesis commenced from **21** and **22** (Scheme 1A). For the Negishi cross-coupling, the organozinc reagent was generated via Boc-directed lithiation of **21** with *s*-BuLi followed by transmetalation with ZnCl_2_. After modifying the conditions developed by Knochel and co-workers,^15^ the Negishi cross-coupling between the organozinc reagent derived from **21** and vinyl iodide **22** occurred with the Buchwald CPhos Pd G3 as catalyst.^16^ Compound **24** (CCDC 2306389) was produced in a highly regio- and diastereoselective manner with the desired stereochemistry at C22. The C25 stereocenter controlled the C22 stereocenter by enforcing a Boc-directed lithiation of the equatorial proton. After Boc removal with TFA, amine **25** was produced in 52% yield over two steps. To set up the C20 stereocenter, directed and catalytic hydrogenation with Crabtree’s catalyst was used to reduce the *exo*-methylene stereoselectively^17^ and delivered **20** in 88% yield as a single diastereomer (CCDC 2306390). The stereochemical outcome could be explained by a nitrogen-directed chelation model (cf. **25a**). For the next oxidative carbonylative C–H lactamization to close the E ring, we employed the conditions developed by Orito and co-workers.^11a^ With Pd(OAc)_2_ as catalyst and Cu(OAc)_2_ and air as co-oxidants, desired product **19** (CCDC 2306384) was produced in 82% yield under carbon monoxide atmosphere in refluxing toluene. The newly introduced lactam then served as a directing group to introduce a handle at its *ortho* position for the next carbonylative cross-coupling. At the exploration stage, we synthesized **17a** with a Bpin group and **17b** with an iodide. The former was prepared via an iridium-catalyzed C–H borylation developed by Chattopadhyay and co-workers,^18^ and the latter was accessed via a rhodium-catalyzed C–H iodination reported by Glorius et al.^19^ To test the feasibility of the carbonylative cross-coupling and the Nazarov cyclization, vinyltriflate **27** was prepared from Wieland–Miescher ketone in 40% yield.^20^ The latter was further converted to vinylstannane **28** in 83% yield using the Wulff method.^21^ We then investigated various palladium-catalyzed Suzuki (between **17a** and **27**) or Stille (between **17b** and **28**) carbonylative cross-coupling conditions but were unfortunately not successful. The information we gathered indicated that the lactam carbonyl group was problematic due to both the steric and electronic effects it generated. Thus, we decided to remove it before the carbonylative cross-coupling and were aware of potential difficulties in dealing with the resulting tertiary amine in the following steps. While reduction of **17a** was difficult, we were able to reduce **17b** with borane–THF to deliver **26** in 95% yield. Without the lactam carbonyl group, the Stille carbonylative cross-coupling between **26** and **28** occurred smoothly with cuprous chloride as an additive to facilitate the transmetalation step.^22^ Nazarov precursor **29** was obtained in 68% yield. We next examined the Nazarov cyclization under various conditions. Interestingly, under photochemical conditions (254 nm), instead of the Nazarov cyclization products, we obtained a mixture of spirocyclic compounds (dr 2.7:1) as the major identifiable products. The structure of the major diastereomer (**30**) was confirmed by X-ray crystallography (CCDC 2309636). The formation of **30** was proposed to go through intermediate **31** derived from the excitation of **29**. A subsequent 1,6-hydrogen atom abstraction would produce **32** with a benzylic radical further stabilized by the tertiary amine. Intramolecular radical recombination would give **30**. Since the tertiary amine complicated the Nazarov reaction, we decided to protect it in situ as an ammonium salt and treated **29** with triflic acid (TfOH) in hexafluoro-2-propanol (HFIP) at 60 °C.^10i,10k^ To our delight, the Nazarov cyclization took place under these conditions to give a mixture of **33** (43%; CCDC 2306386), **34** (41%), and **35** (11%; CCDC 2310768) in 95% total yield. Notably, HFIP helped to improve the overall yield and stereoselectivity at C8. Without HFIP, a 2.5/1 mixture of **33** (major, undesired) and **34** was obtained in 81% yield (see the Supporting Information). These results indicate that the angular methyl group exerts little stereochemical control at C8 (dr = 1.2/1 with HFIP). We suspected that a bulky group protected secondary alcohol at C6 could improve the diastereoselectivity. We further learned that *cis* hydrindane **34** can be partially epimerized to give the desired *trans* hydrindane **35**. These results encouraged us to prepare a more sophisticated vinylstanne (cf. **42**) for the carbonylative cross-coupling and Nazarov cyclization.

**Scheme 1:**
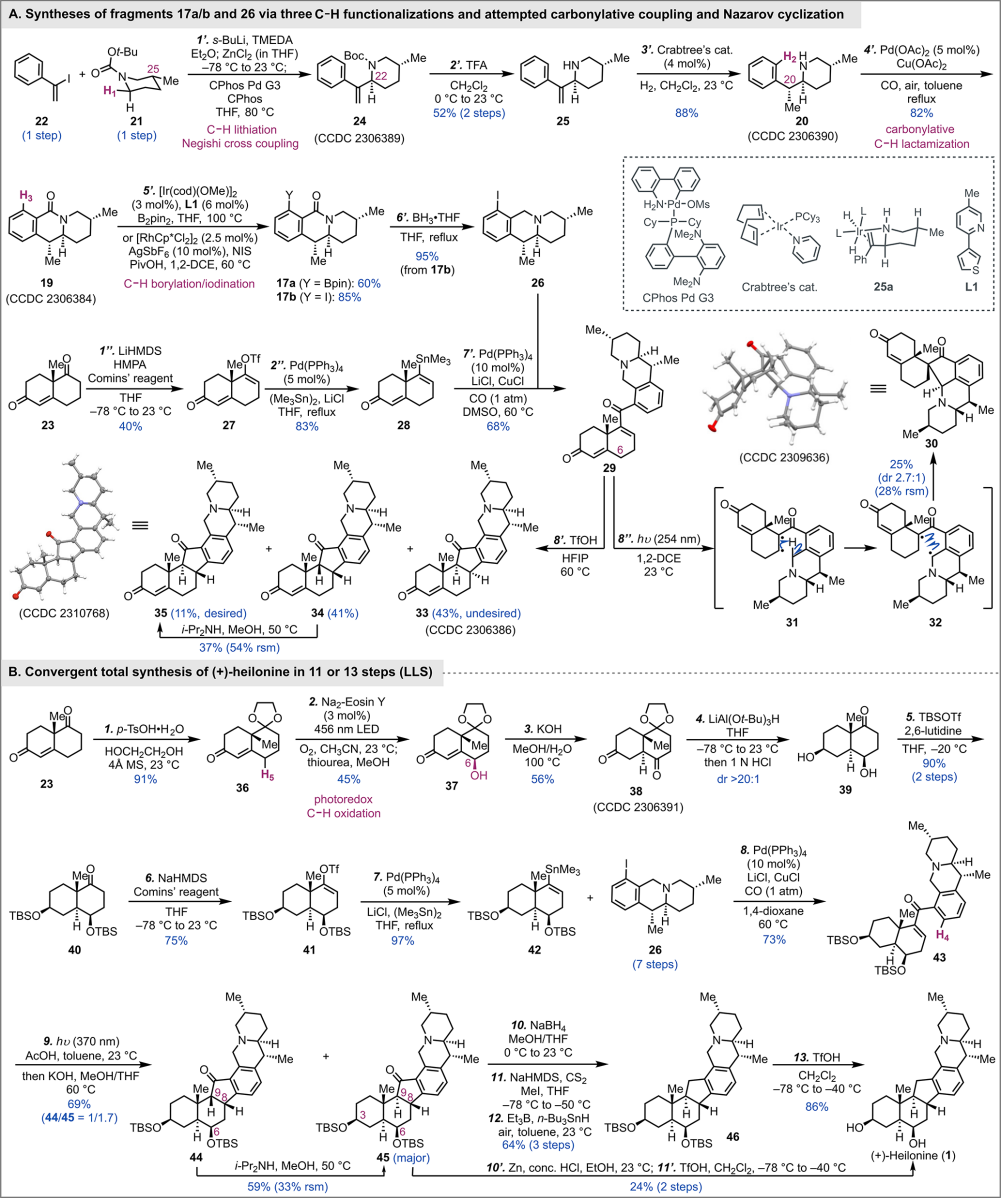
Total Synthesis of (+)-Heilonine

To synthesize **42**, Wieland–Miescher ketone **23** was converted to its monoketal **36** (Scheme 1B). While **36** could be advanced to **37** in two steps via extended silyl enol ether formation followed by Rubottom oxidation,^23^ we wondered whether a directed C–H hydroxylation at C6 could be realized and noted a Na_2_-Eosin Y-catalyzed visible-light-induced γ-hydroxylation of enones developed by Yue and Zheng recently.^24^ After finely tuning the catalyst loading, we were able to convert **36** to **37** in 45% yield. The main side reaction is overoxidation to a diketone. **37** was then isomerized to 1,4-dione **38** (CCDC 2306391) with a *trans* ring junction with KOH.^25^ Dual reduction of **38** using bulky LiAl(O*t*-Bu)_3_H^26^ followed by one-pot removal of the ketal protecting group delivered 1,4-diol **39** (dr >20:1), which was subsequently protected as bis-TBS ether **40** in 90% yield over two steps. Compound **40** was then advanced to vinylstannane **42** in two steps, namely, vinyl triflate formation and palladium-catalyzed stannylation. We then examined the Stille carbonylation between **42** and **26**. Unfortunately, the conditions we used for the coupling between **26** and **28** gave unsatisfactory results. After an extensive investigation, we learned that a simple switch of solvent from DMSO to 1,4-dioxane turned on the carbonylative cross-coupling between **42** and **26** to deliver **43** in 73% yield. We then encountered issues with the Nazarov cyclization. We explored various acidic and photochemical conditions and discovered that a combination of acetic acid and light at 370 nm was optimal. After in situ epimerization, a 1/1.7 mixture of **44** (*cis*)/**45** (*trans*) was obtained in 69% yield. The former could be further epimerized to the latter with *i*-Pr_2_NH in MeOH at 50 °C. Notably, with the C6 axial TBS ether, high stereochemical control at C8 was obtained. We next needed to reduce the ketone to a methylene. A one-step reduction turned out to be challenging. Among the conditions we explored, the Clemmensen reduction with Zn in HCl gave the methylene product with mono-TBS (C3) removal in 25% yield (see the Supporting Information). A subsequent C6 TBS deprotection with TfOH gave (+)-heilonine in 96% yield (11 LLS steps). On the other hand, we reduced the ketone to a secondary alcohol with NaBH_4_ and used the Barton–McCombie deoxygenation to deliver **46** in 64% yield over 3 steps.^27^ Final removal of the two TBS groups with TfOH gave (+)-heilonine in 86% yield (13 LLS steps).

In summary, this work highlights how transition metal catalysis and C–H functionalization chemistry^28^ can impact the efficiency of natural product synthesis. Four well-orchestrated C–H functionalizations, namely, Boc-directed C–H lithiation–Negishi cross-coupling, palladium-catalyzed carbonylative C–H lactamization, lactam directed rhodium-catalyzed C–H iodination, and Na_2_-Eosin Y-catalyzed visible-light-induced C–H hydroxylation enabled rapid syntheses of building blocks **26** and **42**, which were then linked together with a palladium-catalyzed Stille carbonylation for the subsequent photochemical Nazarov cyclization to build the hexacyclic skeleton. These enabling transformations allowed consecutive bond constructions around a monosubstituted aromatic starting material to produce the tetrasubstituted aromatic core of heilonine. Overall, total synthesis of (+)-heilonine was achieved in a highly convergent manner with 11 or 13 LLS steps.

## Supporting Information Available

Experimental procedures and NMR spectra for all new compounds (PDF)
