# Supplementary material for: Convergent and Efficient Total Synthesis of (+)-Heilonine Enabled by C–H Functionalizations
Source: J Am Chem Soc. 2024 Jan 16;146(3):1825–31. doi: 10.1021/jacs.3c13492 (PMC10811669; doi:10.1021/jacs.3c13492)

Supporting Information

Convergent and Efficient Total Synthesis of (+)-Heilonine Enabled by C–H Functionalizations

Yuan Jin,[1] Sovanneary Hok,[1] John Bacsa[1] and Mingji Dai*,[1,2]

[1]Department of Chemistry, Emory University, Atlanta, GA 30322, United States

[2]Department of Pharmacology and Chemical Biology, Emory University, Atlanta, GA 30322, United States

Table of Contents

Part 1. Experimental procedures and spectra data..S3

Part 2. X-ray structure and analysis data...S37

Part 3. References..S51

Part 4. ^{1}H and ^{13}C NMR spectra...S52

Part 1. Experimental procedures and spectra data.

General Methods: Reactions were performed using standard syringe techniques under argon unless stated otherwise. Starting materials and reagents were used as received from suppliers. Dichloromethane (CH_2Cl_2), tetrahydrofuran (THF), and toluene were purified by passing the previously degassed solvents through activated alumina columns. Flash chromatography was performed using silica gel (230-400 mesh). Thin layer chromatography (TLC) was performed using glass-backed silica plates (Silicycle). NMR spectra were recorded on a Bruker AVIII 400 or Bruker NEO 400 or Bruker AVIII HD 600 or Bruker NEO 800 M Hz spectrometer at room temperature. Chemical shifts (in ppm) are given in reference to the solvent signal [^{1}H NMR: $CDCl_3$ (7.26); ^{13}C NMR: $CDCl_3$ (77.00)]. ^{1}H NMR data are reported as follows: chemical shifts (δ ppm), multiplicity (s = singlet, d = doublet, t = triplet, q = quartet, quin = quintuplet, m = multiplet, br = broad), coupling constant (Hz), and integration. ^{13}C NMR data are reported in terms of chemical shift and multiplicity. IR data were recorded on a Thermofisher Scientific NICOLET iS10 instrument. Optical resolution was measured on a LAXCO SGW-5 automatic polarimeter or RUDOLPH APIV-1W automatic polarimeter. Melting point was measured on a MEL-TEMP 1001D. Room temperature is around 23 °C.

Experimental procedure

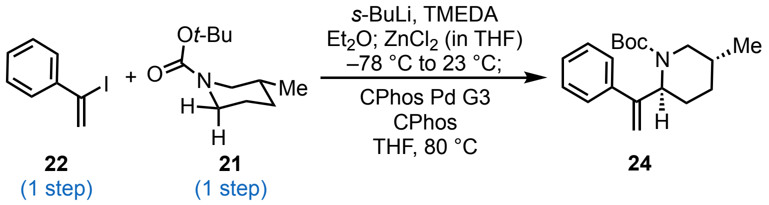

22 (1 step) **21** (1 step) **24**

Note: Compounds **22**[1] and (–)-**21**[2] (**[α]**$_D^{21}$ = –29.8° (c =1.000 g / 100 mL in $CHCl_3$)) were synthesized according to the reported procedures. For the synthesis of **24**, the procedure reported by Knochel et al.[3] was followed with a few alterations.

A 100 mL round bottom flask equipped with a stir bar was charged with **21** (2.18 g, 10.96 mmol, 1.9 equiv.). The material was dried by azeotropic distillation with toluene for three times and further dried under vacuum. The flask was then backfilled with argon. To the material, anhydrous Et_2O (22 mL) and TMEDA (1.64 mL, 10.96 mmol, 1.9 equiv.) were added. The solution was cooled to –78 °C and *s*-BuLi (1.4 M in cyclohexane) (9.4 mL, 13.15 mmol, 2.3 equiv.) was slowly added over 70 min. The reaction mixture was further stirred for 4 h at –78 °C. To the reaction mixture, freshly prepared $ZnCl_2$ (1.0 M in THF) (13.2 mL, 13.2 mmol, 2.3 equiv.) was added dropwise over 35 min. The reaction mixture was further stirred for 15 min at –78 °C and then allowed to warm to room temperature. After stirring at room temperature for 40 min, volatiles were removed under vacuum (100 min, 0.7 torr) with stirring and backfilled with argon, then THF (12 ml) was added to the resulting white gel to get a clear organozinc solution. Meanwhile, in a glove box, to a suspension of CPhos Pd G3 (210.5 mg, 0.26 mmol, 0.05 equiv.) and CPhos (114 mg, 0.26 mmol, 0.05 equiv.) in THF (1 mL) in an oven dried microwave vial was added alkenyl iodide **22** (1.30 g, 5.65 mmol, 1.0 equiv., dried by azeotropic distillation with toluene) in THF (4 mL) and the vial was sealed. The microwave vial was taken out from glove box and charged with an argon balloon. The prepared organozinc solution was added to the microwave vial by a syringe at room temperature and a clear brown solution was obtained. The argon balloon was removed. The resulting solution was heated to 80 °C for 1.5 h and the clear brown solution became a yellow suspension. It was then cooled to room temperature and the yellow suspension became clear brown solution again. It was then heated to 80 °C for 16 h and at the end of the reaction, eventually a black solution was obtained.

(Notice: when running a big scale reaction, after heating the solution to 80 °C, cool back to room temperature to get a clear brown solution again is critical. Based on TLC

checking, heating at 80 °C without cooling and re-heating process will sometimes lead to a yellow suspension and alkenyl iodide would not be consumed sufficiently)
After the reaction mixture was cooled down to room temperature, saturated NH_4Cl solution was added to the resulting black reaction mixture. The phases were separated, and the aqueous phase was extracted with Et_2O (4 x 20 mL). The combined organic layers were washed with brine (40 mL) and dried over anhydrous Na_2SO_4. The solvents were evaporated and the residue was purified by column chromatography (hexane:EtOAc = 50:1) to afford alkene **24** and recovered **21** as an inseparable mixture (1.25 g, **24**/**21**=2/1, determined by ^{1}H NMR). During the column chromatography, the first several fractions containing **24** were pure and the data were collected using the pure sample.

M.P.: 58-61 ˚C

$\boldsymbol{R_f}$ = 0.5 (hexane:EtOAc = 10:1; UV)

$[\alpha]_D^{21}$= +20.5° (c = 1.000 g / 100 mL in $CHCl_3$)

IR (thin film) 2961, 2929, 2870, 1688, 1413, 1207, 1059 cm^{-1}

^{1}H NMR (400 MHz, $CDCl_3$) δ 7.36 – 7.22 (m, 5H), 5.26 (s, 1H), 5.22 (br s, 1H), 5.09 (s, 1H), 3.62 (d, J = 13.4 Hz, 1H), 3.08 (dd, J = 13.4, 3.9 Hz, 1H), 1.96 – 1.82 (m, 2H), 1.75 (m, 1H), 1.64 – 1.53 (m, 1H), 1.44 (s, 9H), 1.24 – 1.13 (m, 1H), 1.00 (d, J = 6.9 Hz, 3H) ppm

^{13}C NMR (100 MHz, $CDCl_3$) δ 155.9, 148.5, 141.2, 128.1, 127.3, 127.0, 113.6, 79.3, 53.7, 45.1, 28.4, 27.9, 25.7, 21.9, 17.3 ppm

HRMS (ESI) m/z $[M+Na]^+$ calcd for $C_{19}H_{27}O_2NNa$: 324.1934; found: 324.1942

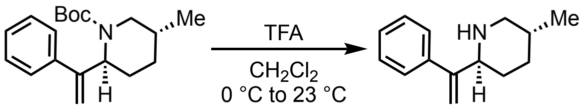

24 **25**

To the above mixture of **24** and **21** (1.25 g) in dichloromethane (39 mL) was slowly added trifluoroacetic acid (13 mL) at 0 ˚C. After stirring at 0 ˚C for 5 min, the solution was allowed to warm to room temperature. After stirring for 16 h at room temperature, the resulting solution was cooled to 0 ˚C and 3.75 M NaOH solution was slowly added until the pH reached 14. The mixture was extracted 3 times with dichloromethane. The combined organic layers were washed with brine and dried over anhydrous Na_2SO_4. The solvent was evaporated and the residue was purified by column chromatography (3% Et_3N in hexane:EtOAc = 15:1) to afford piperidine **25** (586 mg, 52% for 2 steps, based on **22**) as a pale yellow liquid.

$\boldsymbol{R_f}$ = 0.3 (3% Et_3N in hexane:EtOAc = 15:2; UV)

[α]$_D$^{21}= –6.4° (c = 1.000 g / 100 mL in $CHCl_3$)

IR (thin film) 3266, 2923, 1626, 1457, 1375, 1117, 903 cm^{-1}

^{1}H NMR (400 MHz, $CDCl_3$) δ 7.43 – 7.23 (m, 5H), 5.26 (s, 1H), 5.21 (s, 1H), 3.47 (d, J = 11.0 Hz, 1H), 3.12 (ddd, J = 11.7, 4.0, 2.0 Hz, 1H), 2.37 (dd, J = 11.3, 11.3 Hz, 1H), 1.87-1.71 (m, 2H), 1.66-1.50 (m, 1H), 1.37-1.23 (m, 1H), 1.15-1.01 (m, 1H), 0.86 (d, J = 6.6 Hz, 3H) ppm

^{13}C NMR (100 MHz, $CDCl_3$) δ 152.2, 141.3, 128.3, 127.4, 126.8, 111.5, 59.6, 55.3, 34.1, 32.2, 31.8, 19.5 ppm

HRMS (ESI) *m*/*z* [M+H]$^+$ calcd for $C_{14}H_{20}N$: 202.1590; found: 202.1591

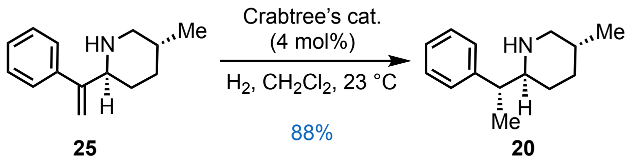

25 **20**

In a glove box, to **25** (586 mg, 2.91 mmol, 1.0 equiv.) in a vial was added Crabtree's catalyst (82 mg, 0.10 mmol, 0.03 equiv.) and dichloromethane (18 mL). Then the vial was taken out from glove box and charged with a H_2 balloon. The mixture was bubbled with H_2 for 20 min and then stirred at room temperature for 22 h with a H_2 balloon (1 atm). The H_2 balloon was replaced with an argon balloon. Dichloromethane (3 mL) was added, and the mixture was bubbled by argon for 10 min. To the resulting solution, Crabtree's catalyst (19 mg, 0.02 mmol, 0.007 equiv.) was added and the argon balloon was replaced with a H_2 balloon. The mixture was bubbled by H_2 for 20 min and then stirred at room temperature for 18 h with a H_2 balloon (1 atm). The solvent was evaporated and the residue was purified by column chromatography (3% Et_3N in hexane:EtOAc = 15:1) to afford piperidine **20** (520 mg, 88%) as a pale yellow liquid.

R$_f$ = 0.3 (3% Et_3N in hexane:EtOAc = 15:2; visualized with CAM stain)

[α]$_D$^{21}= –13.9° (c = 1.000 g / 100mL in $CHCl_3$)

IR (thin film) 3337, 2913, 2783, 1452, 1282, 761, 700, cm^{-1}

^{1}H NMR (400 MHz, $CDCl_3$) δ 7.34 – 7.27 (m, 2H), 7.25 – 7.18 (m, 3H), 2.86 (ddd, J = 11.3, 4.0, 2.1 Hz, 1H), 2.56 (qd, J = 9.0, 6.9 Hz, 1H), 2.46 (ddd, J = 10.8, 8.8, 2.4 Hz, 1H), 2.08 (dd, J = 11.0, 11.0 Hz, 1H), 1.93 (dddd, J = 12.7, 3.1, 3.1, 3.1 Hz, 1H), 1.87 – 1.77 (m, 1H), 1.61 – 1.45 (m, 1H), 1.29 – 1.13 (m, 4H), 0.98 (dddd, J = 13.0, 13.0, 13.0, 3.8 Hz, 1H), 0.80 (d, J = 6.6 Hz, 3H) ppm

^{13}C NMR (100 MHz, $CDCl_3$) δ 145.4, 128.6, 127.7, 126.4, 62.2, 55.1, 46.1, 33.9, 31.6, 30.4, 19.5, 18.6 ppm

HRMS (ESI) *m*/*z* [M+H]$^+$ calcd for $C_{14}H_{22}N$: 204.1747; found: 204.1749

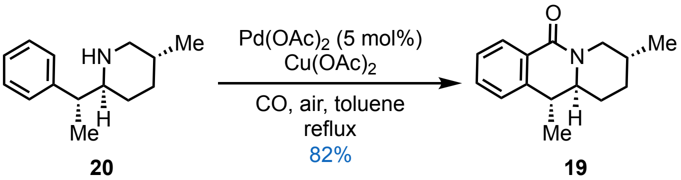

To a solution of **20** (520 mg, 2.56 mmol, 1.0 equiv.) in toluene (520 mL) was added $Pd(OAc)_2$ (28.7 mg, 0.128 mmol, 0.05 equiv.) and $Cu(OAc)_2$ (232.5 mg, 1.28 mmol, 0.5 equiv.). The mixture was bubbled with CO gas for 25 min. A CO gas flushed condenser was then attached. A CO balloon (ca. 2 L) was charged and air (160 mL, corresponding to 1.33 mmol of O_2, 0.5 equiv.) was injected into the reaction system through the septum stopper by a syringe. The resulting mixture was heated to reflux (120 ˚C oil bath) for 24 h. The reaction mixture was cooled to room temperature and filtered through a pad of powdered $MgSO_4$. The solvent was evaporated and the residue was purified by column chromatography (hexane:EtOAc = 10:1) to afford lactam **19** (480 mg, 82%) as a white solid.

M.P.: 94-96 ˚C

$\boldsymbol{R_f}$ = 0.3 (hexane:EtOAc = 5:1; UV)

$[\alpha]_D^{21}$= –229.6° (c = 1.000 g / 100 mL in $CHCl_3$)

IR (thin film) 2953, 2924, 1644, 1604, 1466, 1277, 761 cm^{-1}

^{1}H NMR (400 MHz, $CDCl_3$) δ 8.13 (dd, *J* = 7.8, 1.5 Hz, 1H), 7.43 (ddd, *J* = 7.5, 7.5, 1.5 Hz, 1H), 7.32 (ddd, *J* = 7.6, 7.6, 1.3 Hz, 1H), 7.16 (dd, 8.2, 1.4 Hz, 1H), 4.74 (ddd, *J* = 12.7, 4.4, 2.1 Hz, 1H), 3.30 (ddd, *J* = 12.0, 2.4, 2.4 Hz, 1H), 2.83 (qd, *J* = 7.1, 2.3 Hz, 1H), 2.30 (dd, *J* = 12.7, 11.4 Hz, 1H), 1.92 – 1.81 (m, 1H), 1.75 – 1.61 (m, 2H), 1.46 (dddd, *J* = 12.8, 12.8, 12.8, 3.8 Hz, 1H), 1.34 (d, *J* = 7.2 Hz, 3H), 1.25 (dddd, *J* = 13.0, 13.0, 13.0, 4.1 Hz, 1H), 0.92 (d, *J* = 6.6 Hz, 3H) ppm

^{13}C NMR (100 MHz, $CDCl_3$) δ 163.1, 142.0, 131.9, 128.5, 127.1, 127.0, 126.8, 63.5, 52.5, 37.2, 33.9, 32.3, 31.2, 23.0, 19.1 ppm

HRMS (ESI) *m*/*z* $[M+H]^+$ calcd for $C_{15}H_{20}ON$: 230.1539; found: 230.1540

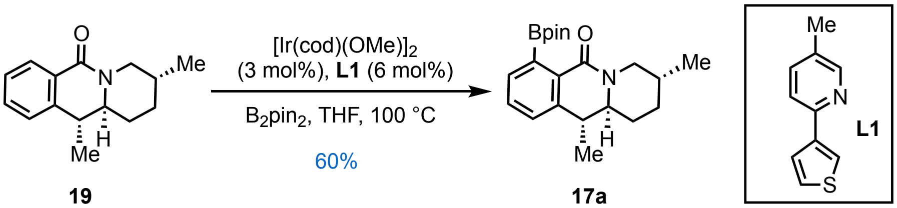

In a glove box, an oven dried microwave vial was charged with $[Ir(cod)(OMe)]_2$ (2.3 mg, 6.5 μmol, 0.03 equiv.), ligand **L1** (2.3 mg, 13 μmol, 0.06 equiv.), B_2pin_2 (58.1 mg, 0.229

mmol, 1.0 equiv.) and anhydrous THF (1.1 mL). The reaction mixture was stirred for 2 minutes at room temperature. To this mixture, lactam **19** (50 mg, 0.22 mmol, 1.0 equiv.) was added. The microwave vial was sealed and the reaction mixture was stirred for 12 h at 100 ˚C. After cooling to room temperature, the solvent was evaporated and the residue was purified by column chromatography (hexane:EtOAc = 5:1) to give a mixture (55 mg, **17a**:**19** = 3.7:1, based on ^{1}H NMR spectrum) of borylated product **17a** (60%, calculated yield) and lactam **19** (16%, calculated yield).
Due to the close R_f values, separation of **17a** and **19** by column chromatography was very difficult. The spectroscopic data of borylated product **17a** were collected after re-purification by preparative TLC (hexane:EtOAc = 5:1).
R_f = 0.32 (hexane:EtOAc = 5:1; UV)
$[\alpha]_D^{21}$= –79.2° (c = 0.200 g / 100 mL in $CHCl_3$)
IR (thin film) 2971, 2926, 1641, 1592, 1483, 1370, 1151 cm^{-1}
^{1}H NMR (600 MHz, $CDCl_3$) δ 7.41 (d, J = 7.4, 7.4 Hz, 1H), 7.36 (d, J = 7.2 Hz, 1H), 7.11 (d, J = 7.3 Hz, 1H), 4.62 (ddd, J = 12.9, 4.4, 2.1 Hz, 1H), 3.24 (ddd, J = 12.0, 3.2, 3.2 Hz, 1H), 2.84 (qd, J = 7.0, 3.9 Hz, 1H), 2.33 (dd, J = 11.6 Hz,1H), 1.89 – 1.82 (m, 1H), 1.81 – 1.67 (m, 2H), 1.53 – 1.37 (m, 13H), 1.31 (d, J = 7.1 Hz, 3H), 1.28 – 1.17 (m, 1H), 0.92 (d, J = 6.6 Hz, 3H) ppm
^{13}C NMR (150 MHz, $CDCl_3$) δ 165.5, 139.4, 132.1, 129.9, 129.5, 126.2, 82.6, 63.3, 52.0, 37.0, 33.5, 32.6, 30.9, 25.0, 25.0, 21.9, 19.1 ppm
^{11}B NMR (128 MHz, $CDCl_3$) δ 27.65 ppm
HRMS (ESI) *m/z* $[M+H]^+$ calcd for $C_{21}H_{31}O_3NB$: 356.2392; found: 356.2391

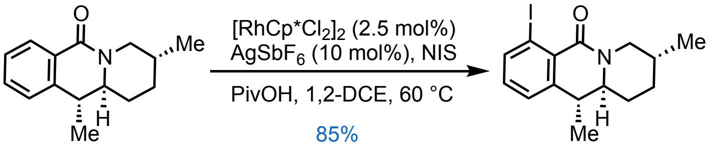

19 **17b**

In a glove box, $[RhCp^*Cl_2]_2$ (32.3 mg, 0.052 mmol, 0.025 equiv.), $AgSbF_6$ (71.9 mg, 0.209 mmol, 0.1 equiv.), PivOH (235 mg, 2.30 mmol, 1.1 equiv.) and NIS (518 mg, 2.30 mmol, 1.1 equiv.) were added to a microwave vial containing lactam **19** (480 mg, 2.09 mmol, 1.0 equiv., dried by azeotropic distillation with toluene). The microwave vial was sealed and taken out from the glove box. The vial was charged with an argon balloon and 1,2-DCE (10.5 mL). The argon balloon was removed, and the sealed vial was stirred at 60 ˚C for 17 h. The mixture was then allowed to cool down to room temperature, diluted with EtOAc, filtered through a short pad of silica gel, and eluted with EtOAc. After removing the solvent under reduced pressure, the product was purified by column chromatography

(hexane:EtOAc = 10:1) to afford iodine **17b** (0.7 g, contains impurities) as a yellow solid. The solid was dissolved in EtOAc and washed 3 times with saturated $NaHCO_3$ solution. The combined aqueous layer was extracted 3 times with EtOAc and the whole organic extracts were dried over anhydrous Na_2SO_4. The solvent was evaporated to afford pure iodide **17b** (629 mg, 85%) as a yellow solid.

R_f = 0.33 (hexane:EtOAc = 5:1; UV)

$[\alpha]_D^{21}$= –176.5° (c = 1.000 g / 100 mL in $CHCl_3$)

IR (thin film) 2924, 2868, 1647, 1458, 1369, 1260, 947 cm^{-1}

^{1}H NMR (400 MHz, $CDCl_3$) δ 8.03 (dd, *J* = 7.9, 1.3 Hz, 1H), 7.16 (dd, *J* = 7.5, 0.7 Hz, 1H), 7.00 (dd, *J* = 7.7, 7.7 Hz, 1H), 4.76 (ddd, *J* = 12.7, 4.4, 2.0 Hz, 1H), 3.27 (ddd, *J* = 12.0, 2.3, 2.3 Hz, 1H), 2.81 (qd, *J* = 7.2, 2.0 Hz, 1H), 2.30 (dd, *J* = 12.8, 11.4 Hz, 1H), 1.93-1.82 (m, 1H), 1.75 – 1.57 (m, 2H), 1.43 (dddd, *J* = 12.6, 12.6, 12.6, 3.9 Hz, 1H), 1.33 (d, *J* = 7.2 Hz, 3H), 1.25 (dddd, *J* = 12.9, 12.9, 12.9, 4.1 Hz, 1H), 0.90 (d, *J* = 6.5 Hz, 3H) ppm

^{13}C NMR (100 MHz, $CDCl_3$) δ 160.3, 144.4, 142.1, 132.2, 128.2, 126.3, 94.8, 62.3, 53.1, 37.8, 33.8, 31.9, 31.4, 23.1, 19.0 ppm

HRMS (ESI) *m/z* $[M+H]^+$ calcd for $C_{15}H_{19}ONI$: 356.0506; found: 356.0507.

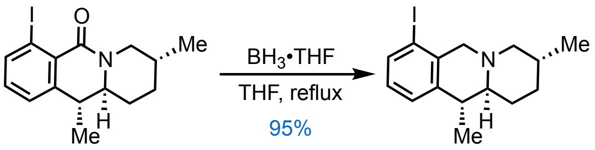

17b **26**

To a sealed microwave vial containing a solution of lactam **17b** (300 mg, 0.84 mmol, 1.0 equiv.) in THF (8 mL) was added dropwise BH_3•THF (1 M in THF, 12 mL, 12 mmol, 14.3 equiv.) under argon atmosphere at room temperature. The sealed vial was stirred at 76 °C for 23 h. After the reaction was cooled down to room temperature, the reaction mixture was poured into a cooled saturated $NaHCO_3$ solution (50 mL) and 4.4 M NaOH solution was added until the pH reached 14. The resulting mixture was extracted 3 times with Et_2O, the combined organic layers were washed with brine and dried over anhydrous Na_2SO_4. The solvent was evaporated, and the resulting residue was dissolved in a mixture of THF (14 mL) and 1 N HCl solution (14 mL), and the resulting mixture was stirred at 60 ˚C for 3 h. The reaction mixture was cooled to 0 ˚C and Et_2O was added. To the mixture, 4.4 M NaOH solution was added until the pH reached 14. The resulting mixture was extracted 3 times with Et_2O, then the combined organic layer was washed with brine and dried over anhydrous Na_2SO_4. The solvent was evaporated and the resulting residue was purified by

column chromatography (3% Et_3N in hexane:Et_2O = 40:1) to afford iodide **26** (273 mg, 95%) as a white solid.

***R**$_f$* = 0.3 (3% Et_3N in hexane:Et_2O = 40:1; UV)

$[\alpha]_D^{21}$= +52.7° (c = 1.000 g / 100 mL in $CHCl_3$)

IR (thin film) 2946, 2914, 2868, 2741, 1561, 1366, 1106 cm^{-1}

^{1}H NMR (400 MHz, $CDCl_3$) δ 7.61 (d, *J* = 7.8 Hz, 1H), 7.21 (d, *J* = 7.8 Hz, 1H), 6.87 (t, *J* = 7.8 Hz, 1H), 3.84 (d, *J* = 15.5 Hz, 1H), 3.14 – 3.04 (m, 2H), 2.73 (qd, *J* = 7.3, 7.3 Hz, 1H), 2.12 (dddd, *J* = 13.2, 3.2, 3.2, 3.2 Hz, 1H), 1.88 – 1.69 (m, 4H), 1.37 – 1.21 (m, 4H), 1.07 – 0.84 (m, 4H) ppm

^{13}C NMR (100 MHz, $CDCl_3$) δ 141.7, 136.3, 136.1, 128.1, 127.4, 98.3, 65.4, 63.9, 63.9, 39.7, 32.9, 31.9, 30.9, 19.6 (2 carbons overlapped) ppm

HRMS (ESI) *m/z* $[M+H]^+$ calcd for $C_{15}H_{21}NI$: 342.0713; found: 342.0711

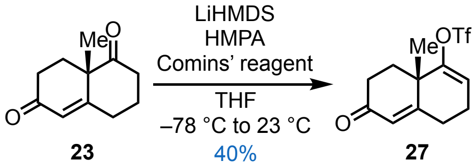

Note: The (*S*)-(+)-Wieland-Miescher ketone and Comins' reagent were dried 3 times by azeotropic removal of water by evaporation of a solution in toluene under reduced pressure before using.

To a solution of (*S*)-(+)-Wieland-Miescher ketone **23** (200 mg, 1.12 mmol, 1.0 equiv.) and anhydrous HMPA (0.98 mL, 5.6 mmol, 5.0 equiv.) in anhydrous THF (7.5 mL) was added dropwise LiHMDS (1 M in THF, 1.3 mL, 1.3 mmol, 1.2 equiv.) at –78 °C. After stirring 5 min at –78 °C, the mixture was allowed to warm up to room temperature over 3 h, then stirred at room temperature for 30 min. To the mixture, Comins' reagent (660 mg, 1.68 mmol, 1.5 equiv.) in anhydrous THF (3.0 mL) was added dropwise at room temperature. After stirring for 1.5 h at room temperature, the reaction mixture was poured to a mixture of EtOAc (50 ml) and water (50 ml). The organic phase was separated, and the aqueous phase was further extracted successively with EtOAc (1x50 mL) and Et_2O (2x50 mL). The whole organic extracts were then washed with a 1 N NaOH solution (2x40 mL), and successively by a pH 3 buffer solution (3x30 mL), water (1x40 mL), a pH 7 buffer solution (40 mL), and brine (50 mL). During the extraction, if the layers are not clearly separated, adding a small amount of brine will help. After drying over Na_2SO_4 and evaporation of the solvents under reduced pressure, the residue was purified by flash column chromatography (hexane:EtOAc = 10:1) to afford vinyl triflate **27** (140 mg, 40%) as a yellow solid. The data fully match the reported data.[4]

***R*$_f$** = 0.4 (hexane:EtOAc = 3:1; UV)

[α]$_D$^{21}= +211.5° (c = 1.050 g / 100 mL in $CHCl_3$)

IR (thin film) 2952, 1676, 1630, 1412, 1208, 1139, 881 cm^{-1}

^{1}H NMR (400 MHz, $CDCl_3$) δ 5.95 – 5.79 (m, 2H), 2.63 – 2.37 (m, 5H), 2.30 (dddd, *J* = 17.7, 12.3, 5.6, 1.9 Hz, 1H), 2.21 – 2.13 (m, 1H), 2.09 – 1.97 (ddd, *J* = 11.7, 11.7, 7.8 Hz, 1H), 1.47 (s, 3H) ppm

^{13}C NMR (100 MHz, $CDCl_3$) δ 197.6, 163.9, 152.2, 125.5, 118.3 (q, *J* = 317.4 Hz), 115.9, 39.9, 33.3, 31.9, 28.9, 23.8, 22.0 ppm

HRMS (ESI) *m*/*z* $[M+H]^+$ calcd for $C_{12}H_{14}O_4F_3S$: 311.0559; found: 311.0556

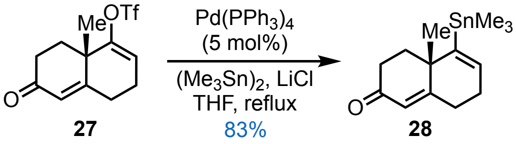

In a glove box, to a microwave vial containing vinyl triflate **27** (140 mg, 0.451 mmol, 1.0 equiv.) was added anhydrous LiCl (96 mg, 2.3 mmol, 5.1 equiv.), $Pd(PPh_3)_4$ (26.1 mg, 0.023 mmol, 0.05 equiv.), THF (9 mL) and hexamethylditin (0.38 mL, 1.8 mmol, 4.0 equiv.). The vial was sealed and taken out from the glove box. After stirring for 14 h at 80 °C, the reaction was cooled to room temperature, diluted with Et_2O, filtered through a short pad of celite, and eluted with Et_2O. The filtrate was washed twice with brine. The organic phase was dried over anhydrous Na_2SO_4, filtered and concentrated under reduced pressure. The residue was purified by flash column chromatography (hexane:EtOAc = 10:1) to afford vinyl stannane **28** (122 mg, 83%) as pale yellow oil.

***R*$_f$** = 0.2 (hexane:EtOAc = 10:1; UV)

[α]$_D$^{21}= +149.1° (c = 1.930 g / 100 mL in $CHCl_3$)

IR (thin film) 2966, 2915, 1670, 1621, 1577, 1430, 1348, 767 cm^{-1}

^{1}H NMR (400 MHz, $CDCl_3$) δ 5.92 – 5.65 (m, 2H), 2.66 – 2.48 (m, 2H), 2.48 – 2.23 (m, 4H), 1.94-1.80 (m, 2H), 1.33 (s, 3H), 0.18 (s, 9H) ppm

^{13}C NMR (100 MHz, $CDCl_3$) δ 199.2, 171.3, 151.0, 134.8, 123.5, 41.8, 37.2, 34.5, 30.3, 30.2, 25.4, -7.3 ppm

HRMS (ESI) *m*/*z* $[M+H]^+$ calcd for $C_{14}H_{23}OSn$: 327.0765; found: 327.0762

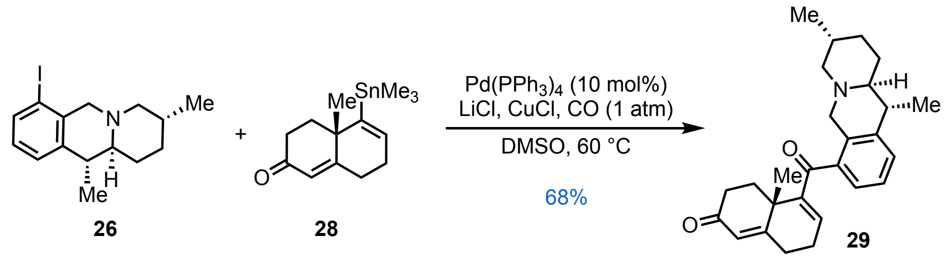

In a glove box, an oven dried microwave vial was charged with anhydrous LiCl (69 mg, 1.6 mmol, 5.9 equiv.), $Pd(PPh_3)_4$ (31.2 mg, 0.027 mmol, 0.1 equiv.) and CuCl (134 mg, 1.35 mmol, 5.0 equiv.). Subsequently, a solution of iodide **26** (91 mg, 0.27 mmol, 1.0 equiv.) in freshly degassed DMSO (2.7 mL) and a solution of vinyl stannane **28** (90 mg, 0.28 mmol, 1.0 equiv.) in freshly degassed DMSO (2.8 mL) were added. The vial was sealed and taken out from the glove box. The reaction vial was charged with a CO balloon and the reaction mixture was bubbled with CO for 30 min. The reaction mixture was stirred at 60 °C for 24 h with a CO balloon (1 atm). The reaction mixture was cooled to room temperature, diluted with Et_2O and a mixture of brine and 5% aqueous NH_4OH (5/1, v/v) was added. The organic phase was separated, and the aqueous phase was then further extracted twice with Et_2O. The combined organic layers were washed twice with water, twice with brine, dried over anhydrous Na_2SO_4, and concentrated to a residue. The residue was first purified by flash column chromatography (hexane:EtOAc= gradually from 3:1 to 1:2) to afford enone **29** which contains impurities. The material was re-purified by flash column chromatography (3% Et_3N in hexane:EtOAc = 3:1) to afford pure enone **29** (73 mg, 68%, based on **26**) as a white foam.

R_f = 0.1 (hexane:EtOAc = 1:1; UV)

$[\alpha]_D^{21}$= +275.6° (c = 1.300 g / 100 mL in $CHCl_3$)

IR (thin film) 2949, 2925, 2871, 2763, 1669, 1652, 1457, 1266, 761 cm^{-1}

^{1}H NMR (600 MHz, $CDCl_3$) δ 7.30 (d, *J* = 7.8 Hz, 1H), 7.15 (dd, *J* = 7.6, 7.6 Hz, 1H), 7.00 (d, *J* = 7.5 Hz, 1H), 6.48 (dd, *J* = 4.7, 1.9 Hz, 1H), 5.84 (s, 1H), 3.60 (d, *J* = 15.6 Hz, 1H), 3.42 (d, *J* = 15.7 Hz, 1H), 2.96 (d, *J* = 10.6 Hz, 1H), 2.74 (qd, *J* = 7.1, 7.1 Hz, 1H), 2.68 – 2.53 (m, 3H), 2.51 – 2.31 (m, 4H), 2.11 (m, 1H), 1.89 (m, 1H), 1.84 – 1.70 (m, 7H), 1.37-1.23 (m, 4H), 1.00 (m, 1H), 0.89 (d, *J* = 6.1 Hz, 3H) ppm

^{13}C NMR (150 MHz, $CDCl_3$) δ 199.2, 199.0, 167.8, 145.1, 143.8, 140.6, 137.6, 133.6, 129.7, 125.9, 125.5, 124.4, 65.7, 64.3, 57.0, 39.4, 39.1, 34.0, 32.9, 32.5, 32.2, 30.9, 28.9, 26.8, 23.8, 20.0, 19.6 ppm

HRMS (ESI) *m/z* $[M+H]^+$ calcd for $C_{27}H_{34}O_2N$: 404.2584; found: 404.2578

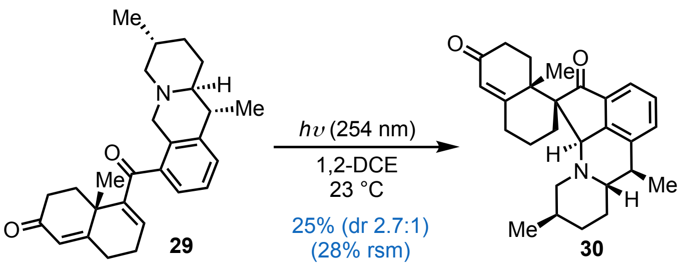

A solution of **29** (7.9 mg, 20 μmol, 1.0 equiv.) in 1,2-DCE (8 mL) was degassed by bubbling argon through the solution for 30 min. The solution was then photolyzed at 254 nm for 2 h at room temperature. Volatiles were evaporated to give a residue, which was purified by preparative thin layer chromatography (3% Et_3N in hexane:EtOAc = 2:1) to give a mixture of inseparable diastereomers (2.0 mg, 25%, dr 2.7:1) as a white solid and starting material **29** (2.2 mg, 28%). The structure of **30** was shown as established by X-ray crystallographic analysis.

Characterization of the 2.7:1 inseparable mixture:

R_f = 0.65 (3% Et_3N in hexane:EtOAc = 2:1; UV)

IR (thin film) 2926, 1706, 1667, 1611, 1450, 1377, 1332, 1171, 921 cm^{-1}

^{1}H NMR (600 MHz, CDCl3) δ 7.53 (d, *J* = 7.5 Hz, (1/3.7)1H), 7.51-7.47 (m, (2.7/3.7) 2H), 7.45 (d, *J* = 7.5 Hz, (1/3.7)1H), 7.36 (dd, *J* = 7.5, 7.5 Hz, (1/3.7)1H), 7.34 (dd, *J* = 7.6, 7.6 Hz, (2.7/3.7)1H), 5.82 (d, *J* = 2.1 Hz, (2.7/3.7)1H), 5.76 (d, *J* = 2.1 Hz, (1/3.7)1H), 4.22 (s, (2.7/3.7)1H), 3.40 (s, (1/3.7)1H), 3.34-3.20 (m, (2.7/3.7)1H + (1/3.7)2H), 2.96-2.83 (m, 2H), 2.80-2.52 (m, 4H), 2.49-2.05 (m, 6H), 2.01-1.82 (m, 5H), 1.79-1.71 (m, 1H), 1.71-1.48 (m, 4H), 1.40-1.20 (m, 5H), 1.12 (d, *J* = 7.1 Hz, (2.7/3.7)3H), 1.02-0.93 (m, (1/3.7)1H), 0.84 (d, *J* = 6.2 Hz, (1/3.7)3H), 0.48 (ddd, *J* = 13.3, 5.5, 2.7 Hz, (1/3.7)1H) ppm

^{13}C NMR (150 MHz, $CDCl_3$) δ 206.5, 204.8, 199.0, 198.7, 170.9, 168.2, 149.2, 149.2, 143.8, 137.7, 134.1, 132.5, 131.8, 129.2, 128.5, 127.6, 124.5, 124.5, 120.4, 120.0, 75.7, 67.8, 66.7, 66.3, 65.8, 65.8, 61.9, 55.1, 41.2, 40.8, 37.4, 34.4, 34.2, 32.7, 32.5, 31.4, 31.1, 31.0, 30.5, 29.8, 29.6, 28.5, 28.5, 25.6, 24.9, 23.7, 20.8, 20.6, 20.3, 19.8, 19.5, 19.0, 17.6, 13.7 ppm

HRMS (ESI) *m*/*z* $[M+H]^+$ calcd for $C_{27}H_{34}O_2N$: 404.2584; found: 404.2576

The inseparable mixture of diastereomers was recrystallized in diethyl ether and single-crystal structure determination was performed. The crystal used for the single-crystal X-ray diffraction was recovered and slightly washed by hexane, then NMR data of the crystal were collected. These results showed that compound **30** is the major diastereomer.

M.P.: 145-147 ˚C

¹H NMR (600 MHz, $CDCl_3$) δ 7.51 – 7.47 (m, 2H), 7.34 (dd, *J* = 7.6, 7.6 Hz, 1H), 5.82 (d, *J* = 2.1 Hz, 1H), 4.22 (s, 1H), 3.25 (dq, *J* = 7.1, 7.1 Hz, 1H), 2.95 – 2.85 (m, 2H), 2.79 – 2.70 (m, 1H), 2.64 – 2.52 (m, 2H), 2.45 (dd, *J* = 16.1, 6.7 Hz, 1H), 2.42 – 2.34 (m, 2H), 2.29 (ddd, *J* = 14.0, 14.0, 4.9 Hz, 1H), 2.10 (ddd, *J* = 13.5, 13.5, 6.2 Hz, 1H), 1.97 – 1.84 (m, 6H), 1.79 – 1.72 (m, 1H), 1.71 – 1.49 (m, 3H), 1.33 – 1.22 (m, 4H), 1.12 (d, *J* = 7.1 Hz, 3H) ppm

¹³C NMR (150 MHz, $CDCl_3$) δ 206.5, 198.7, 170.8, 149.2, 143.8, 132.5, 129.2, 127.6, 124.5, 120.4, 66.8, 65.9, 61.9, 55.1, 41.2, 34.2, 31.0, 30.5, 28.5, 28.5, 25.6, 25.0, 20.8, 20.6, 19.8, 17.6, 13.7 ppm

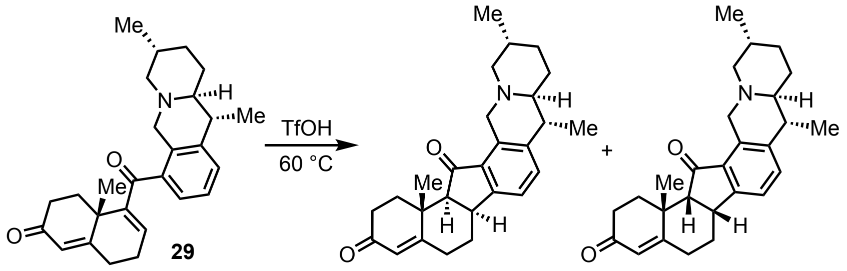

To **29** (8.0 mg, 20 μmol, 1.0 equiv.) in a microwave vial was added TfOH (0.5 mL), the vial was sealed and stirred for 20 h at 60 °C. The reaction mixture was cooled to room temperature and CH_2Cl_2 (1 mL) was added. To the mixture, saturated $NaHCO_3$ solution (4 mL) was carefully added at 0 ˚C, then $NaHCO_3$ solid was further carefully added until the pH reached 7~8. The resulting mixture was extracted 4 times with ethyl acetate, the combined organic phase was dried over anhydrous Na_2SO_4, filtered and concentrated. The residue was purified by preparative thin layer chromatography (3% Et_3N in hexane:EtOAc = 2:1) to give ketone **33** (4.6 mg, 58%) as a white solid and ketone **34** (1.8 mg, 23%) as a white solid.

Ketone **33**

M.P.: 125 ˚C (decomposed)

***R**f* = 0.26 (3% Et_3N in hexane:EtOAc = 2:1; UV)

$[\alpha]_D^{23}$= +43.1° (c = 0.430 g / 100 mL in CH_2Cl_2)

IR (thin film) 2947, 2925, 2751, 1692, 1667, 1606, 1485, 1101, 833 cm^{-1}

¹H NMR (600 MHz, $CDCl_3$) δ 7.50 (d, *J* = 8.0 Hz, 1H), 7.31 (d, *J* = 8.0 Hz, 1H), 5.86 (s, 1H), 4.62 (d, *J* = 17.2 Hz, 1H), 3.50 (ddd, *J* = 11.7, 7.8, 7.8 Hz, 1H), 3.40 (d, *J* = 17.1 Hz, 1H), 3.10 (d, *J* = 10.6 Hz, 1H), 2.81 – 2.60 (m, 4H), 2.59 (d, *J* = 8.1 Hz, 1H), 2.53 (ddd, *J* = 18.1, 13.3, 5.0 Hz, 1H), 2.43 (ddd, *J* = 17.8, 4.1, 4.1 Hz, 1H), 2.37 (m, 1H), 2.21-2.08(m,

2H), 1.92 – 1.70 (m, 5H), 1.38 – 1.24 (m, 4H), 1.08 – 0.96 (m, 4H), 0.92 (d, *J* = 6.4 Hz, 3H) ppm

^{13}C NMR (150 MHz, $CDCl_3$) δ 207.3, 198.7, 169.2, 156.0, 139.3, 134.3 (2 carbons overlapped), 132.4, 125.4, 122.4, 65.0, 64.0, 54.7, 54.2, 39.0, 38.5, 37.7, 37.3, 33.7, 32.9, 32.1, 30.9, 30.2, 24.2, 22.6, 19.8, 19.6 ppm

HRMS (ESI) *m/z* $[M+H]^+$ calcd for $C_{27}H_{34}O_2N$: 404.2584; found: 404.2579.

Ketone **34**

M.P.: 125 ˚C (decomposed)

R_f = 0.21 (3% Et_3N in hexane:EtOAc = 2:1; UV)

$[\alpha]_D^{22}$= –41.9° (c = 0.330 g / 100 mL in CH_2Cl_2)

IR (thin film) 2926, 2872, 2754, 1696, 1664, 1607, 1458, 1237, 829 cm^{-1}

^{1}H NMR (600 MHz, $CDCl_3$) δ 7.48 (d, *J* = 8.0 Hz, 1H), 7.29 (d, *J* = 8.0 Hz, 1H), 5.73 (s, 1H), 4.52 (d, *J* = 17.1 Hz, 1H), 3.79 (ddd, *J* = 7.4, 7.4, 2.9 Hz, 1H), 3.66 (ddd, *J* = 13.7, 13.7, 5.4 Hz, 1H), 3.34 (d, *J* = 17.2 Hz, 1H), 3.04 (d, *J* = 11.0 Hz, 1H), 2.80 – 2.65 (m, 2H), 2.64 (d, *J* = 7.1 Hz, 1H), 2.54 (dddd, *J* = 17.9, 5.5, 2.7, 0.9 Hz, 1H), 2.37 (m, 1H), 2.30 (ddd, *J* = 17.3, 4.1, 4.1 Hz, 1H), 2.12 (dd, *J* = 13.6, 3.0 Hz, 1H), 2.02 (dddd, *J* = 17.4, 12.8, 4.8, 2.1 Hz, 1H), 1.88 – 1.65 (m, 6H), 1.51 (d, *J* = 0.7 Hz, 3H), 1.29 (d, *J* = 6.9 Hz, 4H), 1.05 – 0.95 (m, 1H), 0.91 (d, *J* = 6.3 Hz, 3H) ppm

^{13}C NMR (150 MHz, $CDCl_3$) δ 206.2, 199.0, 167.8, 154.1, 139.4, 134.2, 133.9, 132.2, 125.4, 123.3, 65.0, 64.0, 55.8, 54.6, 39.1, 38.4, 37.0, 34.2, 32.9, 32.6, 32.0, 30.9, 28.1, 27.8, 27.5, 19.6, 19.6 ppm

HRMS (ESI) *m/z* $[M+H]^+$ calcd for $C_{27}H_{34}O_2N$: 404.2584; found: 404.2573

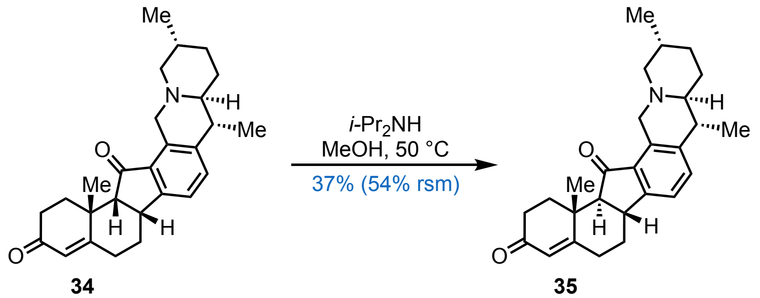

A solution of **34** (6.3 mg, 16 μmol, 1.0 equiv.) in a mixture of freshly distilled diisopropylamine (50 μL) and anhydrous methanol (3.3 mL) was stirred at 50 °C for 2 h. After the reaction was cooled down to room temperature, the solvent was evaporated in vacuo and the residue was purified by preparative thin layer chromatography (3% Et_3N in

hexane:EtOAc = 2:1) to give ketone **35** (2.3 mg, 37%) as a white solid and starting material **34** (3.4 mg, 54%).

Ketone **35**

M.P.: 125 ˚C (decomposed)

$\boldsymbol{R_f}$ = 0.26 (3% Et_3N in hexane:EtOAc = 2:1; UV)

$[\alpha]_D^{22}$ = +143.5° (c = 0.230 g / 100 mL in CH_2Cl_2)

IR (thin film) 2948, 2932, 2913, 2729, 1702, 1667, 1618, 1457, 1228, 1098, 841 cm^{-1}

^{1}H NMR (600 MHz, $CDCl_3$) δ 7.45 (d, *J* = 7.9 Hz, 1H), 7.27 (d, *J* = 8.1 Hz, 1H), 5.83 (s, 1H), 4.79 (d, *J* = 16.9 Hz, 1H), 3.36 – 3.17 (m, 2H), 3.07 (br s, 1H), 2.95 (ddd, *J* = 13.7, 5.0, 2.6 Hz, 1H), 2.75 (br s, 1H), 2.70 – 2.48 (m, 4H), 2.44 (dddd, *J* = 17.4, 4.6, 2.6, 1.0 Hz, 1H), 2.17 (d, *J* = 9.2 Hz, 1H), 2.11 (d, *J* = 11.9 Hz, 1H), 1.99 (ddd, *J* = 14.4, 14.4, 4.5 Hz, 1H), 1.90-1.71 (m, 4H), 1.68-1.55 (m, 1H), 1.41 – 1.22 (m, 7H), 1.06-0.96 (m, 1H), 0.91 (d, *J* = 6.0 Hz, 3H) ppm

^{13}C NMR (150 MHz, $CDCl_3$) δ 203.4, 199.3, 167.6, 150.4, 139.1, 134.3, 133.1, 131.3, 126.2, 121.2, 65.9, 65.0, 64.0, 53.7, 39.0, 38.9, 38.6, 35.0, 33.6, 33.3, 32.9, 32.0, 30.8, 28.9, 19.8, 19.6, 17.3 ppm

HRMS (ESI) *m*/*z* $[M+H]^+$ calcd for $C_{27}H_{34}O_2N$: 404.2584; found: 404.2576

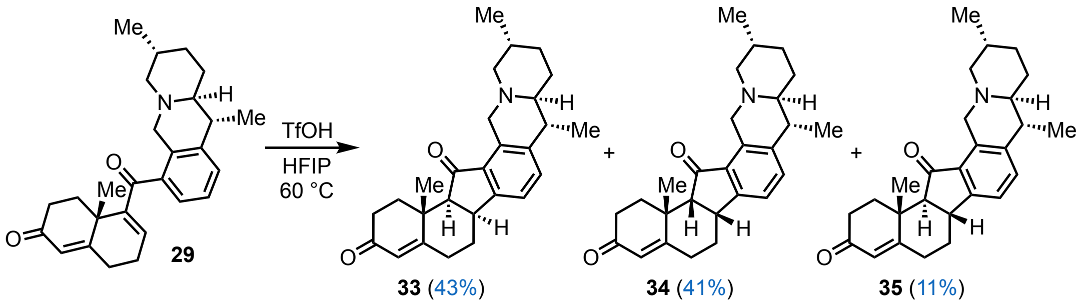

To a solution of **29** (8.0 mg, 20 μmol, 1.0 equiv.) in HFIP (0.4 mL) was added TfOH (40 μL, 23 equiv.). The solution was stirred for 20 h at 60 °C. The reaction mixture was cooled to 0 ˚C, saturated $NaHCO_3$ solution was carefully added until the pH reached 7~8. The resulting mixture was extracted 4 times with ethyl acetate, the combined organic phase was dried over anhydrous Na_2SO_4, filtered and concentrated. The residue was purified by preparative thin layer chromatography (3% Et_3N in hexane:EtOAc = 2:1) to give an inseparable mixture (4.3 mg, **35**:**33** = 1:4.1, based on ^{1}H NMR spectrum) of ketone **35** (11%, calculated yield) and ketone **33** (43%, calculated yield), and ketone **34** (3.3 mg, 41%) as a white solid.

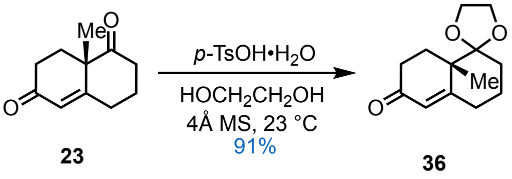

To a flame dried round bottom flask containing 1.8 g of activated molecular sieves (4Å MS beads) was added (*S*)-(+)-Wieland-Miescher ketone **23** (1.20 g, 6.73 mmol, 1.0 equiv.) and ethylene glycol (34 mL). It was stirred until all the (*S*)-(+)-Wieland-Miescher ketone dissolved. Subsequently, *p*-TsOH•H_2O (1.28 g, 6.73 mmol, 1.0 equiv.) was added at once, and the resulting solution was stirred at room temperature under argon atmosphere for 1 h. The mixture was poured into a cold saturated $NaHCO_3$ solution. The mixture was then extracted 3 times with EtOAc. The combined organic extracts were washed with brine and dried over anhydrous Na_2SO_4. The solvent was evaporated and the resulting residue was purified by column chromatography (hexane:EtOAc = 3:1) to afford **36** (1.36 g, 91%) as a colorless oil. The data fully match the reported data.[5]

$\boldsymbol{R_f}$ = 0.4 (hexane:EtOAc = 1:1; UV)

$[\boldsymbol{\alpha}]_D^{21}$ = +119.8º (c = 1.000 g / 100 mL in $CHCl_3$)

IR (thin film) 2949, 2882, 1668, 1619, 1456, 1332, 1280, 1174, 1098, 1020 cm^{-1}

^{1}H NMR (400 MHz, CDCl3) δ 5.81 (d, J = 2.0 Hz, 1H), 4.05 – 3.88 (m, 4H), 2.49 – 2.22 (m, 5H), 1.95 – 1.84 (m, 1H), 1.83 – 1.74 (m, 1H), 1.74 – 1.60 (m, 3H), 1.35 (s, 3H) ppm

^{13}C NMR (100 MHz, $CDCl_3$) δ 199.2, 167.7, 125.7, 112.3, 65.4, 65.1, 45.0, 33.9, 31.4, 30.0, 26.8, 21.7, 20.5 ppm

HRMS (ESI) *m*/*z* $[M+H]^+$ calcd for $C_{13}H_{19}O_3$: 223.1329; found: 223.1334

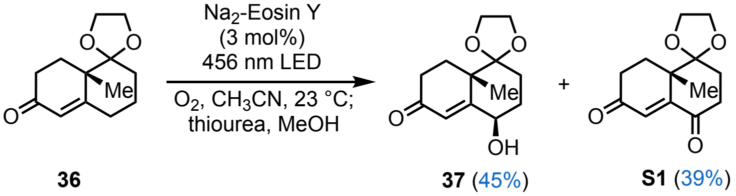

Note: The C–H oxidation follows a reported procedure by Yue et al.[6] but with a few alterations.

A 20 mL scintillation vial with a stir bar was charged with enone **36** (50 mg, 225 µmol, 1.0 equiv.), Na_2-Eosin Y (4.7 mg, 6.8 µmol, 0.03 equiv.), and anhydrous acetonitrile (12.5 mL). The mixture was bubbled with oxygen for 15 minutes. The reaction was stirred under an oxygen balloon (1 atm), exposed to a 456 nm LED lamp (Kessil PR160-456 nm), at room temperature for 16 h. The solvent was then evaporated in vacuo and subsequently charged with thiourea (22 mg, 289 µmol, 1.3 equiv.) and methanol (12.5 mL). The reaction was

stirred for an additional 4 h at room temperature. Following reaction completion, the solvent was evaporated in vacuo. Water (10 mL) was added and extracted with EtOAc (5x5 mL). The combined organic layer was washed with brine and dried over Na_2SO_4. The solvent was evaporated in vacuo and crude product was purified by column chromatography (hexane:EtOAc = 1:1) to afford alcohol **37** as a colorless oil (24 mg, 45 %) and diketone **S1** as a yellow solid (20.9 mg, 39 %).

37:

$\boldsymbol{R_f}$ = 0.16 (hexane:EtOAc = 1:1; UV)

$[\boldsymbol{\alpha}]_D^{21}$ = +6.3° (c = 1.000 g / 100 mL in $CHCl_3$)

IR (thin film) 3413, 2943, 2884, 1673, 1471, 1378, 1271, 1183, 1084, 875 cm^{-1}

^{1}H NMR (400 MHz, $CDCl_3$) δ 5.90 (s, 1H), 4.34 (dd, J = 3.1, 3.1 Hz, 1H), 4.04 – 3.90 (m, 4H), 2.57 – 2.38 (m, 2H), 2.37 – 2.23 (m, 2H), 2.05 – 1.89 (m, 2H), 1.72 – 1.57 (m, 3H), 1.55 (s, 3H) ppm

^{13}C NMR (100 MHz, $CDCl_3$) δ 200.1, 166.0, 128.0, 112.2, 71.8, 65.4, 65.0, 44.3, 33.9, 28.8, 27.8, 25.2, 22.2 ppm

HRMS (ESI) *m/z* $[M+H]^+$ calcd for $C_{13}H_{19}O_4$: 239.1278; found: 239.1282

S1:

$\boldsymbol{R_f}$ = 0.32 (hexane:EtOAc = 1:1; UV)

$[\boldsymbol{\alpha}]_D^{21}$ = –51.5° (c = 0.500 g / 100 mL in $CHCl_3$)

IR (thin film) 2960, 2891, 1683, 1450, 1328, 1227, 1207, 1128, 981, 937 cm^{-1}

^{1}H NMR (400 MHz, $CDCl_3$) δ 6.31 (s, 1H), 4.16 – 3.95 (m, 4H), 2.81 – 2.61 (m, 2H), 2.55 – 2.46 (m, 2H), 2.39 (td, J = 12.3, 7.9 Hz, 1H), 2.28 (ddd, J = 13.9, 12.5, 6.5 Hz, 1H), 2.00 (ddd, J = 13.9, 7.5, 2.2 Hz, 1H), 1.83 (dt, J = 12.9, 4.0 Hz, 1H), 1.35 (s, 3H) ppm

^{13}C NMR (100 MHz, $CDCl_3$) δ 200.0, 199.4, 156.6, 127.8, 110.3, 65.7, 65.5, 44.5, 37.0, 33.7, 27.6, 27.0, 21.0 ppm

HRMS (ESI) *m/z* $[M+H]^+$ calcd for $C_{13}H_{17}O_4$: 237.1121; found: 237.1124

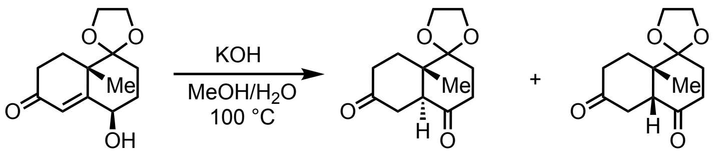

37 **38** (56%) **S2** (21%)

To a solution of **37** (135 mg, 0.567 mmol, 1.0 equiv.) in methanol (14 mL) was added potassium hydroxide (115 mg, 2.05 mmol, 3.6 equiv.) in methanol (6 mL) and water (40 mL). The resulting mixture was bubbled with argon for 25 min. After heating the yellow mixture for 1 h at 100 ˚C and subsequently saturating the reaction mixture with sodium

chloride at room temperature, the resulting mixture was extracted 3 times with EtOAc. The combined organic layers were washed with water (3x10 mL), the combined aqueous layers were saturated with sodium chloride and extracted 3 times with EtOAc. The whole organic extracts were dried over anhydrous Na_2SO_4, filtered and concentrated under reduced pressure. The resulting residue was purified by column chromatography (hexane:EtOAc = gradually from 3/1 to 1/1) to afford diketone **38** (76 mg, 56%) as a white solid and its minor diastereomer **S2** (29 mg, 21%) as a white powder.

38:

M.P.: 123-125˚C

R_f = 0.5 (hexane:EtOAc = 1:1; visualized with CAM stain)

$[\alpha]_D^{21}$= –24.0° (c = 1.000 g / 100 mL in $CHCl_3$)

IR (thin film) 2943, 2963, 2892, 1713, 1480, 1151, 1031, 920 cm^{-1}

^{1}H NMR (400 MHz, $CDCl_3$) δ 4.17 – 3.90 (m, 4H), 3.15 (dd, J = 13.0, 4.4 Hz, 1H), 2.70 – 2.56 (m, 1H), 2.49 (dd, J = 15.6, 12.9 Hz, 1H), 2.44 – 2.29 (m, 4H), 2.20 – 2.04 (m, 2H), 1.99 (ddd, J = 13.8, 7.5, 1.8 Hz, 1H), 1.77 (ddd, J = 13.1, 5.7, 3.0 Hz, 1H), 1.14 (s, 3H) ppm

^{13}C NMR (100 MHz, $CDCl_3$) δ 210.7, 208.8, 110.7, 65.6, 65.5, 51.8, 45.0, 38.2, 36.9, 36.7, 30.6, 29.8, 15.1 ppm

HRMS (ESI) m/z $[M+H]^+$ calcd for $C_{13}H_{19}O_4$: 239.1278; found: 239.1273

S2:

R_f = 0.38 (hexane:EtOAc = 1:1; visualized with CAM stain)

$[\alpha]_D^{21}$= +27.1° (c = 1.000 g / 100 mL in $CHCl_3$)

IR (thin film) 2973, 2958, 2945, 2889, 1720, 1706, 1158, 1001, 910 cm^{-1}

^{1}H NMR (400 MHz, $CDCl_3$) δ 4.18 – 4.02 (m, 4H), 3.12-3.05 (m, 1H), 2.80 (ddd, J = 16.3, 2.6, 1.4 Hz, 1H), 2.73 – 2.62 (m, 1H), 2.45 – 2.32 (m, 3H), 2.27 (dd, J = 16.2, 6.0 Hz, 1H), 2.13 – 1.96 (m, 2H), 1.85 – 1.68 (m, 2H), 1.26 (s, 3H) ppm

^{13}C NMR (100 MHz, $CDCl_3$) δ 210.1, 208.4, 110.6, 65.7, 65.3, 53.1, 45.1, 37.7, 36.8, 36.6, 30.2, 29.4, 17.3 ppm

HRMS (ESI) m/z $[M+H]^+$ calcd for $C_{13}H_{19}O_4$: 239.1278; found: 239.1277

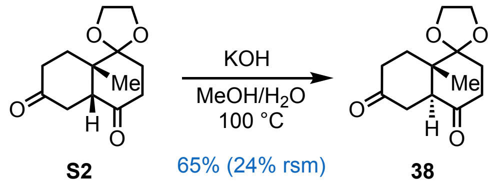

Note: The accumulated **S2** was further isomerized to **38** to avoid material loss.

To a solution of **S2** (0.17 g, 0.71 mmol, 1.0 equiv.) in methanol (20 mL) was added potassium hydroxide (144 mg, 2.57 mmol, 3.6 equiv.) in methanol (5 mL) and water (50 mL). The resulting mixture was bubbled with argon for 25 min. After heating the yellow mixture for 1 h at 100 ˚C, the reaction was cooled to room temperature. The reaction mixture was saturated with sodium chloride at room temperature, the resulting mixture was extracted 3 times with EtOAc. The combined organic layers were washed with water (3x10 mL), the combined aqueous layers were saturated with sodium chloride and extracted 3 times with EtOAc. The whole organic extracts were dried over anhydrous Na_2SO_4, filtered and concentrated under reduced pressure. The resulting residue was purified by column chromatography (hexane:EtOAc = gradually from 3/1 to 1/1) to afford diketone **38** (0.11 g, 65%) as a white solid and starting material **S2** (0.04 g, 24%) as a white powder.

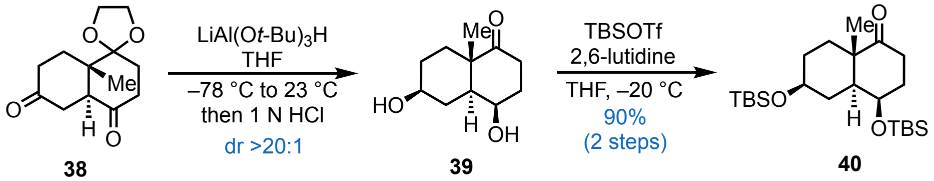

Lithium tri-*i*-butoxyaluminohydride (1 M in THF, 10 mL, 10 mmol, 5.0 equiv.) was added over 30 min to a solution of diketone **38** (0.48 g, 2.0 mmol, 1.0 equiv.) in THF (20 mL) at –78 ˚C. After stirring for 4 h at –78 ˚C, the reaction mixture was allowed to warm to room temperature very slowly (over 4 h) and further stirred for 17 h at room temperature. The resulting mixture was cooled to 0 ˚C and 1 N HCl (60 mL) was added slowly at 0 ˚C. It was then allowed to warm to room temperature and stirred at room temperature for 4 h. The resulting mixture was cooled to 0 ˚C, saturated $NaHCO_3$ solution (20 mL) was slowly added and $NaHCO_3$ solid was further carefully added until pH reached 7~8. After saturating the resulting mixture with sodium chloride at room temperature, the mixture was extracted 10 times with EtOAc. The combined organic layers were dried over anhydrous Na_2SO_4, filtered and concentrated under reduced pressure to afford crude diol **39** (0.43 g) as a white solid that was used in the next step without further purification.

(Notice: extracting 10 times with EtOAc is necessary to sufficiently get the diol out from aqueous layer)

To a solution of above crude diol **39** (0.43 g, 2.2 mmol, 1.0 equiv.) in THF (22 mL) was added 2,6-lutidine (1 mL, 8.6 mmol, 3.9 equiv.) and TBSOTf (1.5 mL, 6.5 mmol, 3.0 equiv.) at –20 °C. After stirring for 1 h at –20 °C, saturated NH_4Cl solution was added, and the mixture was extracted 3 times with EtOAc. The combined organic phases were washed with brine, dried over anhydrous Na_2SO_4, filtered and concentrated under reduced pressure. The resulting residue was purified by column chromatography (gradually from hexane only

to hexane:Et_2O = 9/1) to afford silyl ether **40** (0.77 g, 90% for 2 steps, based on diketone **38**) as a white solid.

***R*$_f$** = 0.15 (hexane:Et_2O = 25:1; visualized with CAM stain)

$[\alpha]_D^{23}$ = –72.2° (c = 0.550 g / 100 mL in $CHCl_3$)

IR (thin film) 2950, 2928, 2856, 1708, 1471, 1385, 1251, 1067, 794 cm^{-1}

^{1}H NMR (400 MHz, $CDCl_3$) δ 3.84 (ddd, *J* = 2.8, 2.8, 2.8 Hz, 1H), 3.54 (dddd, *J* = 10.5, 10.5, 4.8, 4.8 Hz, 1H), 3.06 (ddd, *J* = 14.1, 14.1, 6.1 Hz, 1H), 2.11 (ddd, *J* = 13.9, 4.7, 2.5 Hz, 1H), 2.02 (dddd, *J* = 14.0, 5.8, 2.7, 2.7 Hz, 1H), 1.92 – 1.72 (m, 3H), 1.66 – 1.36 (m, 5H), 1.34 (s, 3H), 0.93 (s, 9H), 0.89 (s, 9H), 0.09 (s, 3H), 0.08 (s, 3H), 0.06 (s, 3H), 0.06 (s, 3H) ppm

^{13}C NMR (100 MHz, $CDCl_3$) δ 216.3, 71.9, 70.7, 47.8, 47.5, 35.4, 35.0, 33.2, 32.4, 31.2, 25.9, 25.8, 19.4, 18.2, 18.0, -4.5, -4.6, -4.7, -4.9 ppm

HRMS (ESI) *m/z* $[M+H]^+$ calcd for $C_{23}H_{47}O_3Si_2$: 427.3058; found: 427.3053

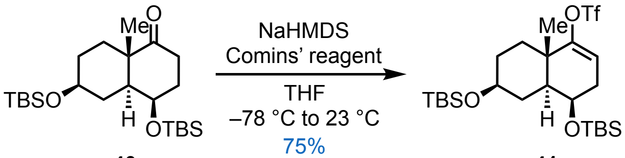

40 41

To a solution of **40** (571 mg, 1.34 mmol, 1.0 equiv.) in THF (8.9 mL) was added dropwise NaHMDS (1 M in THF, 2.4 mL, 2.4 mmol, 1.8 equiv.) at –78 °C. After stirring 80 min at –78 °C, Comins' reagent (690 mg, 1.76 mmol, 1.3 equiv.) in THF (3.5 mL) was added dropwise at –78 °C. After stirring 30 min at –78 °C, the reaction mixture was allowed to warm to room temperature and was further stirred for 2.5 h at room temperature. Saturated NH_4Cl solution was added, and the mixture was extracted three times with EtOAc. The combined organic phases were washed with brine, dried over anhydrous Na_2SO_4, filtered and concentrated under reduced pressure. The residue was purified by flash column chromatography (from hexane:CH_2Cl_2 = 10:1 to hexane:Et_2O = 9:1) to afford vinyl triflate **41** (560 mg, 75%) as a colorless oil. A small amount of the starting material **40** (80 mg, 14%) was recovered.

***R*$_f$** = 0.26 (hexane:CH_2Cl_2 = 10:1; visualized with CAM stain)

$[\alpha]_D^{23}$ = –21.2° (c = 0.540 g / 100 mL in $CHCl_3$)

IR (thin film) 2952, 2929, 2857, 1680, 1472, 1412, 1092, 957, 835 cm^{-1}

^{1}H NMR (600 MHz, $CDCl_3$) δ 5.52 (dd, *J* = 5.1, 2.8 Hz, 1H), 3.86 (d, *J* = 4.9 Hz, 1H), 3.64 (dddd, *J* = 10.6, 10.6, 5.0, 5.0 Hz, 1H), 2.55 (ddd, *J* = 18.5, 5.3, 2.9 Hz, 1H), 2.21 (dd, *J* = 18.6, 5.1 Hz, 1H), 1.84-1.74 (m, 2H), 1.71 (ddd, *J* = 13.2, 3.6, 3.6 Hz, 1H), 1.61 – 1.47

(m, 3H), 1.41-1.31 (m, 4H), 0.89 (s, 9H), 0.88 (s, 9H), 0.06 (s, 3H), 0.06 (s, 3H), 0.05 (s, 3H), 0.04 (s, 3H) ppm

^{13}C NMR (150 MHz, $CDCl_3$) δ 156.5, 118.4 (q, $J_{C\text{-}F}$ = 317.5 Hz), 112.1, 71.9, 69.3, 45.8, 37.3, 35.5, 34.5, 34.4, 31.0, 25.9, 25.7, 19.0, 18.2, 17.9, -4.5, -4.6, -4.7, -4.9 ppm

HRMS (ESI) *m/z* $[M+H]^+$ calcd for $C_{24}H_{46}O_5F_3SSi_2$: 559.2551; found: 559.2548

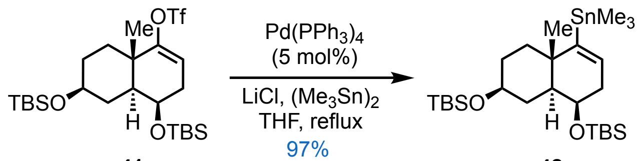

41 **42**

In a glove box, to a round bottom flask containing vinyl triflate **41** (710 mg, 1.27 mmol, 1.0 equiv.) was added anhydrous LiCl (269 mg, 6.35 mmol, 5.0 equiv.), $Pd(PPh_3)_4$ (73 mg, 0.063 mmol, 0.05 equiv.), THF (25.4 mL) and hexamethylditin (1.1 mL, 5.3 mmol, 4.2 equiv.). The flask was taken out from glove box and an argon flushed condenser was attached. After stirring for 16 h at 80 °C, the reaction was cooled to room temperature, diluted with Et_2O, filtered through a short pad of celite, and eluted with Et_2O. The filtrate was washed twice with brine. The organic phase was dried over anhydrous Na_2SO_4, filtered and concentrated under reduced pressure. The residue was purified by flash column chromatography (hexane:CH_2Cl_2 = 10:1) to afford vinyl stannane **42** (710 mg, 97%) as a white solid.

R_f = 0.56 (hexane:CH_2Cl_2 = 10:1; visualized with CAM stain)

$[\alpha]_D^{23}$ = –19.3° (c = 1.000 g / 100 mL in $CHCl_3$)

IR (thin film) 2953, 2928, 2856, 1471, 1251, 1090, 1003, 680 cm^{-1}

^{1}H NMR (600 MHz, $CDCl_3$) δ 5.55 (dd, *J* = 3.3, 3.3 Hz, 1H), 3.88 (dd, *J* = 6.1, 2.5 Hz, 1H), 3.62 (dddd, *J* = 10.7, 10.7, 4.9, 4.9 Hz, 1H), 2.47 (ddd, *J* = 18.9, 6.1, 3.0 Hz, 1H), 2.11 (dd, *J* = 18.9, 4.0 Hz, 1H), 1.82 – 1.67 (m, 2H), 1.63 – 1.52 (m, 1H), 1.48 (dddd, *J* = 12.6, 4.8, 2.3, 2.3 Hz, 1H), 1.40 (ddd, *J* = 12.7, 3.5, 3.5 Hz, 1H), 1.32 (ddd, *J* = 13.1, 2.5, 2.5 Hz, 1H), 1.22 (ddd, *J* = 13.2, 13.2, 3.8 Hz, 1H), 1.12 (s, 3H), 0.89 (s, 9H), 0.87 (s, 9H), 0.12 (s, 9H), 0.07 (s, 3H), 0.06 (s, 3H), 0.02 (s, 3H), 0.02 (s, 3H) ppm

^{13}C NMR (150 MHz, $CDCl_3$) δ 152.7, 131.6, 72.8, 70.5, 45.0, 41.5, 38.7, 37.5, 37.1, 32.0, 26.0, 25.9, 21.6, 18.3, 18.0, -4.5, -4.5, -4.6, -5.0, -7.5 ppm

HRMS (ESI) *m/z* $[M+H]^+$ calcd for $C_{26}H_{55}O_2Si_2Sn$: 575.2757; found: 575.2749

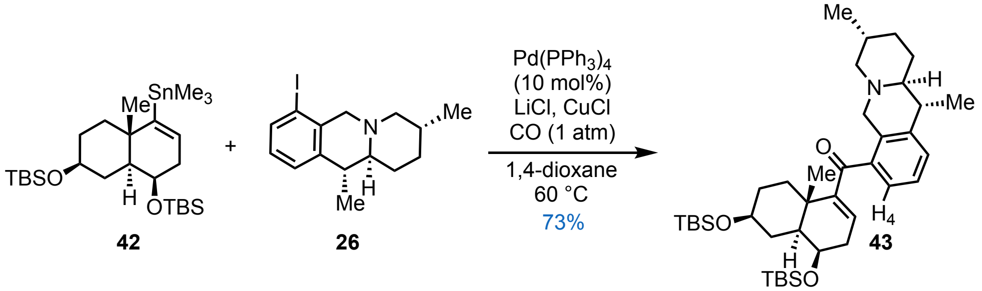

In a glove box, to an oven dried microwave vial was charged with anhydrous LiCl (187 mg, 4.41 mmol, 8.6 equiv.), $Pd(PPh_3)_4$ (59.4 mg, 0.051 mmol, 0.1 equiv.) and CuCl (254 mg, 2.57 mmol, 5.0 equiv.). Subsequently, iodide **26** (175 mg, 0.513 mmol, 1.0 equiv.), vinyl stannane **42** (442 mg, 0.771 mmol, 1.5 equiv.) and 1,4-dioxane (10.5 mL) were added. The vial was sealed and taken out from the glove box. The vial was charged with a CO balloon and the reaction mixture was bubbled with CO for 30 min. The reaction mixture was heated to 60 °C for 24 h with the CO balloon (1 atm). The reaction mixture was then cooled to room temperature, diluted with EtOAc and a mixture of brine and 5% aqueous NH_4OH (5/1, v/v) was added. The organic phase was separated, and the aqueous phase was then further extracted 3 times with EtOAc. The combined organic layers were washed twice with brine, dried over anhydrous Na_2SO_4, and concentrated to a residue. The residue was purified by flash column chromatography (hexane:Et_3N = from 100:1 to 100:3) to afford enone **43** (244 mg, 73%, based on **26**) as a white solid.

$\boldsymbol{R_f}$ = 0.3 (hexane:Et_3N = 100:3; UV)

$[\alpha]_D^{22}$ = +43.4° (c = 0.500 g / 100 mL in $CHCl_3$)

IR (thin film) 2952, 2928, 2856, 1736, 1651, 1252, 1462, 1361, 1252, 1014, 835 cm^{-1}

^{1}H NMR (400 MHz, $CDCl_3$) δ 7.28 (d, *J* = 7.7 Hz, 1H), 7.13 (dd, *J* = 7.7, 7.6 Hz, 1H), 7.03 (d, *J* = 7.4 Hz, 1H), 6.16 (dd, *J* = 3.8, 3.8 Hz, 1H), 3.90 (dd, *J* = 5.3, 1.8 Hz, 1H), 3.72-3.60 (m, 2H), 3.44 (d, *J* = 16.0 Hz, 1H), 2.97 (d, *J* = 8.5 Hz, 1H), 2.80-2.67 (m, 1H), 2.59 (ddd, *J* = 20.4, 6.1, 3.5 Hz, 1H), 2.32 (ddd, *J* = 13.2, 3.6, 3.6 Hz, 1H), 2.23 – 2.06 (m, 2H), 1.95-1.49 (m, 11 H), 1.41 (ddd, *J* = 13.0, 2.5, 2.5 Hz, 1H), 1.37-1.22 (m, 4H), 1.12 (ddd, *J* = 13.3, 13.3, 4.0 Hz, 1H), 1.07-0.94 (m, 1H), 0.94-0.88 (m, 12H), 0.86 (s, 9H), 0.07 (s, 3H), 0.07 (s, 3H), 0.04 (s, 3H), 0.01 (s, 3H) ppm

^{13}C NMR (100 MHz, $CDCl_3$) δ 199.5, 149.1, 140.9, 140.4, 138.2, 134.0, 129.5, 126.4, 125.3, 72.5, 69.7, 65.8, 64.2, 57.1, 45.3, 39.4, 36.4, 36.1, 35.9, 35.2, 33.0, 32.3, 31.7, 31.0, 25.9, 25.8, 20.6, 20.1, 19.6, 18.3, 17.9, -4.5, -4.5, -4.7, -4.9 ppm

HRMS (ESI) *m/z* $[M+H]^+$ calcd for $C_{39}H_{66}O_3NSi_2$: 652.4576; found: 652.4588

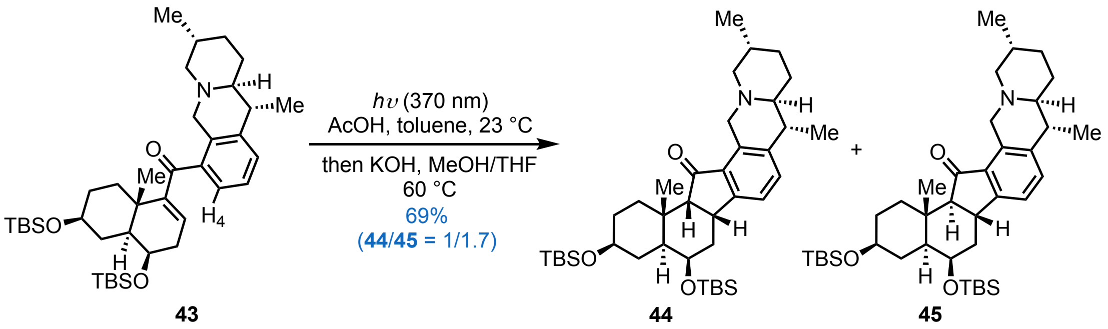

A solution of **43** (10.1 mg, 15.5 μmol, 1.0 equiv.) in toluene (1 mL) and AcOH (50 μL, 0.87 mmol, 56.1 equiv.) was degassed by bubbling argon through the solution for 30 min. The solution was then photolyzed at 370 nm (Kessil PR160L-370nm Gen 2) for 75 min at room temperature. The photo reactor was turned off. MeOH (1 mL), potassium hydroxide (103 mg, 1.84 mmol, 118.7 equiv.) and THF (0.5 mL) were added to the mixture and the solution was stirred at 60 °C for 2 h. The mixture was cooled to room temperature and brine (2 mL) was added. The resulting mixture was extracted four times with ethyl acetate, and the combined organic phase was dried over anhydrous Na_2SO_4, filtered and concentrated. The residue was purified by preparative thin layer chromatography (3%Et_3N in hexane:EtOAc = 15:1) to give ketone **45** (4.3 mg, 43%) as a white solid and ketone **44** (2.6 mg, 26%) as a white solid.

44:

R_f = 0.5 (3% Et_3N in hexane:EtOAc = 15:1; UV)

$[\alpha]_D^{22}$= +28.0° (c = 0.200 g / 100 mL in CH_2Cl_2)

IR (thin film) 2951, 2927, 2855, 1733, 1706, 1461, 1252, 1085, 872, 772 cm^{-1}

^{1}H NMR (600 MHz, $CDCl_3$) δ 7.35 (d, *J* = 7.8 Hz, 1H), 7.18 (d, *J* = 7.8 Hz, 1H), 4.42 (d, *J* = 16.9 Hz, 1H), 3.75 – 3.64 (m, 2H), 3.60 (ddd, *J* = 11.5, 5.5, 5.5 Hz, 1H), 3.32 (d, *J* = 16.9 Hz, 1H), 3.02 (d, *J* = 10.2 Hz, 1H), 2.96 (ddd, *J* = 13.8, 13.8, 3.8 Hz, 1H), 2.76 – 2.65 (m, 1H), 2.53 (d, *J* = 5.4 Hz, 1H), 2.17 – 2.06 (m, 2H), 1.88 – 1.68 (m, 6H), 1.67 – 1.56 (m, 2H), 1.51 – 1.43 (m, 1H), 1.43 – 1.19 (m, 9H), 1.07 – 0.81 (m, 22H), 0.06 (s, 3H), 0.06 (s, 3H), 0.06 (s, 3H), 0.04 (s, 3H) ppm

^{13}C NMR (150 MHz, $CDCl_3$) δ 206.7, 155.2, 138.8, 134.3, 132.5, 131.5, 122.3, 72.5, 71.7, 65.2, 64.0, 61.2, 54.9, 44.0, 42.4, 39.1, 36.4, 36.1, 35.4, 34.2, 33.0, 32.1, 31.4, 30.9, 26.0, 25.8, 24.0, 19.8, 19.6, 18.3, 18.0, -4.5, -4.6, -4.7, -4.9 ppm

HRMS (ESI) *m/z* $[M+H]^+$ calcd for $C_{39}H_{66}O_3NSi_2$: 652.4576; found: 652.4589

45:

R_f = 0.55 (3% Et_3N in hexane:EtOAc = 15:1; UV)

$[\alpha]_D^{22}$ = +23.5° (c = 0.200 g / 100 mL in CH_2Cl_2)

IR (thin film) 2951, 2928, 2856, 1709, 1472, 1461, 1254, 1142, 1095, 1078, 835 cm^{-1}

^{1}H NMR (600 MHz, $CDCl_3$) δ 7.40 (d, *J* = 7.9 Hz, 1H), 7.19 (d, *J* = 7.9 Hz, 1H), 4.81 (d, *J* = 16.9 Hz, 1H), 3.95 (ddd, *J* = 2.6, 2.6, 2.6 Hz, 1H), 3.63 (dddd, *J* = 10.6, 10.6, 4.9, 4.9 Hz, 1H), 3.54 (dd, *J* = 10.8, 10.8 Hz, 1H), 3.24 (d, *J* = 16.9 Hz, 1H), 3.05 (d, *J* = 8.8 Hz, 1H), 2.73 (br s, 1H), 2.50 (ddd, *J* = 13.3, 3.7, 3.7 Hz, 1H), 2.39 (ddd, *J* = 12.7, 3.0, 3.0 Hz, 1H), 2.09 (d, *J* = 13.2 Hz, 1H), 1.91 (d, *J* = 8.9 Hz, 1H), 1.86 – 1.54 (m, 8H), 1.53 – 1.46 (m, 1H), 1.39 – 1.23 (m, 4H), 1.22-1.14 (m, 4H), 1.11 (ddd, *J* = 12.9, 2.5, 2.5 Hz, 1H), 1.04-0.75 (m, 22H), 0.09 (s, 3H), 0.07 (s, 3H), 0.07 (s, 3H), 0.07 (s, 3H) ppm

^{13}C NMR (150 MHz, $CDCl_3$) δ 205.4, 152.2, 138.4, 134.1, 132.6, 131.7, 120.8, 72.7, 72.5, 67.8, 65.0, 64.0, 53.8, 49.5, 39.0, 37.5, 37.1, 36.4, 35.9, 33.9, 33.0, 31.9, 31.4, 30.7, 25.9, 25.8, 19.8, 19.6, 18.2, 17.9, 15.6, -4.4, -4.5, -4.6, -4.9 ppm

HRMS (ESI) *m/z* $[M+H]^+$ calcd for $C_{39}H_{66}O_3NSi_2$: 652.4576; found: 652.4582

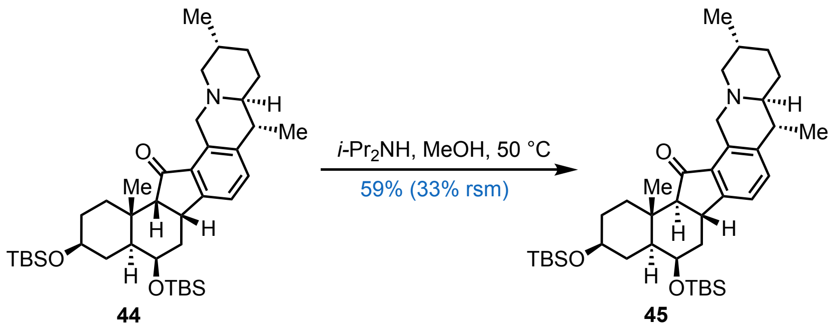

A solution of **44** (8.0 mg, 12 µmol, 1.0 equiv.) in a mixture of freshly distilled diisopropylamine (0.11 mL) and anhydrous methanol (1.1 mL) was stirred at 50 °C for 2 h. After the reaction was cooled down to room temperature, the solvent was evaporated in vacuo and the residue was purified by preparative thin layer chromatography (3% Et_3N in hexane:EtOAc = 15:1) to give ketone **45** (4.7 mg, 59%) as a white solid and starting material **44** (2.6 mg, 33%).

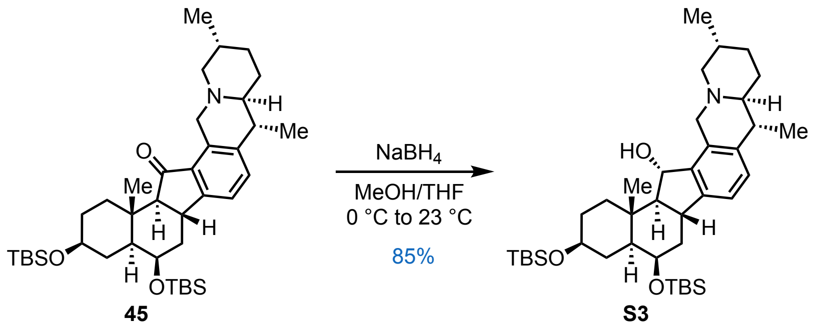

To a solution of ketone **45** (13.8 mg, 21.2 µmol, 1.0 equiv.) in THF (1 mL) and MeOH (1 mL) was added $NaBH_4$ (20 mg, 0.53 mmol, 25.0 equiv.) at 0 °C. After stirring for 2 h at

0 °C, the reaction mixture was allowed to warm to room temperature and was further stirred for 12 h at room temperature. Saturated $NaHCO_3$ solution was added, and the mixture was extracted three times with EtOAc. The combined organic phases were dried over anhydrous Na_2SO_4, filtered and concentrated under reduced pressure. The residue was purified by preparative thin layer chromatography (3% Et_3N in hexane:EtOAc = 15:1) to give alcohol **S3** (11.8 mg, 85%) as a white solid.
R_f = 0.35 (3% Et_3N in hexane:EtOAc = 15:1; UV)
$[\alpha]_D^{23}$ = +28.9° (c = 0.300 g / 100 mL in $CHCl_3$)
IR (thin film) 3276, 2952, 2928, 2856, 1472, 1460, 1254, 1085, 1045, 835, 774 cm^{-1}
^{1}H NMR (600 MHz, $CDCl_3$) δ 7.10 (d, *J* = 7.8 Hz, 1H), 6.94 (d, *J* = 7.8 Hz, 1H), 5.14 (dd, *J* = 8.5, 8.5 Hz, 1H), 3.96 (d, *J* = 15.5 Hz, 1H), 3.89 (ddd, *J* = 2.6, 2.6, 2.6 Hz, 1H), 3.63 (dddd, *J* = 10.6, 10.6, 4.9, 4.9 Hz, 1H), 3.39 (d, *J* = 15.5 Hz, 1H), 3.13 – 2.98 (m, 2H), 2.70 (dq, *J* = 7.2, 7.2 Hz, 1H), 2.28 (ddd, *J* = 12.8, 3.2, 3.2 Hz, 1H), 2.09 (dddd, *J* = 13.3, 3.1, 3.1, 3.1 Hz, 1H), 1.95 – 1.39 (m, 11H), 1.36 – 1.23 (m, 5H), 1.18 (s, 3H), 1.13 (ddd, *J* = 12.9, 2.6, 2.6 Hz, 1H), 1.04 – 0.80 (m, 22H), 0.07 (s, 3H), 0.07 (s, 3H), 0.06 (s, 3H), 0.06 (s, 3H) ppm
^{13}C NMR (150 MHz, $CDCl_3$) δ 141.8, 140.5, 138.0, 132.0, 127.2, 120.1, 75.5, 73.0, 72.6, 70.8, 66.0, 64.4, 56.2, 49.1, 39.7, 39.4, 38.0, 36.3, 36.1, 35.3, 33.1, 32.3, 31.6, 30.9, 25.9, 25.8, 20.5, 19.7, 18.3, 17.9, 16.2, -4.5, -4.5, -4.6, -4.9 ppm
HRMS (ESI) *m/z* $[M+H]^+$ calcd for $C_{39}H_{68}O_3NSi_2$: 654.4732; found: 654.4745

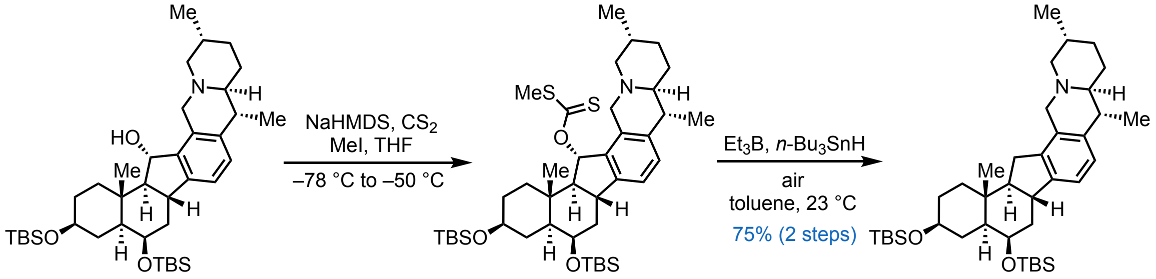

S3 **S4** **46**

To a solution of alcohol **S3** (7.9 mg, 12 μmol, 1.0 equiv.) in THF (0.3 mL) was added dropwise NaHMDS (1 M in THF, 0.12 mL, 0.12 mmol, 10.0 equiv.) at –78 °C. After stirring 30 min at –78 °C, carbon disulfide (19 μL, 0.32 mmol, 26.7 equiv.) was added dropwise at –78 °C. After stirring 5 min at –78 °C, the reaction mixture was allowed to warm to –50 °C and was further stirred at –50 °C for 1 h. Iodomethane (23 uL, 0.37 mmol, 30.8 equiv.) was added dropwise at –50 °C and the mixture was stirred at –50 °C for 1 h. Saturated $NaHCO_3$ solution was added at –50 °C. The mixture was warmed to room temperature and extracted three times with EtOAc. The combined organic phases were

dried over anhydrous Na_2SO_4, filtered and concentrated under reduced pressure. The residue was quickly purified by preparative thin layer chromatography (3% Et_3N in hexane) to give xanthate ester **S4** (8.3 mg) which was used immediately for next step. **(Note: Due to the instability of xanthate ester S4, the characterization data were not collected.)**

Under argon atmosphere, to a solution of above xanthate ester **S4** (8.3 mg, 11 μmol, 1.0 equiv.) in dry toluene (0.56 mL) was added tributyltin hydride (25 μL, 93 μmol, 8.5 equiv.) and triethylborane (1.0 M in hexanes, 12 μL, 12 μmol, 1.1 equiv.) at room temperature. The argon balloon was removed, and air (5 μL) was injected through the septum stopper by a microsyringe. After stirring for 2 h at room temperature, air (5 μL) was injected through the septum stopper by a microsyringe again. The reaction mixture was further stirred at room temperature for 2 h. Volatiles were evaporated to give a residue. The residue was purified by preparative thin layer chromatography (3% Et_3N in hexane) to give amine **46** (5.8 mg, 75% for 2 steps, based on alcohol **S3**) as a white solid.

R_f = 0.55 (hexane:Et_3N = 100:3; UV)

$[\alpha]_D^{23}$= +29.0° (c = 0.580 g / 100 mL in $CHCl_3$)

IR (thin film) 2951, 2928, 2856, 1472, 1461, 1361, 1253, 1078, 1049, 1006, 835 cm^{-1}

^{1}H NMR (600 MHz, $CDCl_3$) δ 7.05 (d, *J* = 7.7 Hz, 1H), 6.94 (d, *J* = 7.8 Hz, 1H), 3.91 (ddd, *J* = 2.6, 2.6, 2.6 Hz, 1H), 3.70 (d, *J* = 15.0 Hz, 1H), 3.63 (dddd, *J* = 10.7, 10.7, 4.9, 4.9 Hz, 1H), 3.30 – 3.14 (m, 2H), 3.01 (dd, *J* = 7.7, 1.9 Hz, 1H), 2.70 (dq, *J* = 7.2, 7.2 Hz, 1H), 2.53 (dd, *J* = 14.4, 6.8 Hz, 1H), 2.44 (dd, *J* = 13.7, 12.5 Hz, 1H), 2.28 (ddd, *J* = 12.9, 3.2, 3.2 Hz, 1H), 2.11 (dddd, *J* = 13.3, 3.1, 3.1, 3.1 Hz, 1H), 1.90 – 1.64 (m, 6H), 1.63 – 1.42 (m, 5H), 1.38 – 1.23 (m, 4H), 1.18 – 1.05 (m, 5H), 0.90 (d, *J* = 4.2 Hz, 22H), 0.14 – -0.00 (m, 12H) ppm

^{13}C NMR (150 MHz, $CDCl_3$) δ 143.9, 139.5, 136.7, 130.2, 124.8, 120.0, 73.2, 72.9, 65.5, 64.4, 61.3, 56.3, 49.0, 39.2, 39.1, 38.6, 37.9, 36.1, 35.8, 33.1, 31.9, 31.6, 30.8, 28.7, 25.9, 25.8, 19.7, 19.5, 18.3, 18.0, 15.6, -4.5, -4.5, -4.5, -4.9 ppm

HRMS (ESI) *m/z* $[M+H]^+$ calcd for $C_{39}H_{68}O_2NSi_2$: 638.4783; found: 638.4789

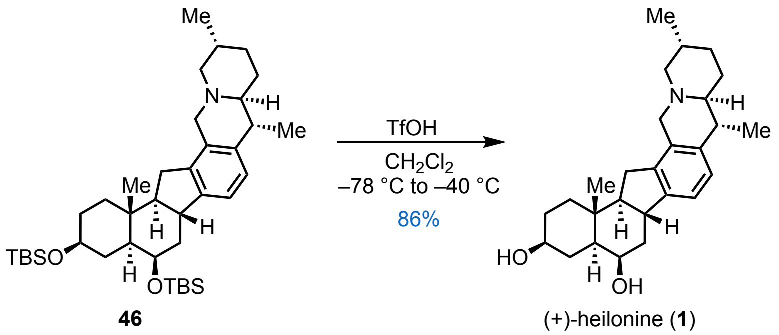

To a solution of amine **46** (5.8 mg, 9.1 μmol, 1.0 equiv.) in CH_2Cl_2 (0.91 mL) was added dropwise trifluoromethanesulfonic acid (30 μL, 0.34 mmol, 37.4 equiv.) at –78 °C. After stirring 20 min at –78 °C, the reaction mixture was allowed to warm to –40 °C and was further stirred at –40 °C for 15 min. Saturated $NaHCO_3$ solution (2 mL) was added at –40 °C and the mixture was extracted four times with EtOAc. The combined organic phases were dried over anhydrous Na_2SO_4, filtered and concentrated under reduced pressure. The residue was purified by preparative thin layer chromatography ($CHCl_3$:MeOH:aq. NH_4OH = 88:11:1) to give heilonine **1** (3.2 mg, 86%) as a white solid.

Rf = 0.5 ($CHCl_3$: MeOH : aq. NH_4OH = 88 : 11 : 1; UV)

$[\alpha]_D^{23}$ = +71.5° (c = 0.200 g / 100 mL in MeOH)

IR (thin film) 3397, 2925, 2851, 2758, 1654, 1637, 1590, 1458, 1440, 1360, 1299, 1223, 1032, 809, 751, 687 cm^{-1}

^{1}H NMR (800 MHz, CD_3OD) δ 7.05 (d, *J* = 7.8 Hz, 1H), 6.96 (d, *J* = 7.7 Hz, 1H), 3.94 (ddd, *J* = 2.8, 2.8, 2.8 Hz, 1H), 3.70 (d, *J* = 14.8 Hz, 1H), 3.61 (dddd, *J* = 10.7, 10.7, 4.9, 4.9 Hz, 1H), 3.24 (d, *J* = 14.8 Hz, 1H), 3.23 – 3.17 (m, 1H), 3.01 (ddd, *J* = 11.4, 2.7, 2.7 Hz, 1H), 2.70 (qd, *J* = 7.2, 7.2 Hz, 1H), 2.58 (dd, *J* = 14.3, 6.7 Hz, 1H), 2.47 (dd, *J* = 13.4, 12.9 Hz, 1H), 2.41 (ddd, *J* = 13.1, 3.2, 3.2 Hz, 1H), 2.17 (dddd, *J* = 13.4, 3.3, 3.3, 3.3 Hz, 1H), 1.88 – 1.70 (m, 6H), 1.70–1.65 (m, 1H), 1.61 (ddd, *J* = 12.8, 3.5, 3.5 Hz, 1H), 1.59 – 1.49 (m, 3H), 1.35 – 1.28 (m, 1H), 1.27 (d, *J* = 6.8 Hz, 3H), 1.25 – 1.18 (m, 2H) 1.16 (s, 3H), 1.05 (dddd, *J* = 13.2, 3.7, 3.7, 3.7 Hz, 1H), 0.93 (d, *J* = 6.5 Hz, 3H) ppm

^{13}C NMR (200 MHz, CD_3OD) δ 145.3, 140.5, 137.5, 130.4, 126.2, 121.4, 73.4, 72.7, 67.2, 65.0, 62.8, 57.1, 49.9, 40.1 (2 carbons overlapped), 40.0, 38.5, 36.9, 35.7, 34.0, 32.7, 31.9, 31.8, 29.6, 20.4, 19.9, 15.8 ppm

HRMS (ESI) *m/z* $[M+H]^+$ calcd for $C_{27}H_{40}O_2N$: 410.3054; found: 410.3064

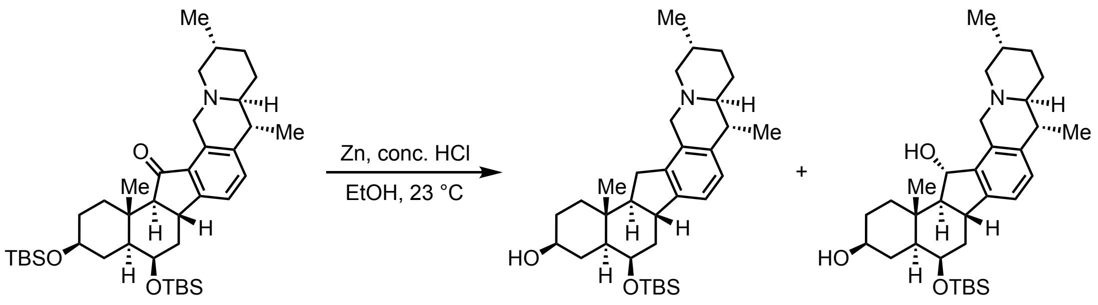

45 **S5** **S6**

25% 52%

To a suspension of **45** (9.8 mg, 15 μmol) and Zn powder (120 mg, 1.84 mmol, 122 equiv.) in EtOH (1.5 mL), concentrated HCl (0.7 mL) was slowly added over 10 min. After stirring for 10 min at room temperature, Zn powder (60 mg, 0.92 mmol, 61 equiv.) was added to the mixture per 30 min. After adding 4 times of Zn powder (4 x 60 mg over 2 h), the mixture was further stirred at room temperature for 1 h. Then the mixture was filtered, the resulting solution was brought to pH 12 with aqueous NH_3 (30%). The mixture was extracted 3 times with EtOAc. The combined organic layers were washed twice with brine and dried over anhydrous Na_2SO_4. The solvent was evaporated and the residue was purified by preparative thin layer chromatography (3%Et_3N in hexane:EtOAc = 2:1) to give alcohol **S5** (2.0 mg, 25%) as a white solid and **S6** (4.2 mg, 52%) as a white solid. Note: when **S6** was resubmitted to the same reaction conditions, **S5** was not obtained.

S5

$\boldsymbol{R_f}$ = 0.6 (3%Et_3N in hexane:EtOAc = 2:1; UV)

$[\alpha]_D^{22}$ = +25.4° (c = 0.110 g / 100 mL in CH_2Cl_2)

IR (thin film) 3333, 2925, 2853, 1460, 1440, 1258, 1047, 959, 807 cm^{-1}

^{1}H NMR (600 MHz, $CDCl_3$) δ 7.05 (d, J = 7.8 Hz, 1H), 6.94 (d, J = 7.7 Hz, 1H), 3.94 (d, J = 2.7 Hz, 1H), 3.74 – 3.63 (m, 2H), 3.25 – 3.15 (m, 2H), 3.04 – 2.97 (m, 1H), 2.69 (dt, J = 6.8, 6.8 Hz, 1H), 2.54 (dd, J = 14.3, 6.8 Hz, 1H), 2.45 (dd, J = 13.1, 13.1 Hz, 1H), 2.29 (ddd, J = 12.9, 3.2, 3.2 Hz, 1H), 2.15 – 2.07 (m, 1H), 1.86 – 1.68 (m, 6H), 1.67 – 1.42 (m, 5H), 1.36 – 1.23 (m, 4H), 1.19 – 1.08 (m, 5H), 1.04 – 0.81 (m, 13H), 0.08 (s, 3H), 0.06 (s, 3H) ppm

^{13}C NMR (150 MHz, $CDCl_3$) δ 143.8, 139.4, 136.7, 130.2, 124.9, 119.9, 73.1, 72.0, 65.5, 64.4, 61.2, 56.3, 48.9, 39.1, 39.0, 38.6, 37.8, 35.7, 35.5, 33.1, 31.9, 31.3, 30.8, 28.7, 25.8, 19.7, 19.5, 18.0, 15.6, -4.4, -4.9 ppm

HRMS (ESI) m/z $[M+Na]^+$ calcd for $C_{33}H_{54}O_2NSi$: 524.3918; found: 524.3921

S6

$\boldsymbol{R_f}$ = 0.2 (3%Et_3N in hexane:EtOAc = 2:1; UV)

[α]D22 = +31.1° (c = 0.180 g / 100 mL in CH_2Cl_2)

IR (thin film) 3349, 2949, 2926, 2855, 1460, 1361, 1254, 1044, 835 cm^{-1}

^{1}H NMR (800 MHz, $CDCl_3$) δ 7.10 (d, J = 7.8 Hz, 1H), 6.94 (d, J = 7.7 Hz, 1H), 5.15 (d, J = 9.0 Hz, 1H), 3.95 (d, J = 15.5 Hz, 1H), 3.92 (d, J = 2.7 Hz, 1H), 3.66 (dddd, J = 10.8, 10.8, 4.9, 4.9 Hz, 1H), 3.38 (d, J = 15.5 Hz, 1H), 3.07 (ddd, J = 12.0, 12.0, 3.3 Hz, 1H), 3.03 (br d, J = 8.8 Hz, 1H), 2.70 (dt, J = 7.2, 7.2 Hz, 1H), 2.30 (ddd, J = 12.8, 3.2, 3.2 Hz, 1H), 2.11 – 2.06 (m, 1H), 1.92 – 1.83 (m, 2H), 1.82 – 1.68 (m, 5H), 1.67 – 1.42 (m, 4H), 1.36 – 1.28 (m, 2H), 1.26 (d, J = 6.9 Hz, 3H), 1.22 – 1.12 (m, 4H), 1.03 – 0.95 (m, 1H), 0.90 (d, J = 5.8 Hz, 3H), 0.88 (s, 9H), 0.07 (s, 3H), 0.06 (s, 3H) ppm

^{13}C NMR (200 MHz, $CDCl_3$) δ 141.7, 140.4, 138.1, 132.0, 127.2, 120.0, 75.5, 72.9, 71.8, 70.7, 66.0, 64.4, 56.2, 48.9, 39.5, 39.4, 38.0, 36.2, 35.4, 35.3, 33.1, 32.3, 31.2, 30.9, 25.8, 20.5, 19.7, 17.9, 16.2, -4.5, -4.9 ppm

HRMS (ESI) *m/z* $[M+Na]^+$ calcd for $C_{33}H_{54}O_3NSi$: 540.3868; found: 540.3875

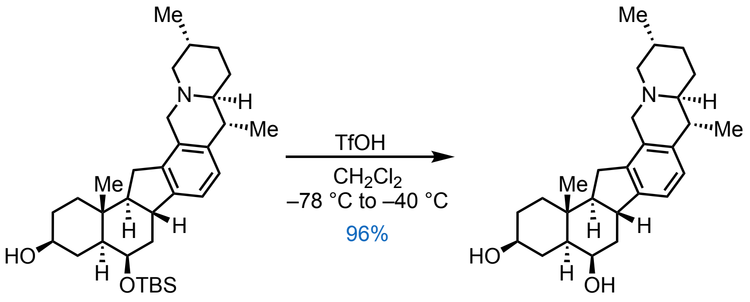

S5 (+)-heilonine (**1**)

To a solution of **S5** (2.0 mg, 3.8 μmol, 1.0 equiv.) in CH_2Cl_2 (0.4 mL) was added dropwise trifluoromethanesulfonic acid (10 μL, 0.11 mmol, 29 equiv.) at –78 °C. After stirring 20 min at –78 °C, the reaction mixture was allowed to warm to –40 °C and was further stirred at –40 °C for 15 min. Saturated $NaHCO_3$ solution (2 mL) was added at –40 °C and the mixture was warmed to room temperature and extracted 4 times with EtOAc. The combined organic phases were dried over anhydrous Na_2SO_4, filtered and concentrated under reduced pressure. The residue was purified by preparative thin layer chromatography ($CHCl_3$:MeOH:aq. NH_4OH = 88:11:1) to give heilonine (1.5 mg, 96%) as a white solid.

For ^{1}H NMR and ^{13}C NMR comparison between our synthetic sample and Rawal's synthetic sample[7]:

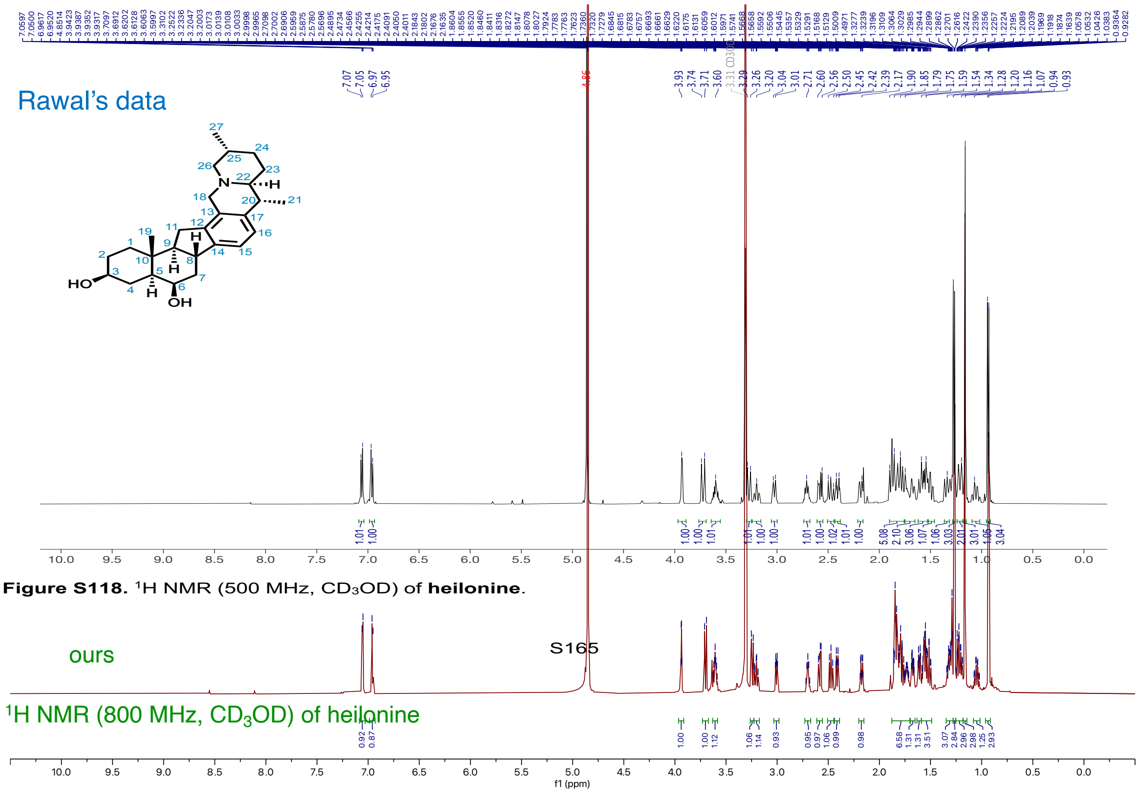

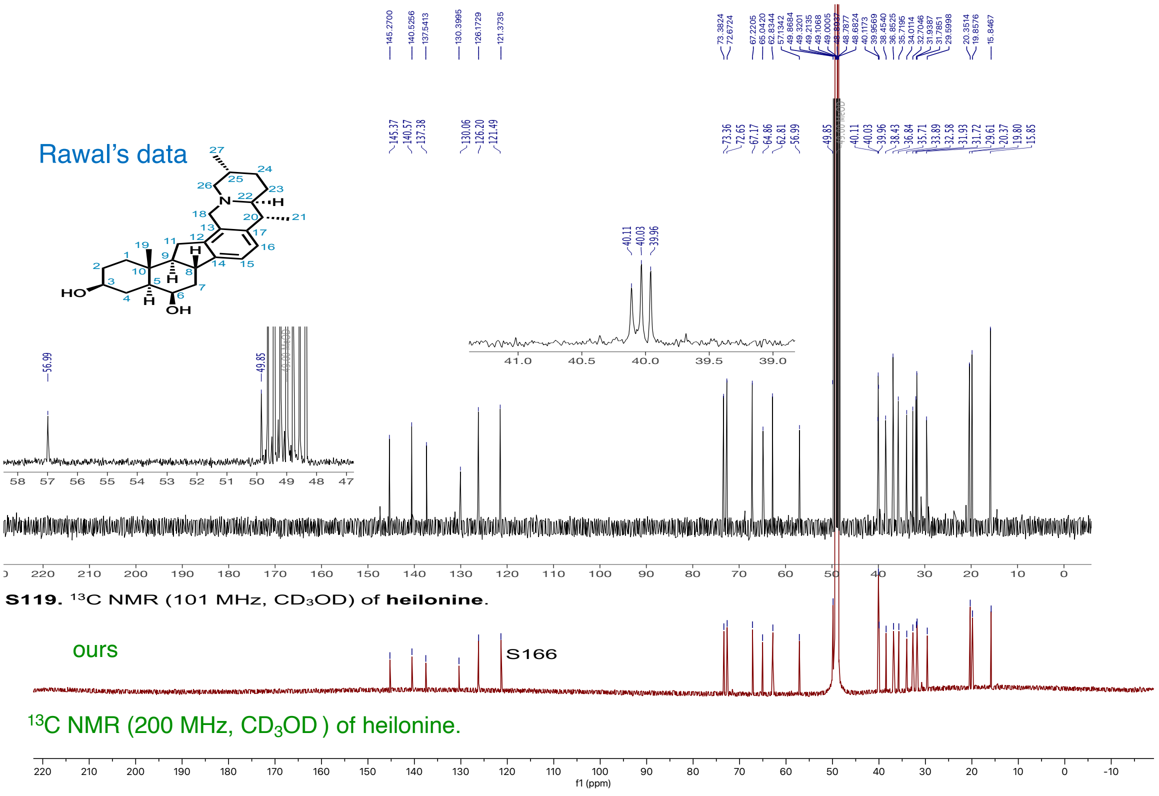

Heilonine diacetate[8] was synthesized to further confirm our structural assignment.

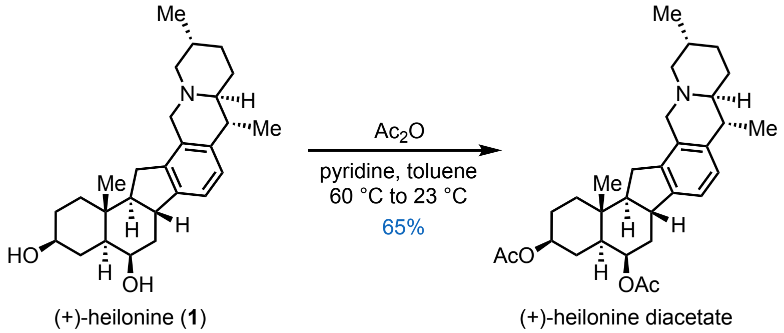

To a solution of heilonine **1** (2.3 mg, 5.6 μmol, 1.0 equiv.) in acetic anhydride (0.5 mL) was added pyridine (0.5 mL) at room temperature and the mixture was stirred at 60 °C for 6 h. It was then cooled to room temperature and was further stirred for 18 h at room temperature. Volatiles were evaporated to give a residue. The residue was purified by preparative thin layer chromatography (3% Et_3N in hexane:EtOAc = 15:2) to give heilonine diacetate (1.8 mg, 65%) as a white solid.

R_f = 0.35 (3% Et_3N in hexane:EtOAc = 15:2; UV).

$[\alpha]_D^{23}$= +32.3° (c = 0.130 g / 100 mL in $CHCl_3$)

IR (thin film) 2926, 2853, 1734, 1458, 1439, 1376, 1363, 1239, 1139, 1097, 1077, 1024, 955, 829, 809, 752, 667, 607 cm^{-1}

^{1}H NMR (600 MHz, $CDCl_3$) δ 7.06 (d, J = 7.8 Hz, 1H), 6.93 (d, J = 7.7 Hz, 1H), 5.10 (dt, 2.9, 2.9 Hz, 1H), 4.78 (sept, J = 5.0 Hz, 1H), 3.70 (d, J = 15.1 Hz, 1H), 3.23 (d, J = 15.0 Hz, 1H), 3.09 (td, J = 12.2, 3.0 Hz, 1H), 3.01 (br s, 1H), 2.71 (br s, 1H), 2.58 (dd, J = 14.3, 6.7 Hz, 1H), 2.50 – 2.43 (m, 2H), 2.16 – 2.09 (m, 1H), 2.07 (s, 3H), 2.04 (s, 3H), 1.93 – 1.70 (m, 6H), 1.69 – 1.49 (m, 5H), 1.42 (dt, J = 13.0, 2.8 Hz, 1H), 1.35 – 1.19 (m, 2H), 1.27 (d, J = 6.8 Hz, 3H), 1.15 (s, 3H), 1.05 – 0.96 (m, 1H), 0.91 (d, J = 5.4 Hz, 3H) ppm

^{13}C NMR (150 MHz, $CDCl_3$) δ 170.5, 170.5, 142.7, 139.0, 137.1, 130.1, 125.2, 120.1, 74.0, 73.4, 65.6, 64.4, 60.5, 56.2, 47.1, 39.0 (2 carbons overlapped), 38.4, 35.7, 34.1, 33.0, 32.0, 30.8, 30.3, 28.6, 27.0, 21.4, 21.3, 19.7 (2 carbons overlapped), 14.9 ppm

HRMS (ESI) m/z $[M+H]^+$ calcd for $C_{31}H_{44}O_4N$: 494.3265; found: 494.3265.

^{1}H NMR spectral data comparison of synthetic heilonine diacetate and natural heilonine diacetate.[8]

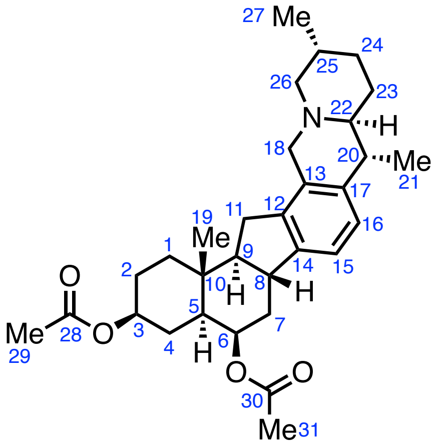

Heilonine diacetate

No.	Reported δ_H (270 MHz, $CDCl_3$) [8]	Synthetic δ_H (600 MHz, $CDCl_3$)	$\Delta\delta$ (ppm)
1	1.23 (m, 1H)<br>1.65 (m, 1H)	1.25 (m, 1H)<br>1.66 (m, 1H)	0.02<br>0.01
2	1.65 (m, 1H)<br>1.87 (m, 1H)	1.64 (m, 1H)<br>1.88 (m, 1H)	-0.01<br>0.01
3	4.78 (sept, *J* = 5 Hz, 1H)	4.78 (sept, *J* = 5.0 Hz, 1H)	0.00
4	1.65 (m, 1H)<br>1.76 (m, 1H)	1.64 (m, 1H)<br>1.75 (m, 1H)	-0.01<br>-0.01
5	1.41 (m, 1H)	1.42 (dt, *J* = 13.0, 2.8 Hz, 1H)	0.01
6	5.10 (m, $W_{1/2}$ = 8 Hz, 1H)	5.10 (dt, 2.9, 2.9 Hz, 1H)	0.00
7	1.57 (dt, *J* = 13.8, 3.0 Hz, 1H)<br>2.47 (dd, *J* = 14.0, 3.5 Hz, 1H)	1.57 (td, *J* = 13.6, 3.1 Hz, 1H)<br>2.47 (m, 1H)	0.00<br>0.00
8	3.12 (dt, *J* = 12.2, 7 Hz, 1H)	3.09 (td, *J* = 12.2, 3.0 Hz, 1H)	-0.03
9	1.62 (m, 1H)	1.64 (m, 1H)	0.02
11	2.50 (br t, *J* = 13.6 Hz, 1H)<br>2.57 (dd, *J* = 14.5, 7.1 Hz, 1H)	2.47 (m, 1H)<br>2.58 (dd, *J* = 14.3, 6.7 Hz, 1H)	-0.03<br>0.01
15	6.96 (d, *J* = 7.8 Hz, 1H)	6.93 (d, *J* = 7.7 Hz, 1H)	-0.03
16	7.08 (d, *J* = 7.8 Hz, 1H)	7.06 (d, *J* = 7.8 Hz, 1H)	-0.02
18	3.32(d, *J* = 15.0 Hz,1H)<br>3.92(d, *J* = 15.0 Hz,1H)	3.23 (d, *J* = 15.0 Hz, 1H)<br>3.70 (d, *J* = 15.1 Hz, 1H)	-0.09<br>-0.22

19	1.16 (s, 3H)	1.15 (s, 3H)	-0.01
20	2.84 (quintet, *J* = 7 Hz, 1H)	2.71 (br s, 1H)	-0.13
21	1.29 (d, *J* = 6.9 Hz, 3H)	1.27 (d, *J* = 6.8 Hz, 3H)	-0.02
22	1.99 (m, 1H)	1.81 (m, 1H)	-0.18
23	1.47 (m, 1H),<br>2.15 (br dd, *J* = 13.7, 2.7 Hz, 1H)	1.33 (m, 1H)<br>2.12 (m, 1H)	-0.14<br>-0.03
24	1.04 (qd, *J* = 11.8, 4 Hz, 1H)<br>1.85 (m, 1H)	1.00 (m, 1H)<br>1.83 (m, 1H)	-0.04<br>-0.02
25	1.88 (m, 1H)	1.81 (m, 1H)	-0.07
26	1.89 (m, 1H)<br>3.20 (br d, *J* = 10 Hz, 1H)	1.82 (m, 1H)<br>3.01 (br s, 1H)	-0.07<br>-0.19
27	0.93 (d, *J* = 5.4 Hz, 3H)	0.91 (d, *J* = 5.4 Hz, 3H)	-0.02
acetate	2.04 (s, 3H)<br>2.06 (s, 3H)	2.04 (s, 3H)<br>2.07 (s, 3H)	0.00<br>0.01

^{13}C NMR spectral data comparison of synthetic heilonine diacetate and natural heilonine diacetate.[8]

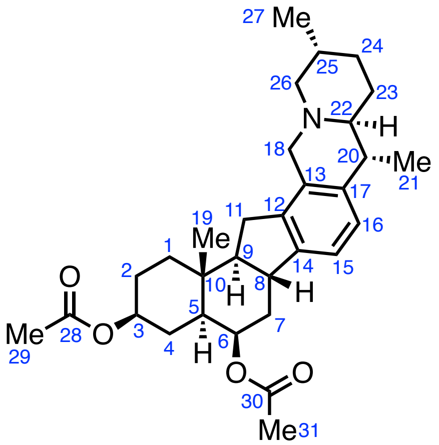

Heilonine diacetate

No.	Reported δ_C (100 MHz, $CDCl_3$) [8]	Synthetic δ_C (150 MHz, $CDCl_3$)	Δδ (ppm)
1	38.4	38.4	0.0
2	27.1	27.0	-0.1
3	73.4	73.4	0.0

4	30.4	30.3	-0.1
5	47.1	47.1	0.0
6	74.0	74.0	0.0
7	34.2	34.1	-0.1
8	39.0	39.0	0.0
9	60.5	60.5	0.0
10	35.7	35.7	0.0
11	28.6	28.6	0.0
12	142.8	142.7	-0.1
13	136.7	137.1	0.4
14	139.0	139.0	0.0
15	120.2	120.1	-0.1
16	125.2	125.2	0.0
17	129.5	130.1	0.6
18	55.8	56.2	0.4
19	14.9	14.9	0.0
20	38.7	39.0	0.3
21	19.49	19.7	0.2
22	65.5	65.6	0.1
23	31.5	32.0	0.5
24	32.9	33.0	0.1
25	30.5	30.8	0.3
26	64.0	64.4	0.4
27	19.57	19.7	0.1
acetate	170.5<br>170.5<br>21.27<br>21.34	170.5<br>170.5<br>21.3<br>21.4	0.0<br>0.0<br>0.0<br>0.1

For ^{1}H NMR and ^{13}C NMR comparison between our synthetic heilonine diacetate and Rawal's synthetic heilonine diacetate[7]:

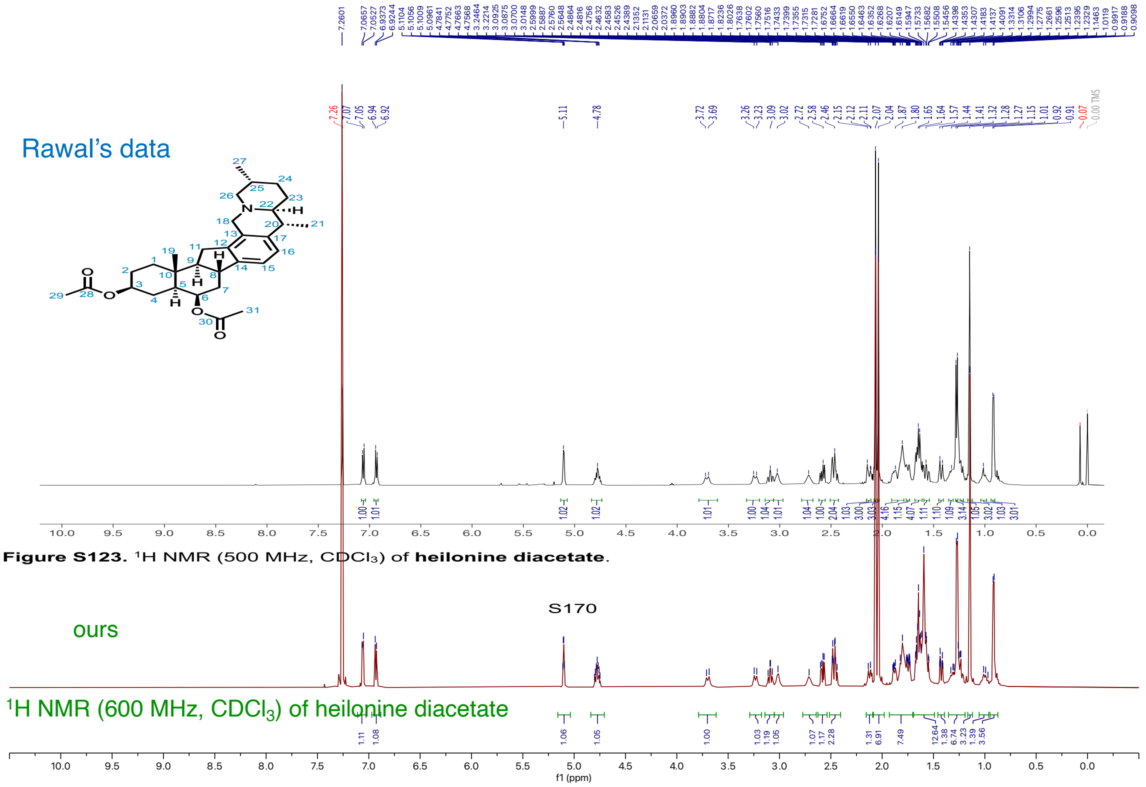

Figure S123. ^1H NMR (500 MHz, $CDCl_3$) of **heilonine diacetate**.

^1H NMR (600 MHz, $CDCl_3$) of heilonine diacetate

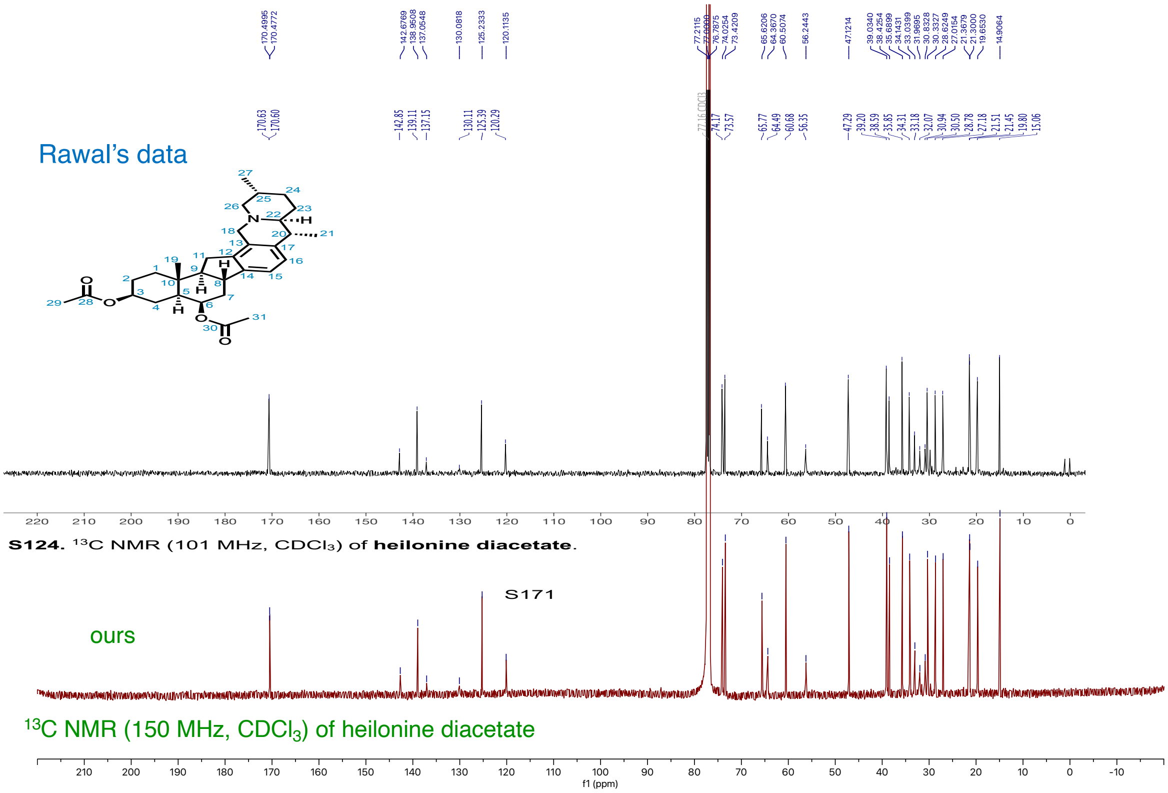

S124. ^{13}C NMR (101 MHz, $CDCl_3$) of **heilonine diacetate**.

^{13}C NMR (150 MHz, $CDCl_3$) of heilonine diacetate

Part 2. X-ray structure and analysis data

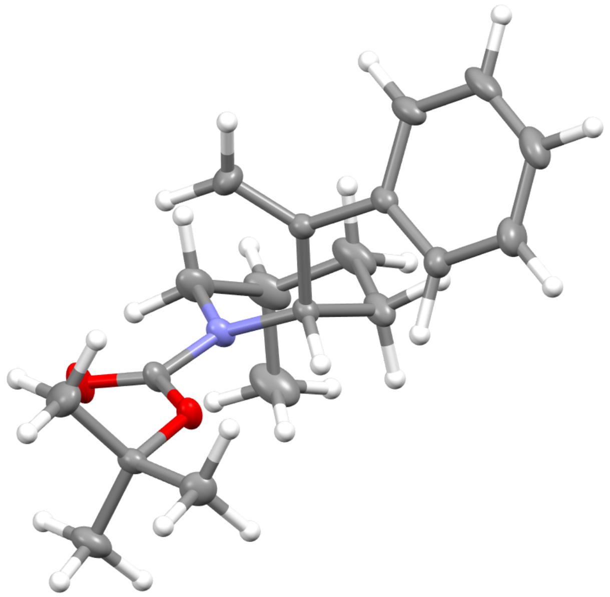

Figure S1. Single-crystal X-ray diffraction analysis of compound **24** (CCDC 2306389; 50% probability of the thermal ellipsoids).

Crystal Data for Compound **24** (*M* =301.432 g/mol): orthorhombic, space group $P2_12_12_1$ (no. 19), *a* = 5.95738(19) Å, *b* = 14.5370(4) Å, *c* = 20.4632(5) Å, *V* = 1772.16(9) Å^3, *Z* = 4, *T* = 105(7) K, μ(Cu Kα) = 0.566 mm^{-1}, *Dcalc* = 1.130 g/cm^3, 10312 reflections measured (7.46° ≤ 2Θ ≤ 153.26°), 3305 unique (R_{int} = 0.0463, R_{sigma} = 0.0399) which were used in all calculations. The final R_1 was 0.0291 (I>=2u(I)) and wR_2 was 0.0669 (all data).

Identification code	YJ01319-VRF
Empirical formula	$C_{19}H_{27}NO_2$
Formula weight	301.432
Temperature/K	105(7)
Crystal system	orthorhombic
Space group	$P2_12_12_1$
a/Å	5.95738(19)
b/Å	14.5370(4)
c/Å	20.4632(5)
α/°	90

β/°	90
γ/°	90
Volume/Å^3	1772.16(9)
Z	4
ρ_{calc}g/cm^3	1.130
μ/mm^{-1}	0.566
F(000)	657.9
Crystal size/mm^3	0.37 × 0.17 × 0.1
Radiation	Cu Kα (λ = 1.54184)
2Θ range for data collection/°	7.46 to 153.26
Index ranges	$-7 \leq h \leq 6$, $-18 \leq k \leq 17$, $-25 \leq l \leq 18$
Reflections collected	10312
Independent reflections	3305 [R_{int} = 0.0463, R_{sigma} = 0.0399]
Data/restraints/parameters	3305/504/374
Goodness-of-fit on F^2	1.049
Final R indexes [I>=2σ (I)]	R_1 = 0.0291, wR_2 = 0.0657
Final R indexes [all data]	R_1 = 0.0313, wR_2 = 0.0669
Largest diff. peak/hole / e Å^{-3}	0.13/-0.11
Flack parameter	-0.00(13)

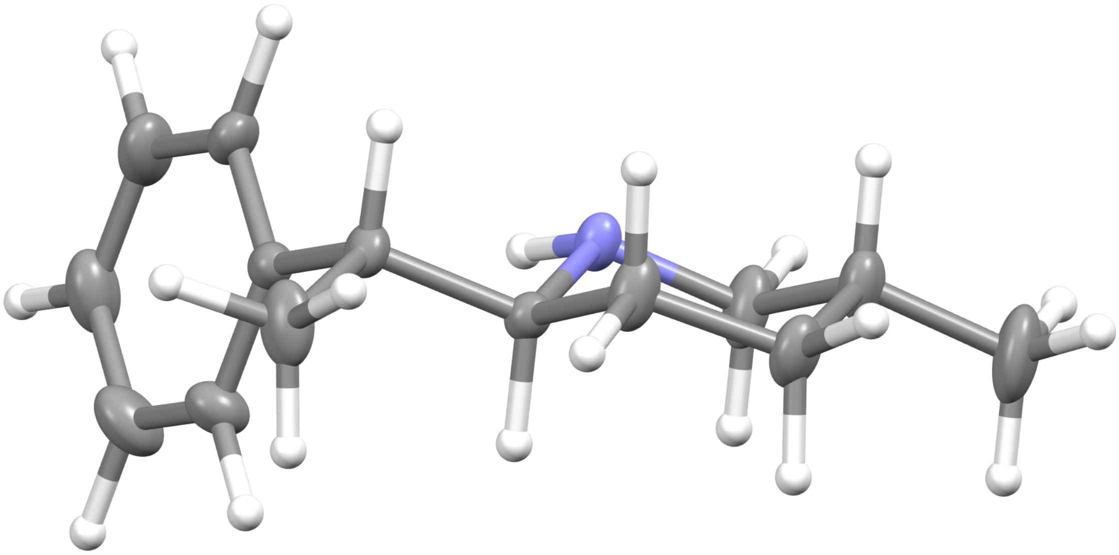

Figure S2. Single-crystal X-ray diffraction analysis of compound **20** (CCDC 2306390; 50% probability of the thermal ellipsoids).

Crystal Data for Compound **20** (M =203.330 g/mol): monoclinic, space group $P2_1$ (no. 4), a = 20.8246(8) Å, b = 10.8685(4) Å, c = 5.5737(2) Å, β = 90.031(4)°, V = 1261.50(8) Å^3, Z = 4, T = 99.9(2) K, μ(Cu Kα) = 0.458 mm^{-1}, $Dcalc$ = 1.071 g/cm^3, 15097 reflections measured (9.18° ≤ 2Θ ≤ 153.38°), 4711 unique (R_{int} = 0.0414, R_{sigma} = 0.0365) which were used in all calculations. The final R_1 was 0.0233 (I>=2u(I)) and wR_2 was 0.0493 (all data).

Identification code	YJ01322_VRF
Empirical formula	$C_{14}H_{21}N$
Formula weight	203.330
Temperature/K	99.9(2)
Crystal system	monoclinic
Space group	$P2_1$
a/Å	20.8246(8)
b/Å	10.8685(4)
c/Å	5.5737(2)
α/°	90
β/°	90.031(4)
γ/°	90
Volume/Å^3	1261.50(8)

Z	4
ρ_{calc}g/cm^3	1.071
μ/mm^{-1}	0.458
F(000)	449.1
Crystal size/mm^3	0.43 × 0.22 × 0.13
Radiation	Cu Kα (λ = 1.54184)
2Θ range for data collection/°	9.18 to 153.38
Index ranges	$-24 \leq h \leq 25, -13 \leq k \leq 12, -6 \leq l \leq 5$
Reflections collected	15097
Independent reflections	4711 [R_{int} = 0.0414, R_{sigma} = 0.0365]
Data/restraints/parameters	4711/1061/570
Goodness-of-fit on F^2	1.097
Final R indexes [I>=2σ (I)]	R_1 = 0.0233, wR_2 = 0.0488
Final R indexes [all data]	R_1 = 0.0245, wR_2 = 0.0493
Largest diff. peak/hole / e Å^{-3}	0.08/-0.08
Flack parameter	-0.3(2)

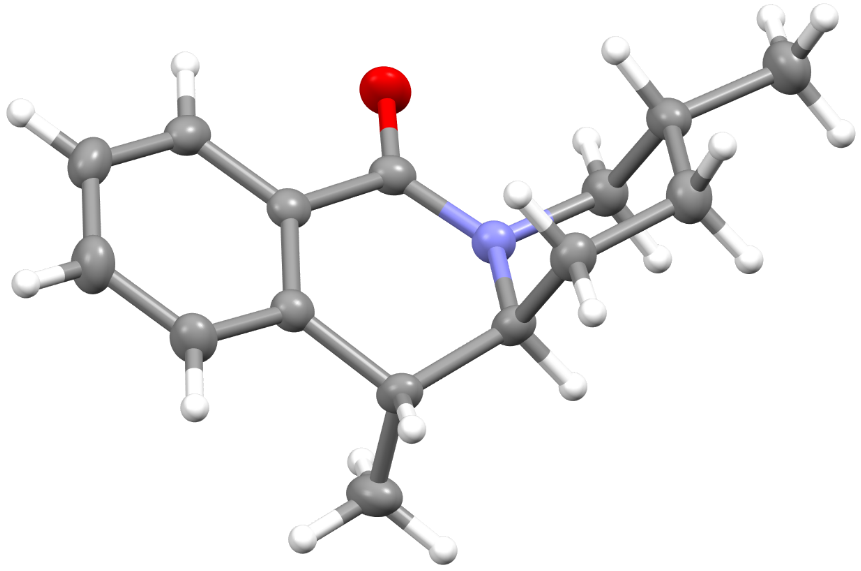

Figure S3. Single-crystal X-ray diffraction analysis of compound **19** (CCDC 2306384; 50% probability of the thermal ellipsoids)

Crystal Data for Compound **19** (M =229.31 g/mol): hexagonal, space group $P6_1$ (no. 169), a = 17.8803(5) Å, c = 7.3138(2) Å, V = 2024.99(13) Å^3, Z = 6, T = 150(2) K, μ(CuKα) = 0.545 mm^{-1}, $Dcalc$ = 1.128 g/cm^3, 12873 reflections measured (5.708° ≤ 2Θ ≤ 159.448°), 2629 unique (R_{int} = 0.0450, R_{sigma} = 0.0310) which were used in all calculations. The final R_1 was 0.0305 (I > 2σ(I)) and wR_2 was 0.0728 (all data).

Identification code	yj01188_0m_sq_complete
Empirical formula	$C_{15}H_{19}NO$
Formula weight	229.31
Temperature/K	150(2)
Crystal system	hexagonal
Space group	$P6_1$
a/Å	17.8803(5)
b/Å	17.8803(5)
c/Å	7.3138(2)
α/°	90
β/°	90
γ/°	120

Volume/Å^3	2024.99(13)
Z	6
ρ_{calc}g/cm^3	1.128
μ/mm^{-1}	0.545
F(000)	744.0
Crystal size/mm^3	0.330 × 0.120 × 0.090
Radiation	CuKα (λ = 1.54178)
2Θ range for data collection/°	5.708 to 159.448
Index ranges	-20 ≤ h ≤ 19, -20 ≤ k ≤ 22, -8 ≤ l ≤ 8
Reflections collected	12873
Independent reflections	2629 [R_{int} = 0.0450, R_{sigma} = 0.0310]
Data/restraints/parameters	2629/1/157
Goodness-of-fit on F^2	1.055
Final R indexes [I>=2σ (I)]	R_1 = 0.0305, wR_2 = 0.0707
Final R indexes [all data]	R_1 = 0.0337, wR_2 = 0.0728
Largest diff. peak/hole / e Å^{-3}	0.14/-0.15
Flack parameter	-0.08(13)

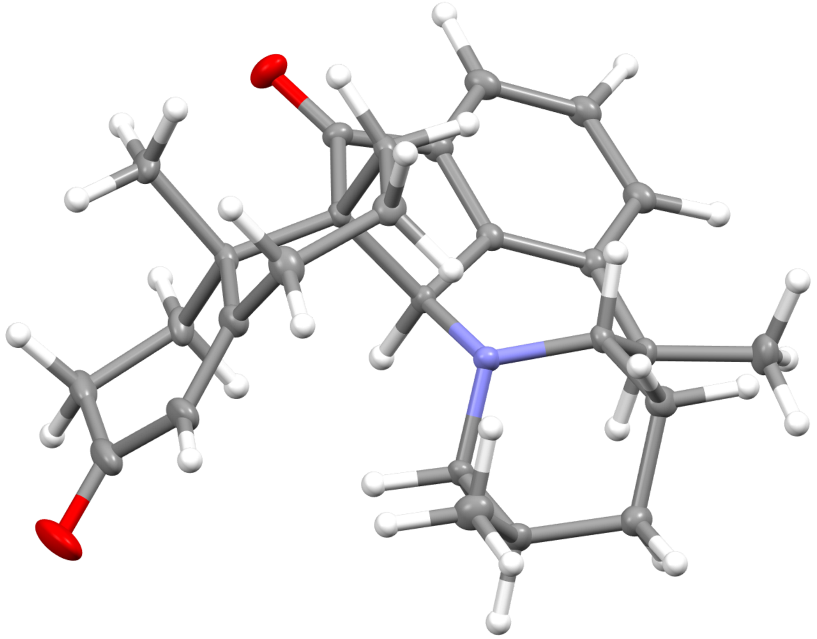

Figure S4. Single-crystal X-ray diffraction analysis of compound **30** (CCDC 2309636; 50% probability of the thermal ellipsoids).

Crystal Data for Compound **30** (M =403.568 g/mol): tetragonal, space group P4$_3$ (no. 78), a = 10.11031(17) Å, c = 41.5310(14) Å, V = 4245.24(17) Å^3, Z = 8, T = 101(2) K, μ(Cu Kα) = 0.609 mm^{-1}, $Dcalc$ = 1.263 g/cm^3, 28389 reflections measured (8.74° ≤ 2Θ ≤ 135.12°), 7537 unique (R_{int} = 0.0691, R_{sigma} = 0.0559) which were used in all calculations. The final R_1 was 0.0316 (I>=2u(I)) and wR_2 was 0.0604 (all data).

Identification code	YJ01272_SP1-finalcif_VRF
Empirical formula	$C_{27}H_{33}NO_2$
Formula weight	403.568
Temperature/K	101(2)
Crystal system	tetragonal
Space group	P4$_3$
a/Å	10.11031(17)
b/Å	10.11031(17)
c/Å	41.5310(14)
α/°	90
β/°	90
γ/°	90
Volume/Å^3	4245.24(17)
Z	8
ρ_{calc}g/cm^3	1.263
μ/mm^{-1}	0.609
F(000)	1749.2
Crystal size/mm^3	0.54 × 0.519 × 0.047
Radiation	Cu Kα (λ = 1.54178)
2Θ range for data collection/°	8.74 to 135.12
Index ranges	-12 ≤ h ≤ 11, -8 ≤ k ≤ 11, -49 ≤ l ≤ 49
Reflections collected	28389
Independent reflections	7537 [R_{int} = 0.0691, R_{sigma} = 0.0559]
Data/restraints/parameters	7537/1452/933
Goodness-of-fit on F^2	1.066

Final R indexes [I>=2σ (I)]	R_1 = 0.0316, wR_2 = 0.0599
Final R indexes [all data]	R_1 = 0.0329, wR_2 = 0.0604
Largest diff. peak/hole / e $Å^{-3}$	0.12/-0.11

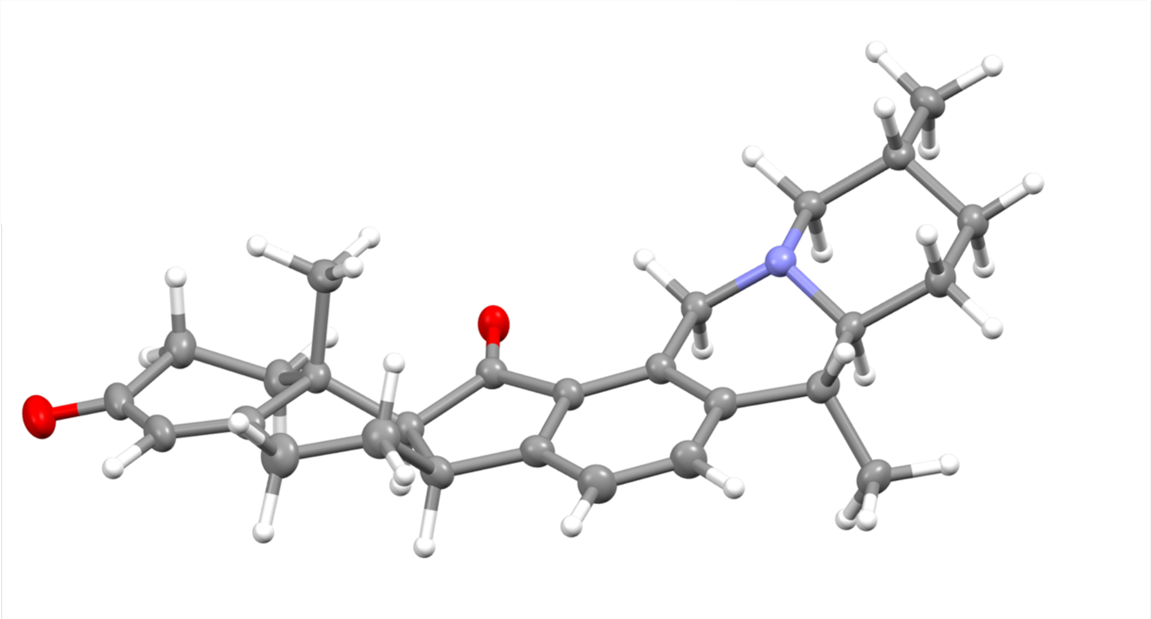

Figure S5. Single-crystal X-ray diffraction analysis of compound **33** (CCDC 2306386; 50% probability of the thermal ellipsoids).

Crystal Data for Compound **33** (M =403.568 g/mol): orthorhombic, space group $P2_12_12_1$ (no. 19), a = 7.9628(1) Å, b = 16.7701(2) Å, c = 47.9459(6) Å, V = 6402.55(14) Å^3, Z = 12, T = 100(2) K, μ(Cu Kα) = 0.605 mm^{-1}, $Dcalc$ = 1.256 g/cm^3, 39007 reflections measured (5.58° ≤ 2Θ ≤ 146.24°), 11982 unique (R_{int} = 0.0721, R_{sigma} = 0.0695) which were used in all calculations. The final R_1 was 0.0380 (I>=2u(I)) and wR_2 was 0.0743 (all data).

Identification code	YJ01272-sp2-final-wVRF
Empirical formula	$C_{27}H_{33}NO_2$
Formula weight	403.568
Temperature/K	100(2)
Crystal system	orthorhombic
Space group	$P2_12_12_1$
a/Å	7.9628(1)
b/Å	16.7701(2)
c/Å	47.9459(6)
α/°	90
β/°	90
γ/°	90

Volume/Å^3	6402.55(14)
Z	12
ρ_{calc}g/cm^3	1.256
μ/mm^{-1}	0.605
F(000)	2623.9
Crystal size/mm^3	0.14 × 0.11 × 0.04
Radiation	Cu Kα (λ = 1.54178)
2Θ range for data collection/°	5.58 to 146.24
Index ranges	$-9 \leq h \leq 9$, $-17 \leq k \leq 20$, $-48 \leq l \leq 58$
Reflections collected	39007
Independent reflections	11982 [R_{int} = 0.0721, R_{sigma} = 0.0695]
Data/restraints/parameters	11982/31/1187
Goodness-of-fit on F^2	1.018
Final R indexes [I>=2σ (I)]	R_1 = 0.0380, wR_2 = 0.0700
Final R indexes [all data]	R_1 = 0.0508, wR_2 = 0.0743
Largest diff. peak/hole / e Å^{-3}	0.18/-0.17
Flack parameter	0.13(14)

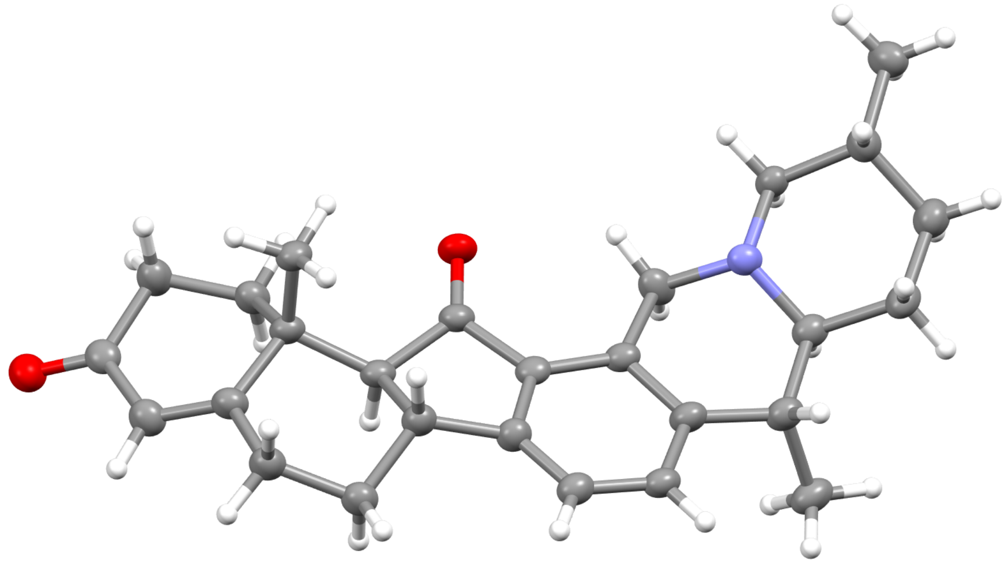

Figure S6. Single-crystal X-ray diffraction analysis of compound **35** (CCDC 2310768; 50% probability of the thermal ellipsoids).

Crystal Data for Compound **35** (M =403.54 g/mol): monoclinic, space group P2 (no. 3), a = 10.0421(5) Å, b = 7.8368(3) Å, c = 28.1303(13) Å, β = 100.259(5)°, V = 2178.41(17) $Å^3$, Z = 4, T = 99.9(3) K, μ(Cu Kα) = 0.593 mm^{-1}, $Dcalc$ = 1.230 g/cm^3, 23937 reflections measured (9.586° ≤ 2Θ ≤ 130.168°), 6916 unique (R_{int} = 0.0469, R_{sigma} = 0.0579) which were used in all calculations. The final R_1 was 0.1077 (I > 2σ(I)) and wR_2 was 0.2612 (all data).

Identification code	YJ01295-sp1_VRF
Empirical formula	$C_{27}H_{33}NO_2$
Formula weight	403.54
Temperature/K	99.9(3)
Crystal system	monoclinic
Space group	P2
a/Å	10.0421(5)
b/Å	7.8368(3)
c/Å	28.1303(13)
α/°	90
β/°	100.259(5)

γ/°	90
Volume/Å^3	2178.41(17)
Z	4
ρ_{calc}g/cm^3	1.230
μ/mm^{-1}	0.593
F(000)	872.0
Crystal size/mm^3	0.102 × 0.071 × 0.032
Radiation	Cu Kα (λ = 1.54184)
2Θ range for data collection/°	9.586 to 130.168
Index ranges	-11 ≤ h ≤ 11, -9 ≤ k ≤ 9, -33 ≤ l ≤ 27
Reflections collected	23937
Independent reflections	6916 [R_{int} = 0.0469, R_{sigma} = 0.0579]
Data/restraints/parameters	6916/595/542
Goodness-of-fit on F^2	1.115
Final R indexes [I>=2σ (I)]	R_1 = 0.1077, wR_2 = 0.2444
Final R indexes [all data]	R_1 = 0.1206, wR_2 = 0.2612
Largest diff. peak/hole / e Å^{-3}	1.35/-0.37
Flack parameter	-0.20(19)

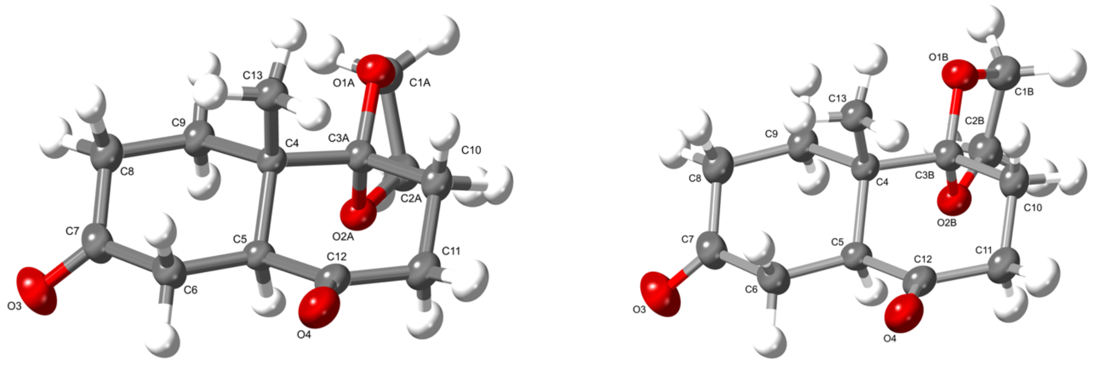

Figure S7. Single-crystal X-ray diffraction analysis of compound **38** (CCDC 2306391; 50% probability of the thermal ellipsoids). The dioxolane ring within the structure exhibits disorder and can be readily analyzed by interpreting its conformational pucker. The dioxolane ring possesses approximate C2v symmetry. The five-membered ring includes two oxygen atoms and a spiro carbon, along with an additional two carbons. The two dioxolane oxygen atoms, the spiro carbon, and one carbon from the same 5-ring are approximately coplanar (and can be defined as the horizontal reflection plane). At this intersection, the spiro carbon connects with two carbons from the 6-ring, forming an almost vertical plane relative to the first. The two dioxolane oxygen atoms are anchored at the junction carbon, while the carbon atoms C(1) and C(2) can both pucker (they are not constrained by direct bonds), and it turned out that both configurations exist in the crystal structure. Both rings strongly conform to the envelope forms (the cosine form) (with the major form puckering parameters being 0.89 and 0.26). Therefore, we have identified two equally viable forms, with envelope conformations. To accurately model this disorder, we used dual components applying similarity restraints and thermal parameter constraints to each split atom. The populations of these components were freely refined to 75% for the form puckered at C(2) and 25% for that puckered at C(1).

Crystal Data for Compound **38** (M =238.285 g/mol): monoclinic, space group $P2_1$ (no. 4), a = 8.2557(7) Å, b = 7.7366(5) Å, c = 9.6161(6) Å, β = 103.784(7)°, V = 596.50(8) Å^3, Z = 2, T = 99.99(10) K, μ(Cu Kα) = 0.803 mm^{-1}, $Dcalc$ = 1.327 g/cm^3, 11151 reflections measured (9.46° ≤ 2Θ ≤ 154.8°), 2403 unique (R_{int} = 0.0805, R_{sigma} = 0.0588) which were used in all calculations. The final R_1 was 0.0498 (I>=2u(I)) and wR_2 was 0.1278 (all data).

Identification code	YJ02153-VRF
Empirical formula	$C_{13}H_{18}O_4$

Formula weight	238.285
Temperature/K	99.99(10)
Crystal system	monoclinic
Space group	$P2_1$
a/Å	8.2557(7)
b/Å	7.7366(5)
c/Å	9.6161(6)
α/°	90
β/°	103.784(7)
γ/°	90
Volume/Å^3	596.50(8)
Z	2
ρ_{calc}g/cm^3	1.327
μ/mm^{-1}	0.803
F(000)	256.9
Crystal size/mm^3	0.39 × 0.26 × 0.21
Radiation	Cu Kα (λ = 1.54184)
2Θ range for data collection/°	9.46 to 154.8
Index ranges	$-10 \leq h \leq 10$, $-9 \leq k \leq 9$, $-9 \leq l \leq 12$
Reflections collected	11151
Independent reflections	2403 [R_{int} = 0.0805, R_{sigma} = 0.0588]
Data/restraints/parameters	2403/366/281
Goodness-of-fit on F^2	1.088
Final R indexes [I>=2σ (I)]	R_1 = 0.0498, wR_2 = 0.1228
Final R indexes [all data]	R_1 = 0.0550, wR_2 = 0.1278
Largest diff. peak/hole / e Å^{-3}	0.31/-0.23
Flack parameter	-0.08(17)

Part 3. Reference:

1. Bartoli, G.; Cipolletti, R.; Di Antonio G.; Giovannini, R.; Lanari, S.; Marcolini, M.; Marcantoni, E. A convergent approach to (*R*)-Tiagabine by a regio- and stereocontrolled hydroiodination of alkynes. *Org. Biomol. Chem*. **2010**, *8*, 3509−3517.

2. Zhang, Z.; Giampa, G. M.; Draghici, C.; Huang, Q.; Brewer, M. Synthesis of Demissidine by a Ring Fragmentation 1,3-Dipolar Cycloaddition Approach. *Org. Lett.* **2013**, *15*, 2100–2103.

3. Seel, S.; Thaler, T.; Takatsu, K.; Zhang, C.; Zipse, H.; Straub, B. F.; Mayer, P.; Knochel, P. Highly Diastereoselective Arylations of Substituted Piperidines. *J. Am. Chem. Soc.* **2011**, *133*, 4774−4777.

4. Zorn, N.; Lett, R. Enol triflates derived from the Wieland−Miescher ketone and an analog bearing an angular acetoxymethyl group: their highly regioselective synthesis and Stille coupling with vinyl(tributyl)tin. *Tetrahedron Lett.* **2006**, *47*, 4331−4335.

5. Ciceri, P.; Demnitz, F. W. J. An Efficient, Rapid and Highly Selective Preparation of the Wieland-Miescher Ketone 9-Ehylene Ketal. *Tetrahedron Lett.* **1997**, *38*, 389–390.

6. Zheng, C.-Y.; Yue, J.-M. Allylic hydroxylation of enones useful for the functionalization of relevant drugs and natural products. *Nat. Commun.* **2023**, *14*, 2399.

7. Cassaidy, K. J.; Rawal, V. H. Enantioselective Total Synthesis of (+)-Heilonine. *J. Am. Chem. Soc.* **2021**, *143*, 16394−16400.

8. Kitamura, Y.; Nishizawa, M.; Kaneko, K.; Shiro, M.; Chen, Y.-P.; Hsu, H.-Y. New Steroidal Alkaloids from Fritillaria Ussuriensis Maxim. Pingbeinone and Heilonine. *Tetrahedron* **1989**, *45*, 7281−7286.

Part 4. ^{1}H and ^{13}C NMR spectra

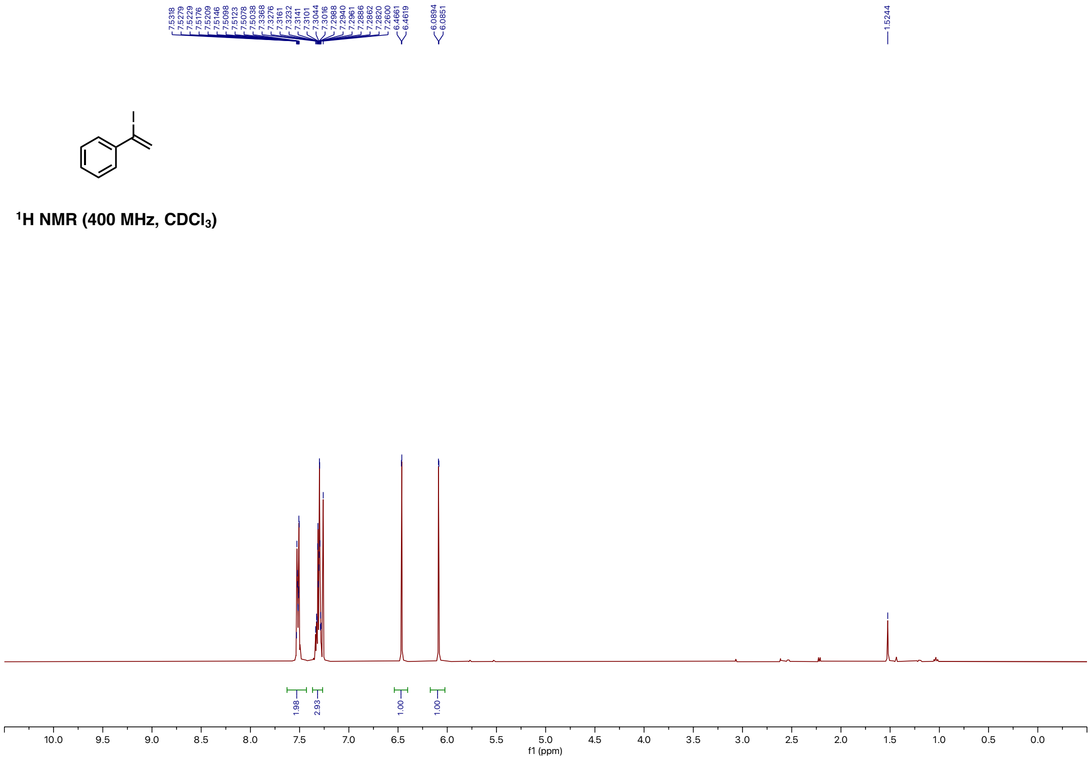

^{1}H NMR (400 MHz, $CDCl_3$)

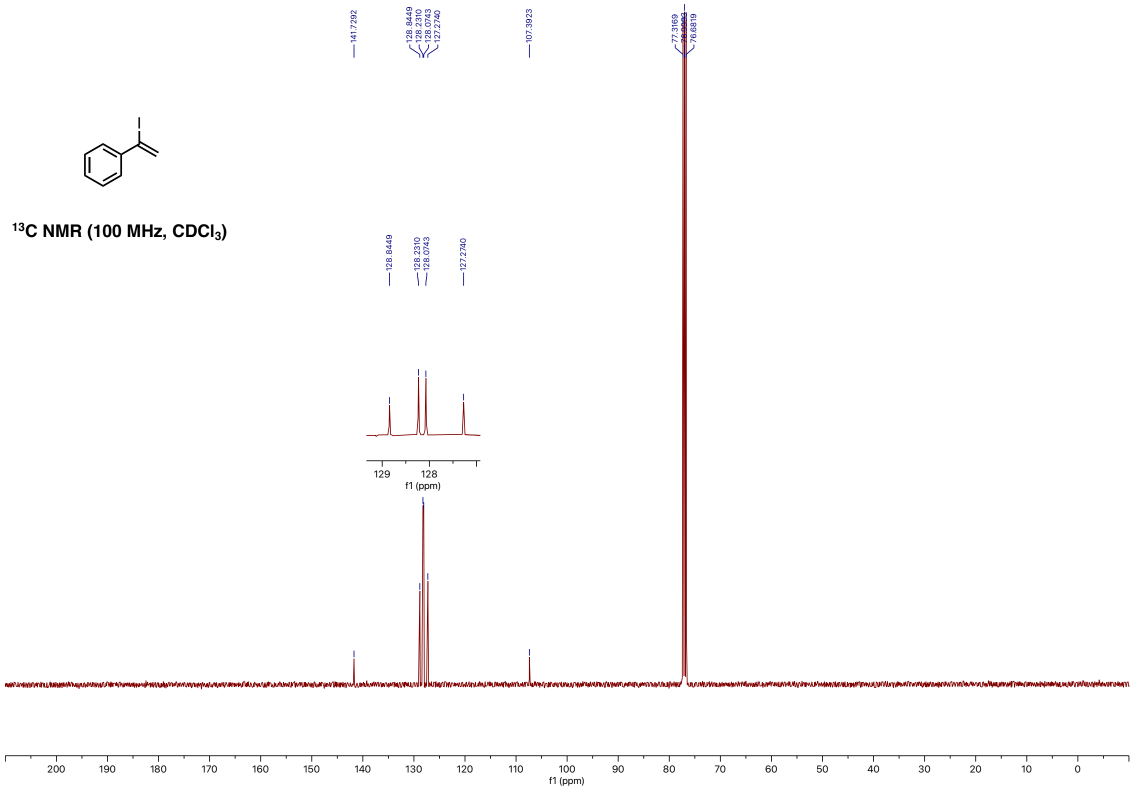

^{13}C NMR (100 MHz, $CDCl_3$)

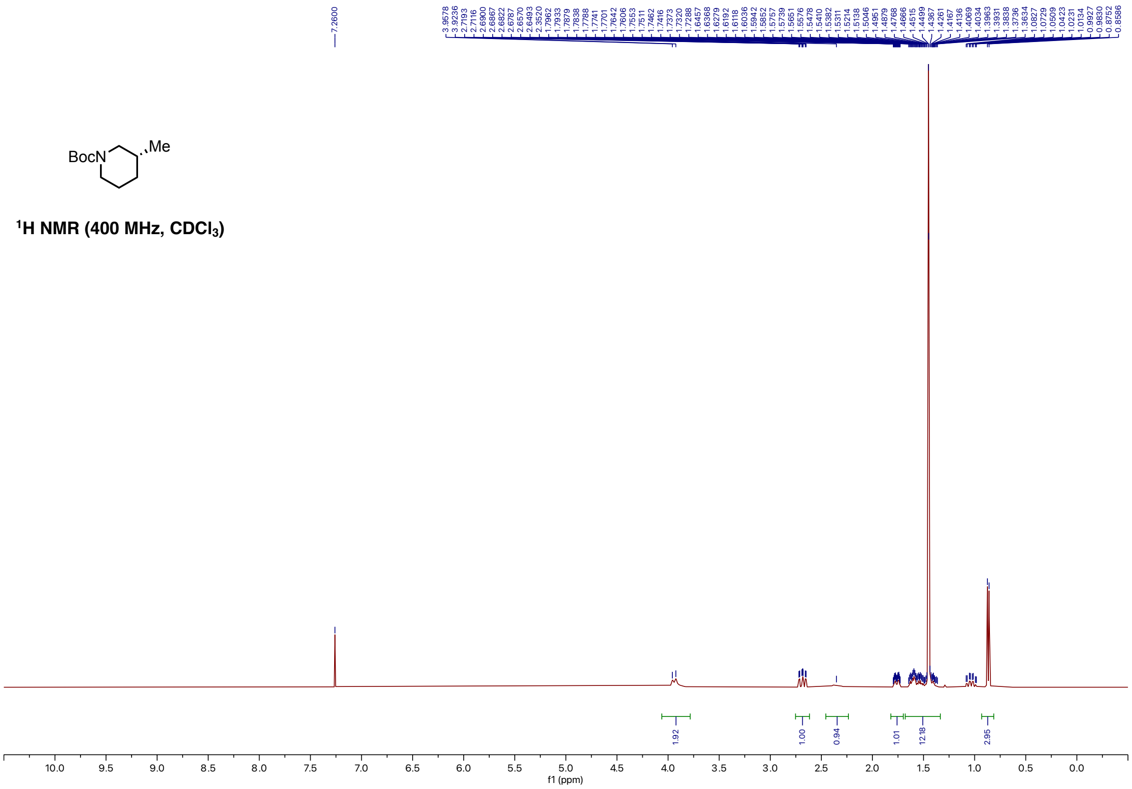

^1H NMR (400 MHz, $CDCl_3$)

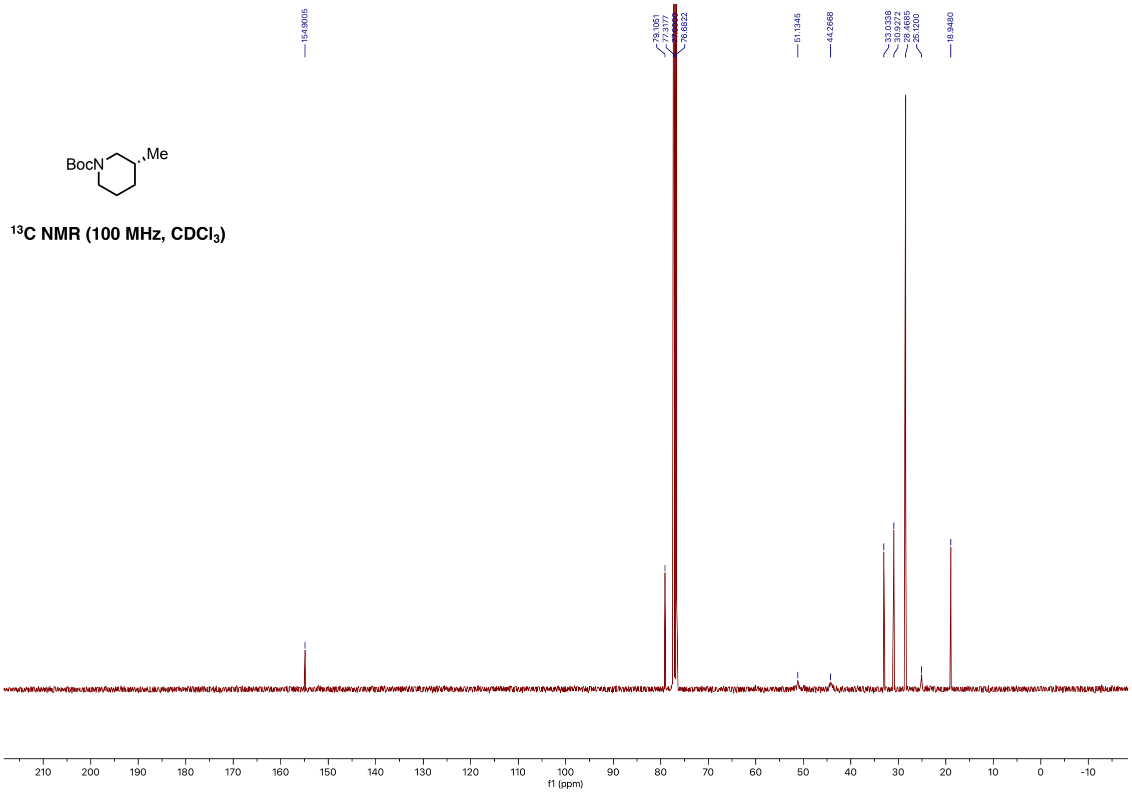

^{13}C NMR (100 MHz, $CDCl_3$)

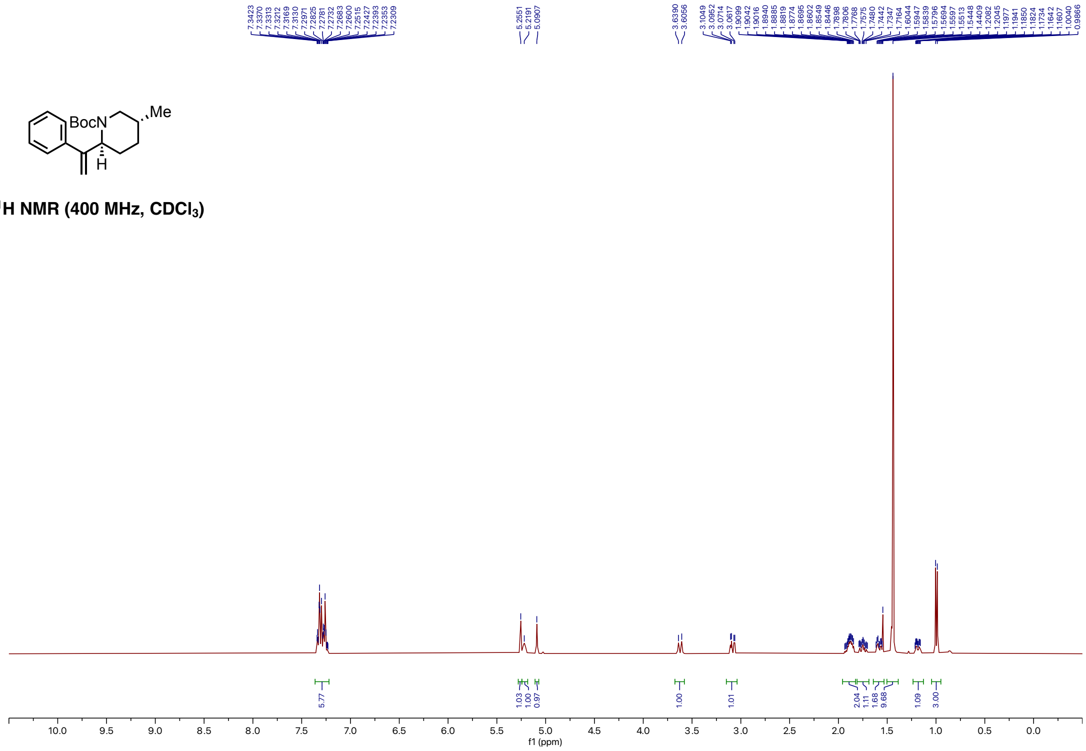

^{1}H NMR (400 MHz, $CDCl_3$)

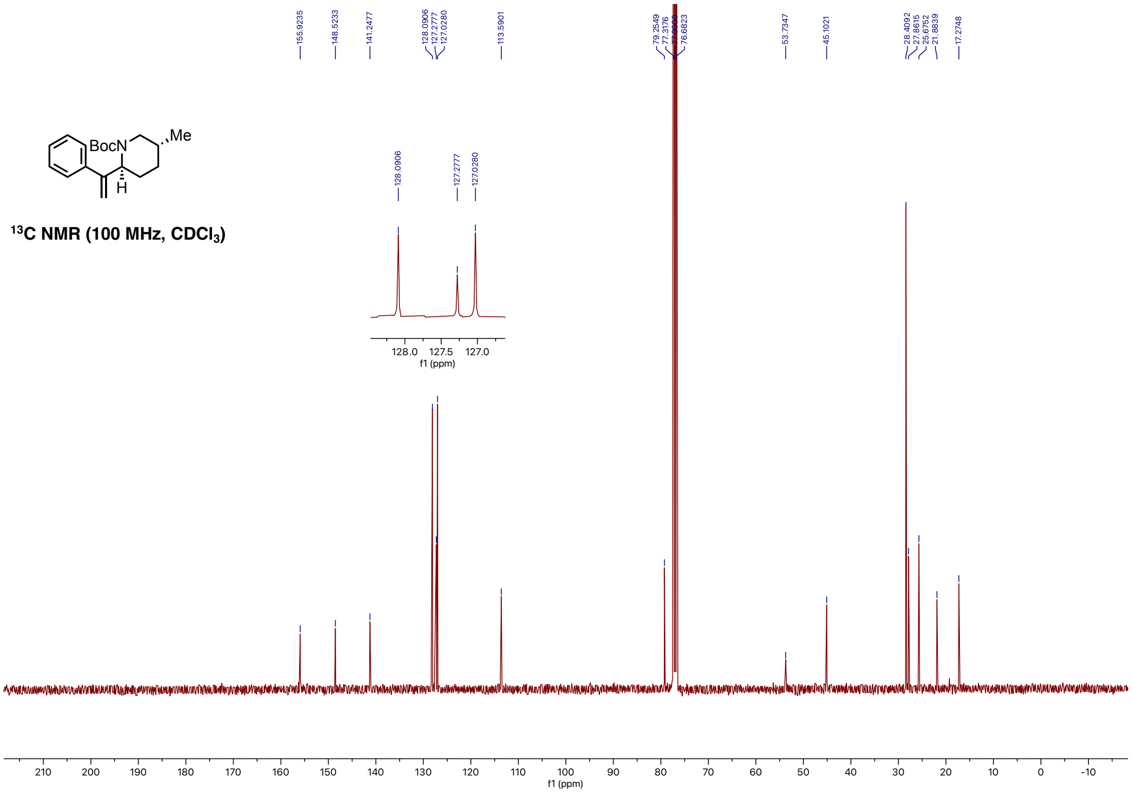

^{13}C NMR (100 MHz, $CDCl_3$)

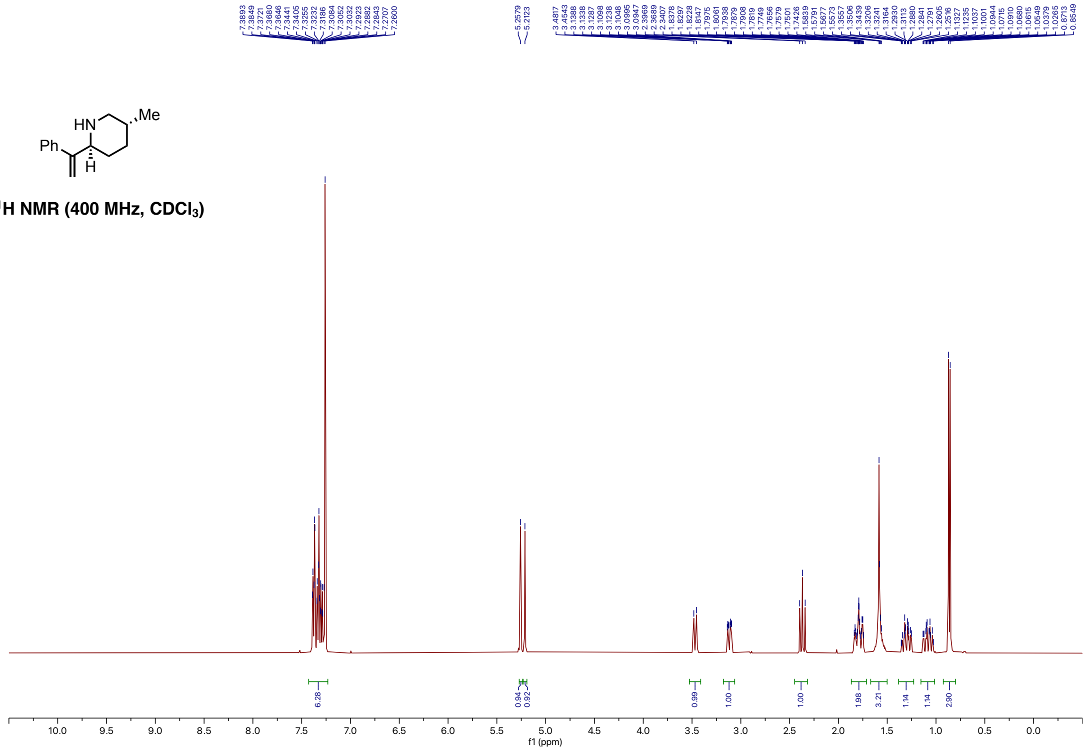

^{1}H NMR (400 MHz, $CDCl_3$)

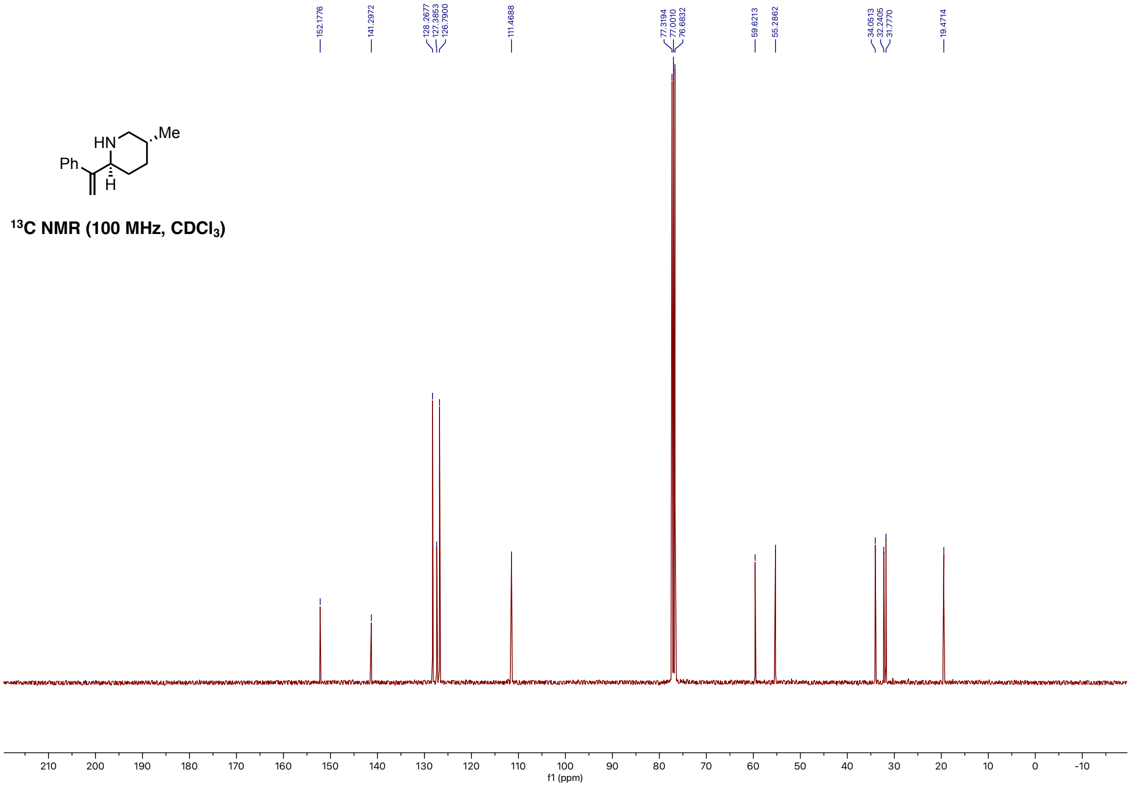

^{13}C NMR (100 MHz, $CDCl_3$)

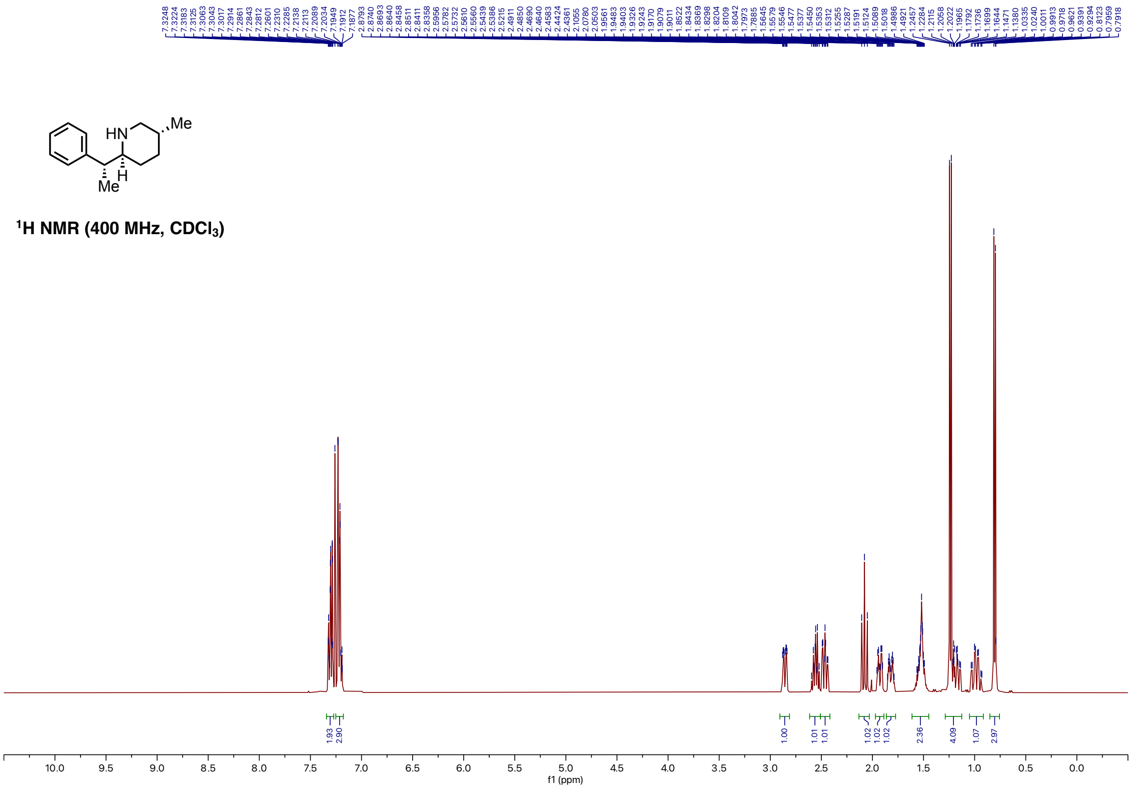

HN
Me
H
Me
^{1}H NMR (400 MHz, $CDCl_3$)
f1 (ppm)

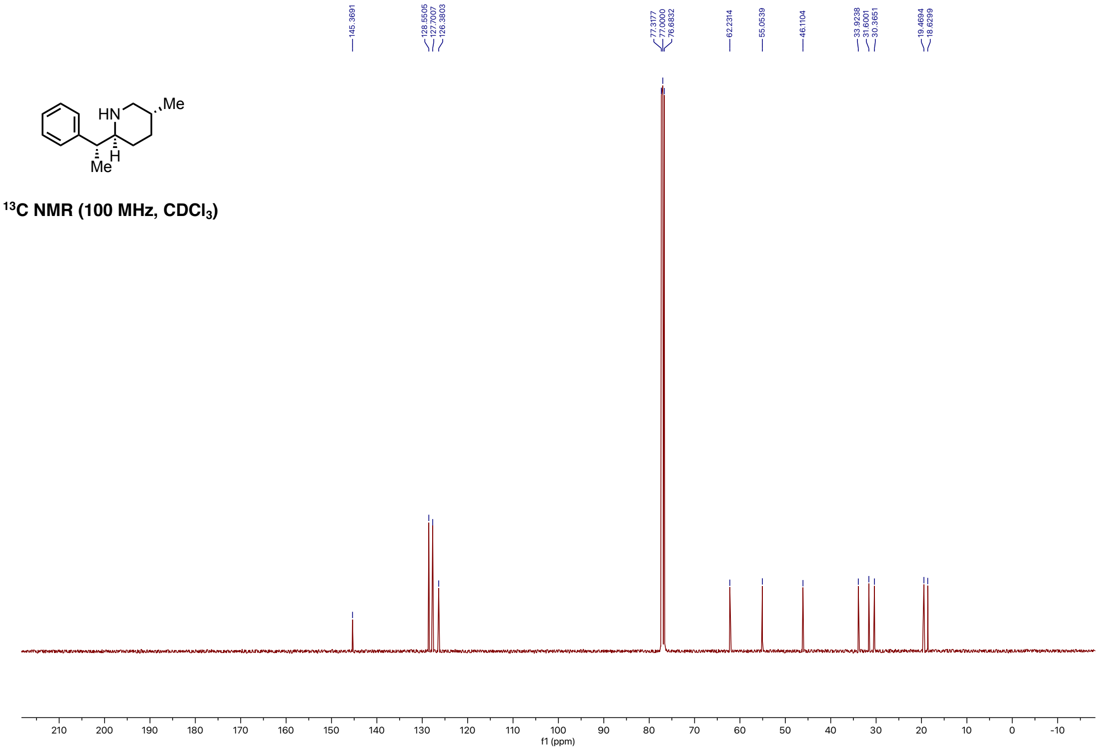

HN
Me
H
Me
^{13}C NMR (100 MHz, $CDCl_3$)
f1 (ppm)

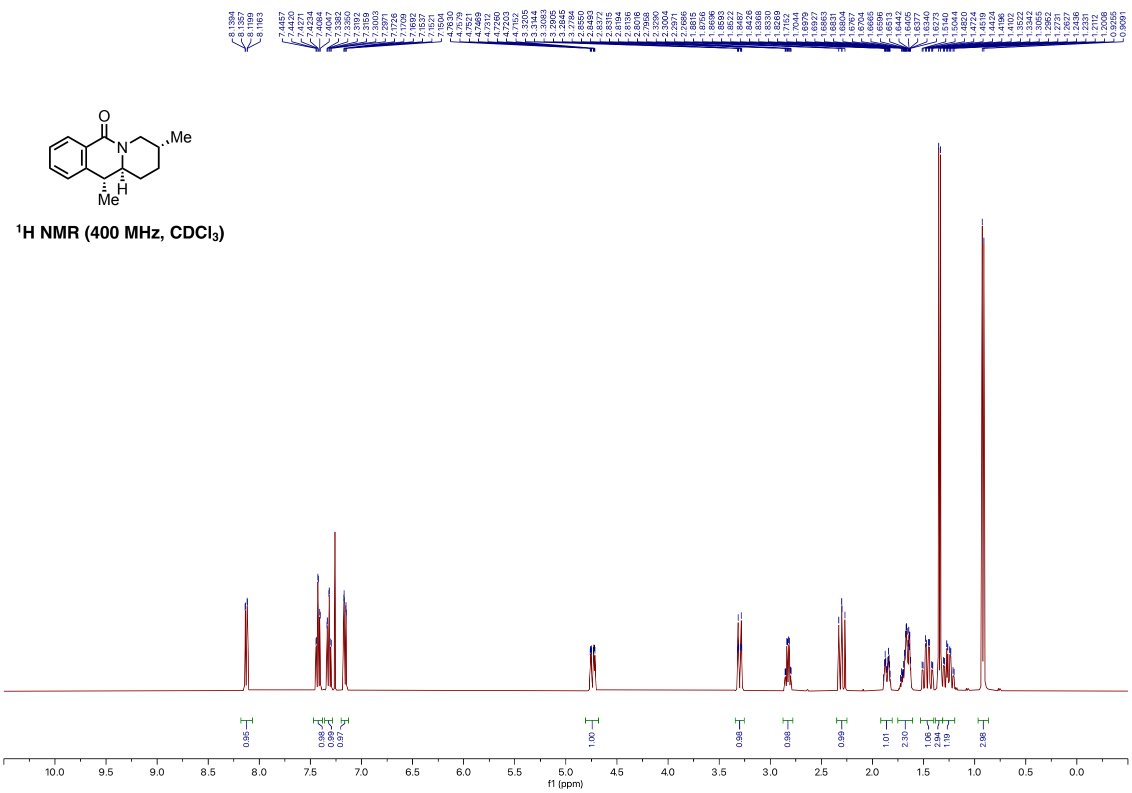
Me
Me
H
N
O
¹H NMR (400 MHz, CDCl₃)
f1 (ppm)

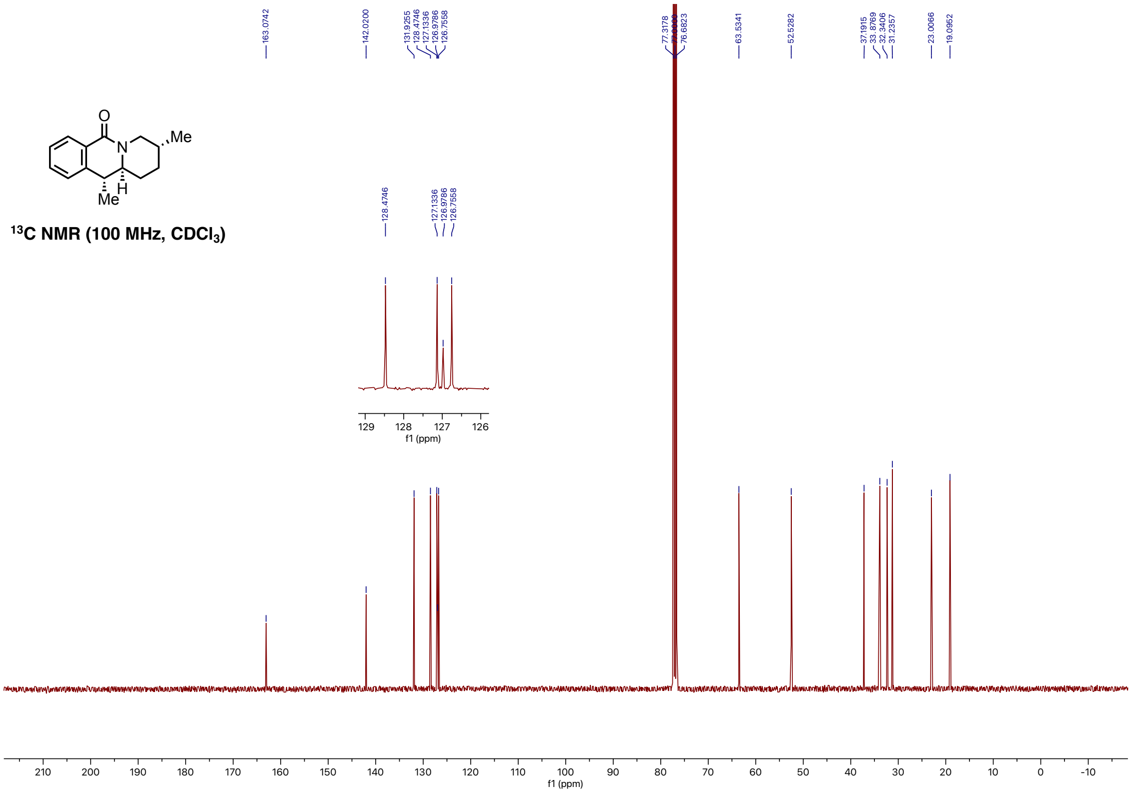
Me
Me
H
N
O
¹³C NMR (100 MHz, CDCl₃)
f1 (ppm)

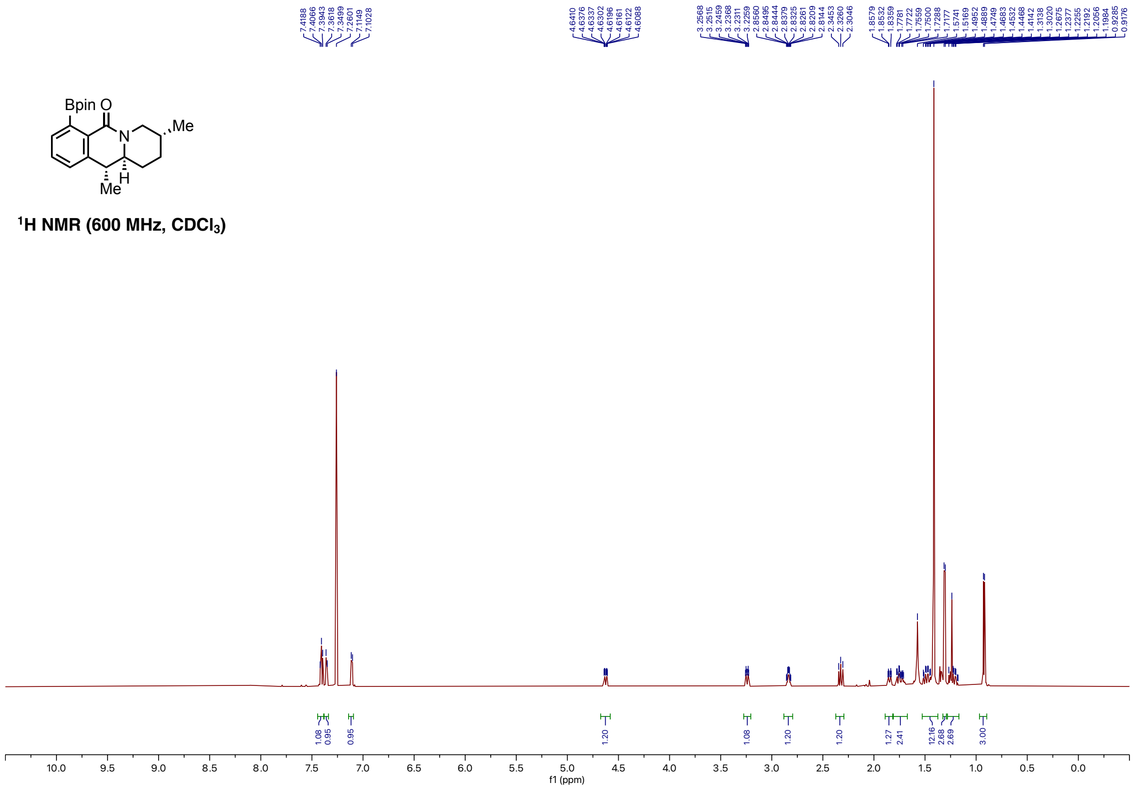

^{1}H NMR (600 MHz, $CDCl_3$)

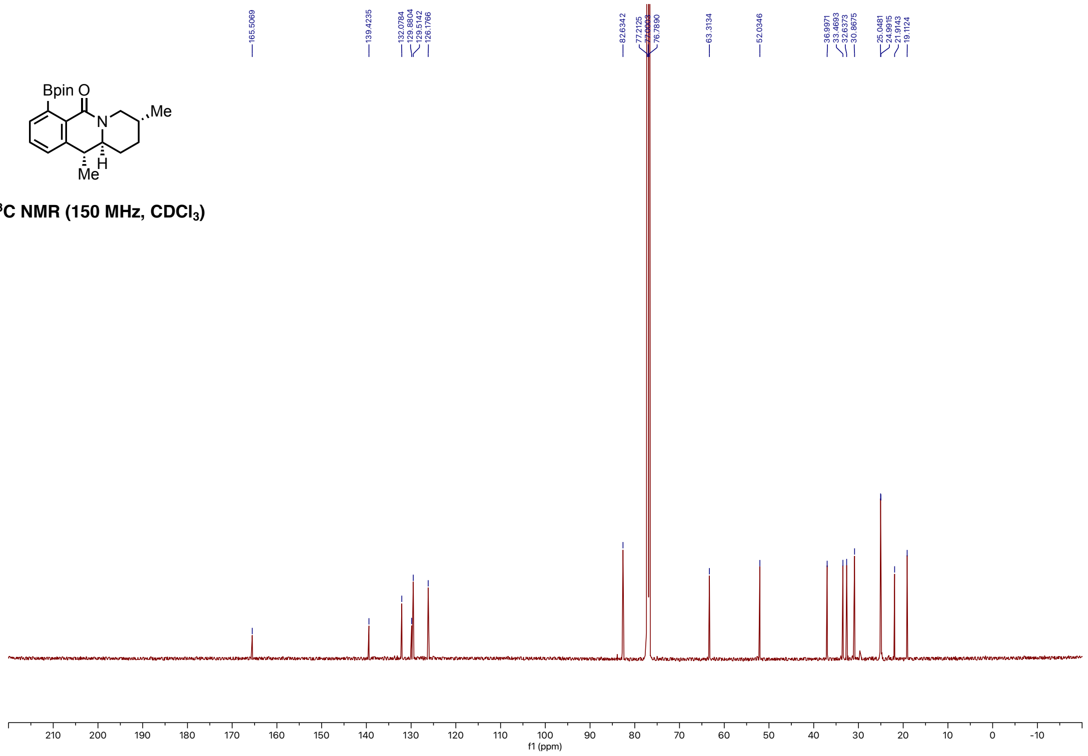

^{13}C NMR (150 MHz, $CDCl_3$)

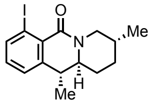

^{1}H NMR (400 MHz, CDCl$_3$)

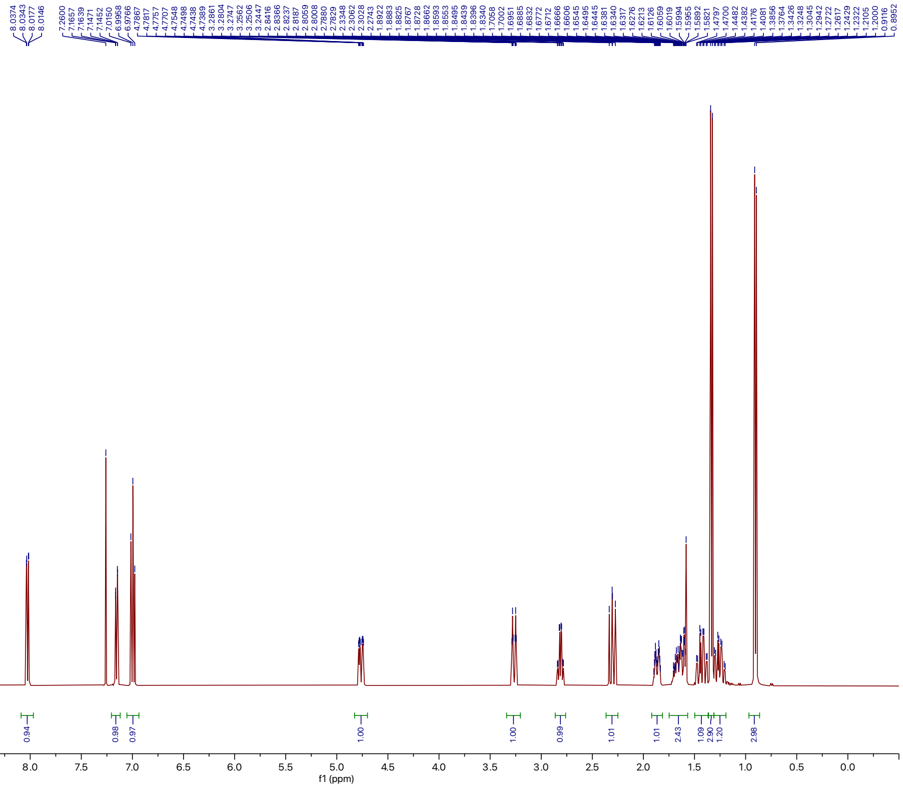

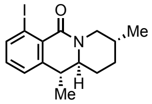

^{13}C NMR (100 MHz, CDCl$_3$)

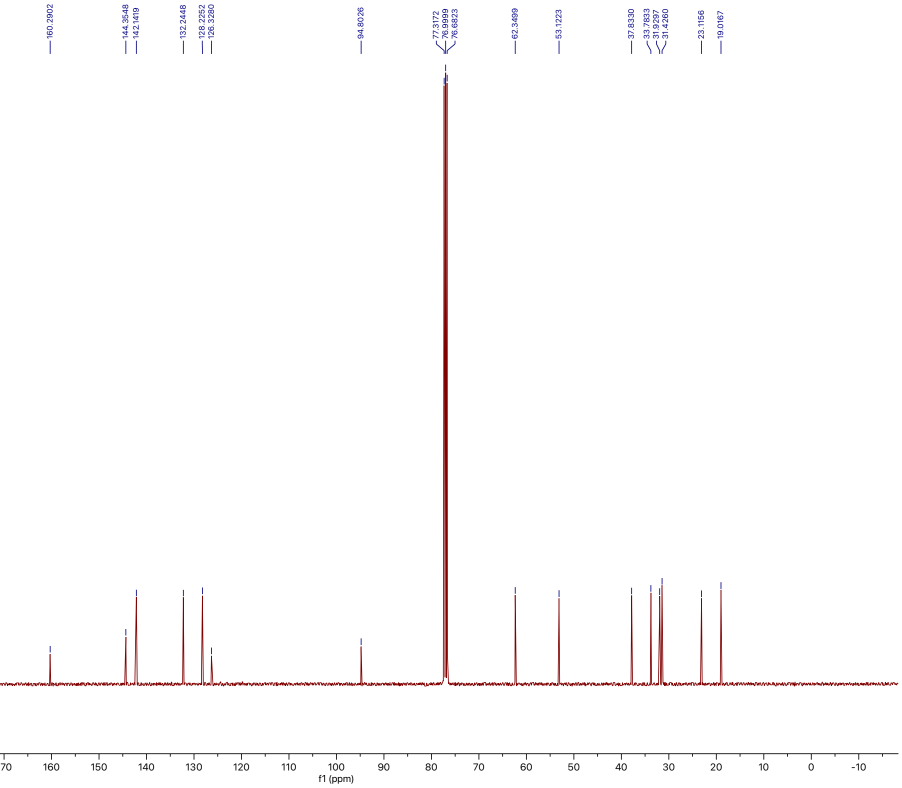

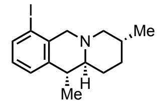

^{1}H NMR (400 MHz, $CDCl_3$)

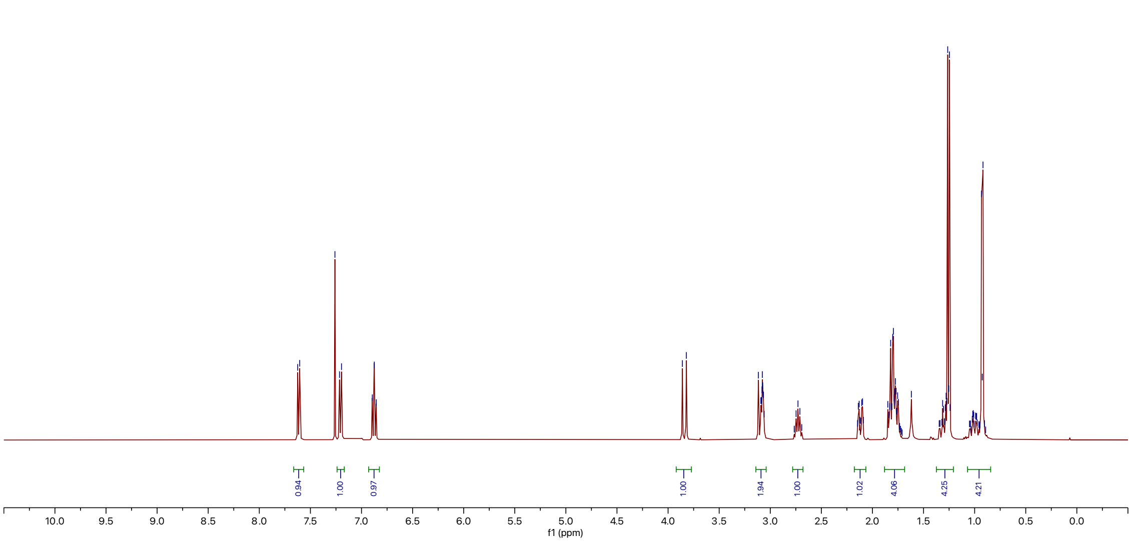

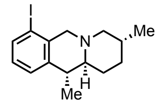

^{13}C NMR (100 MHz, $CDCl_3$)

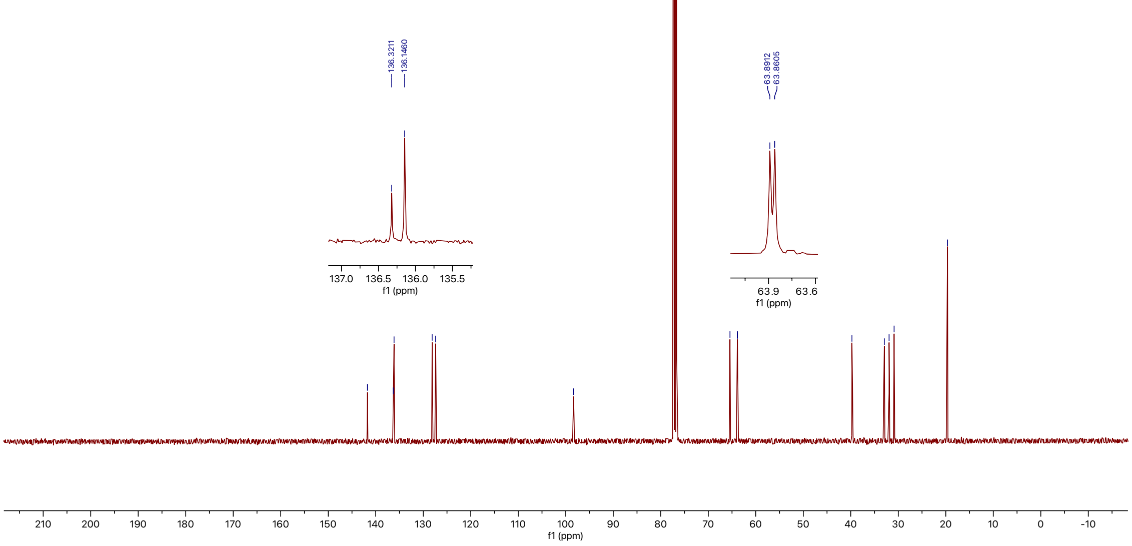

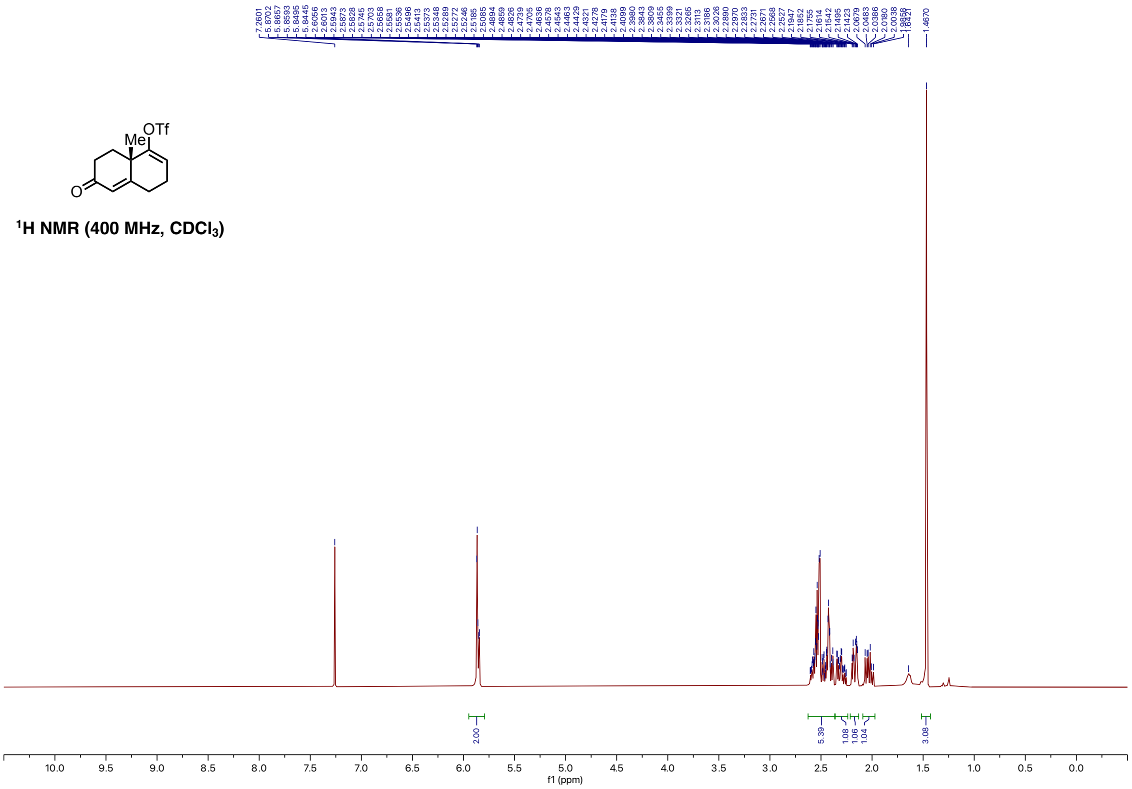
Me
OTf
O
^{1}H NMR (400 MHz, $CDCl_3$)
f1 (ppm)

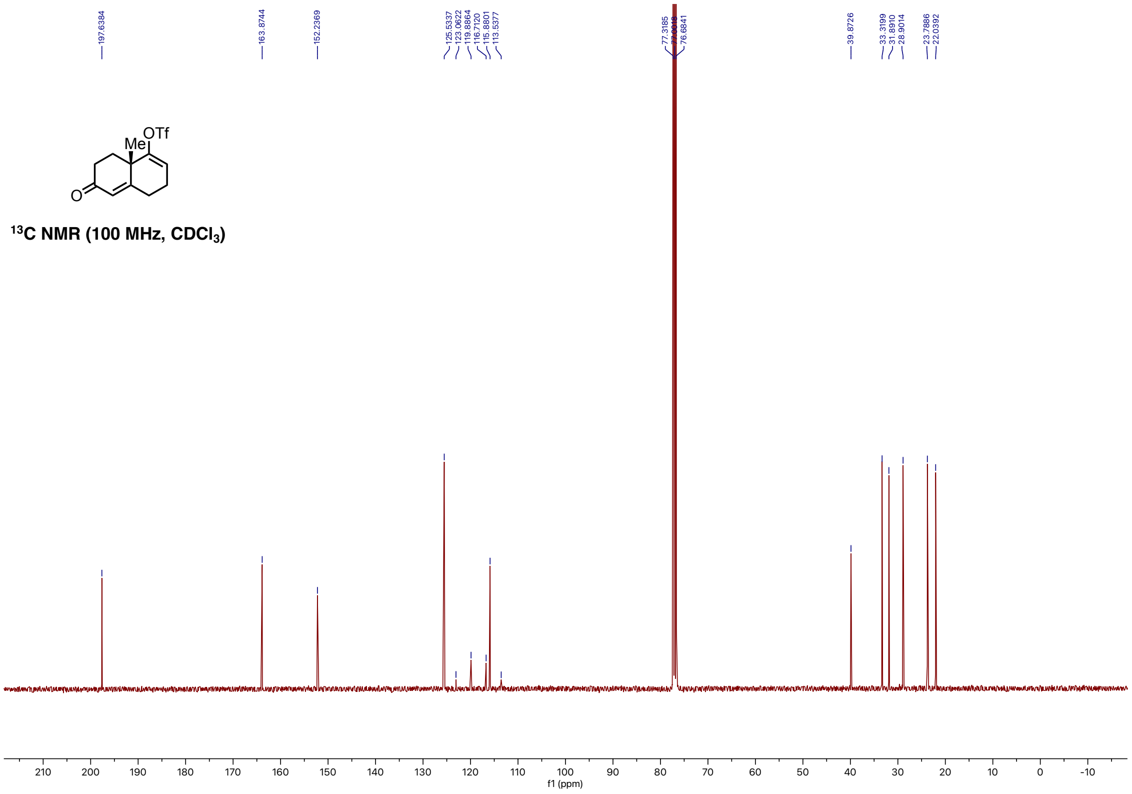
Me
OTf
O
^{13}C NMR (100 MHz, $CDCl_3$)
f1 (ppm)

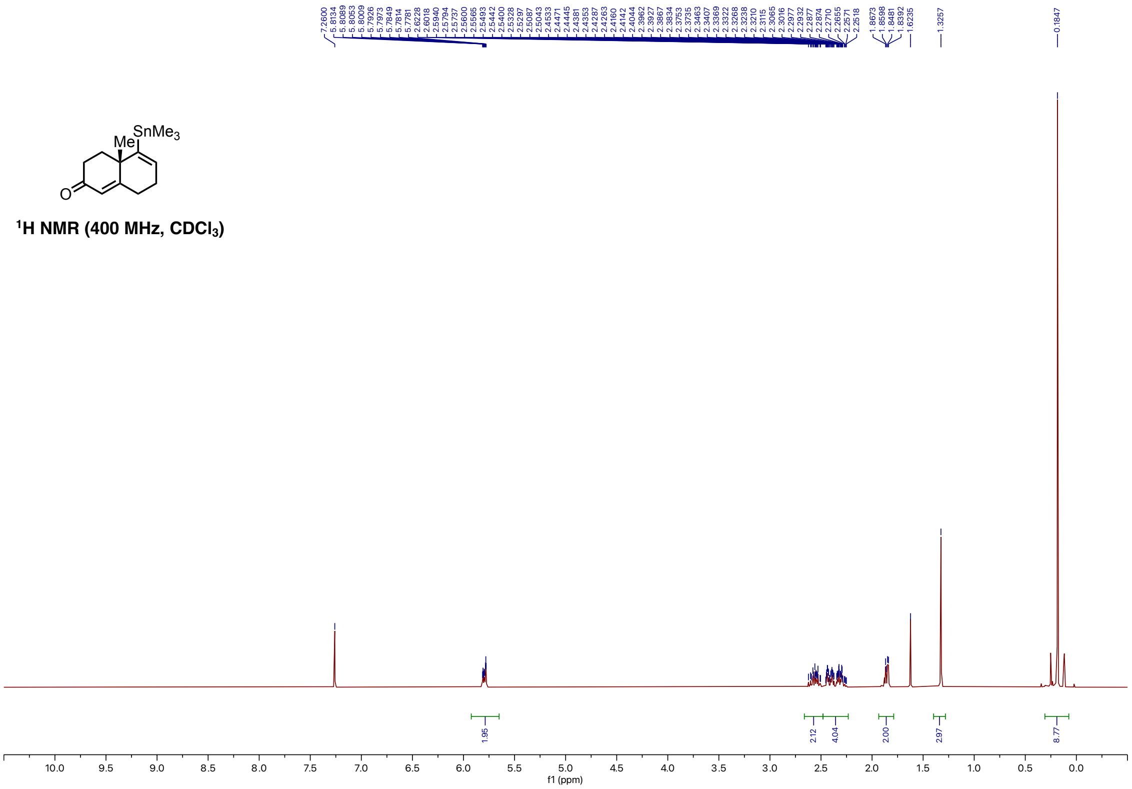
SnMe3
Me
O
1H NMR (400 MHz, CDCl3)
f1 (ppm)

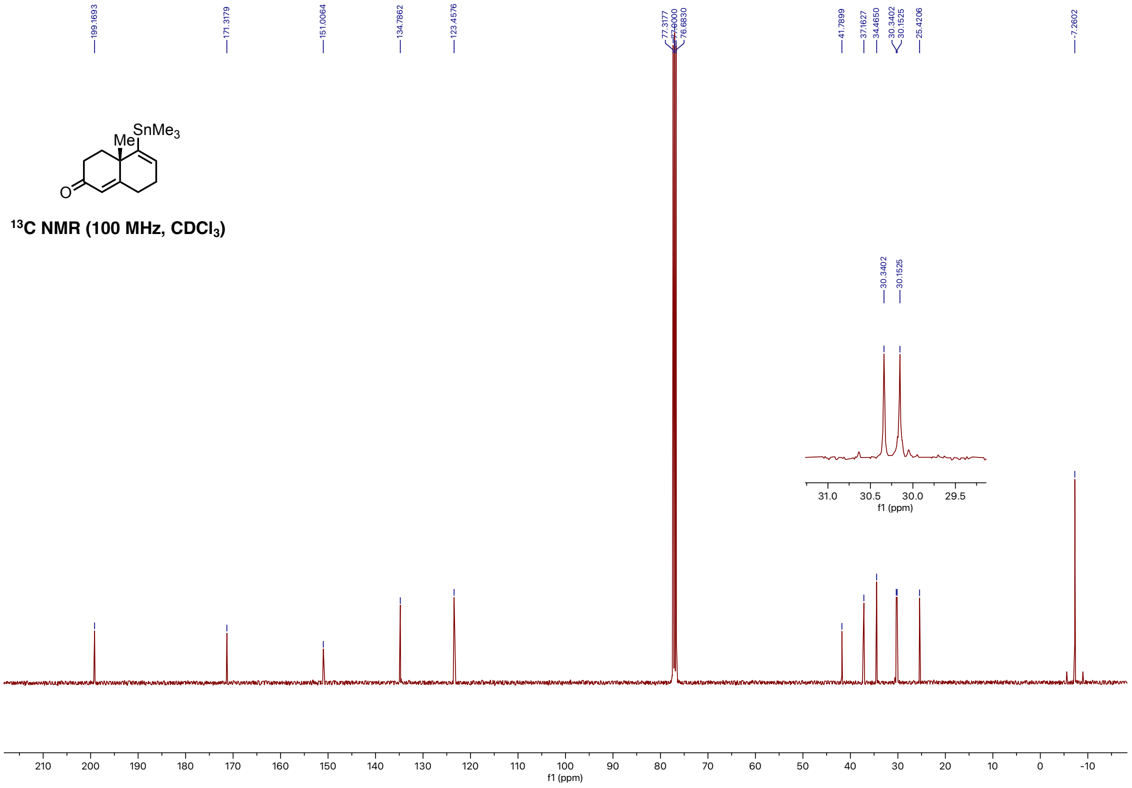
SnMe3
Me
O
13C NMR (100 MHz, CDCl3)
f1 (ppm)

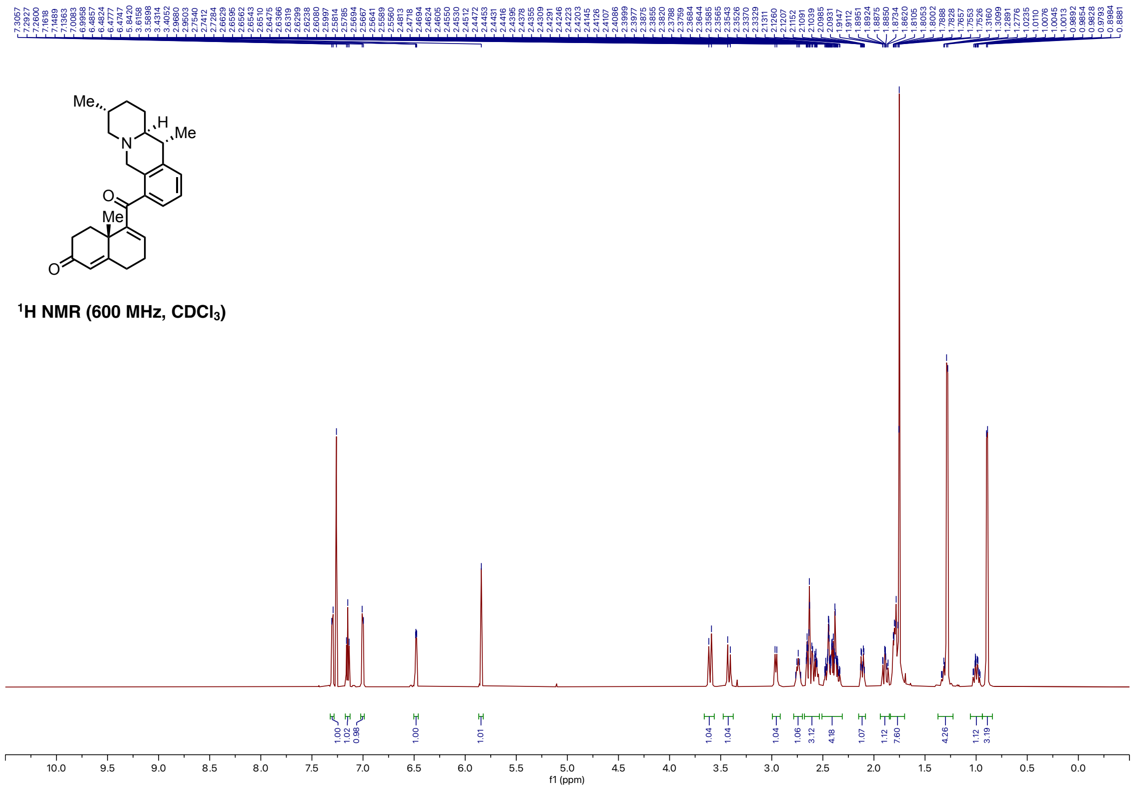

^{1}H NMR (600 MHz, $CDCl_3$)

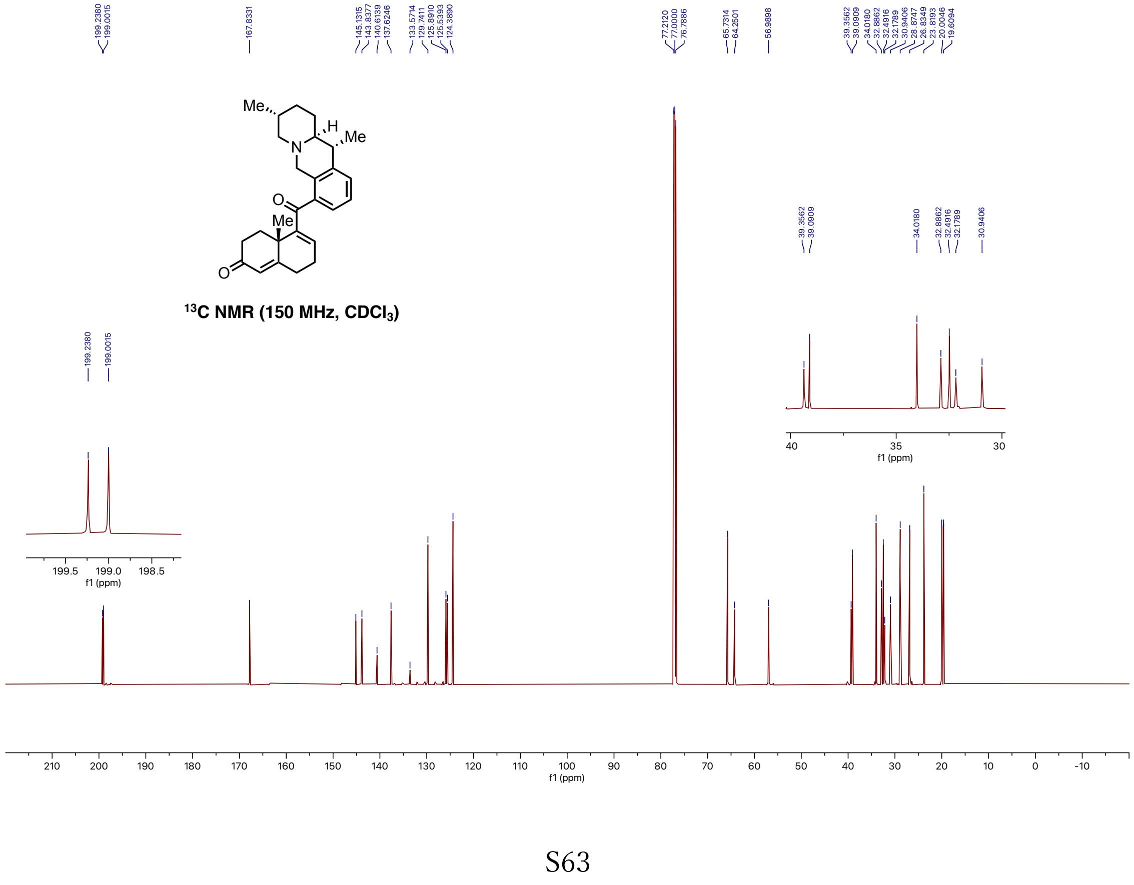

^{13}C NMR (150 MHz, $CDCl_3$)

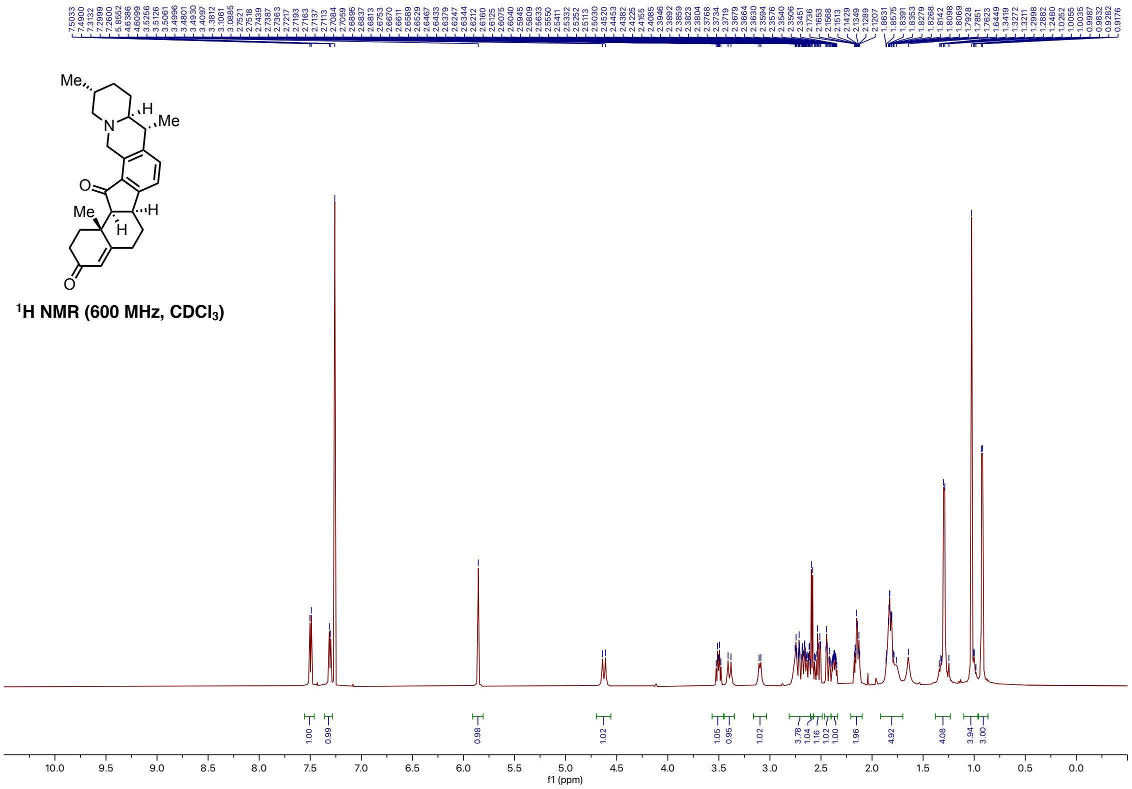

^1H NMR (600 MHz, $CDCl_3$)

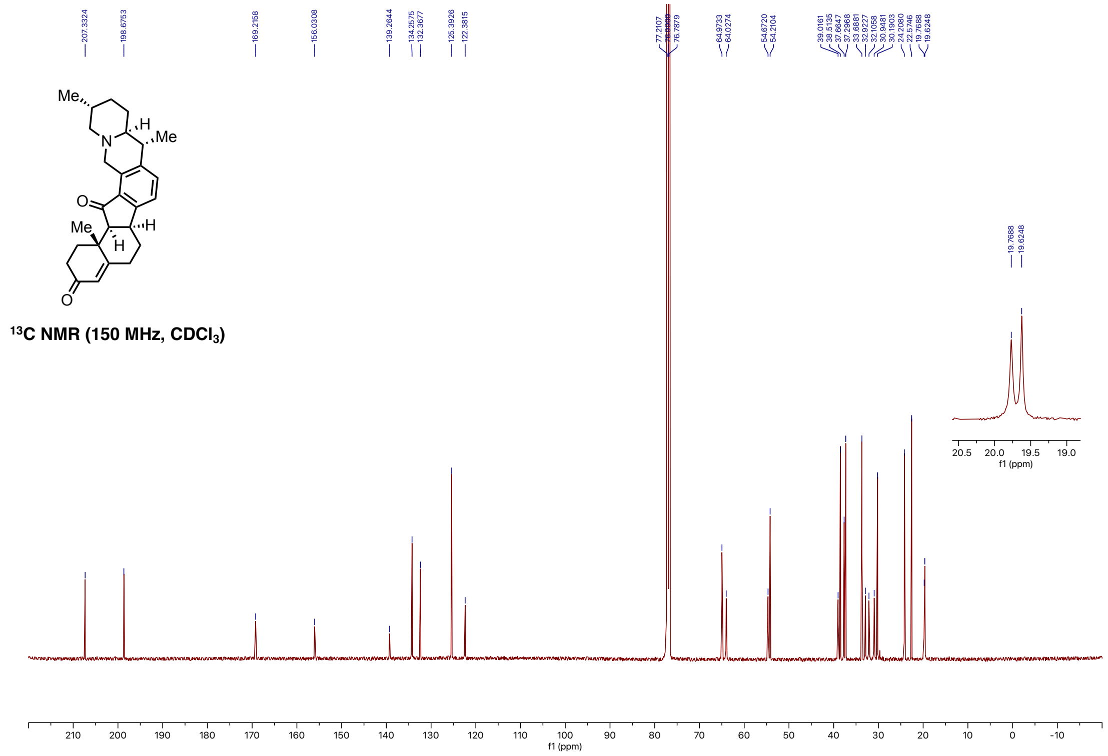

^{13}C NMR (150 MHz, $CDCl_3$)

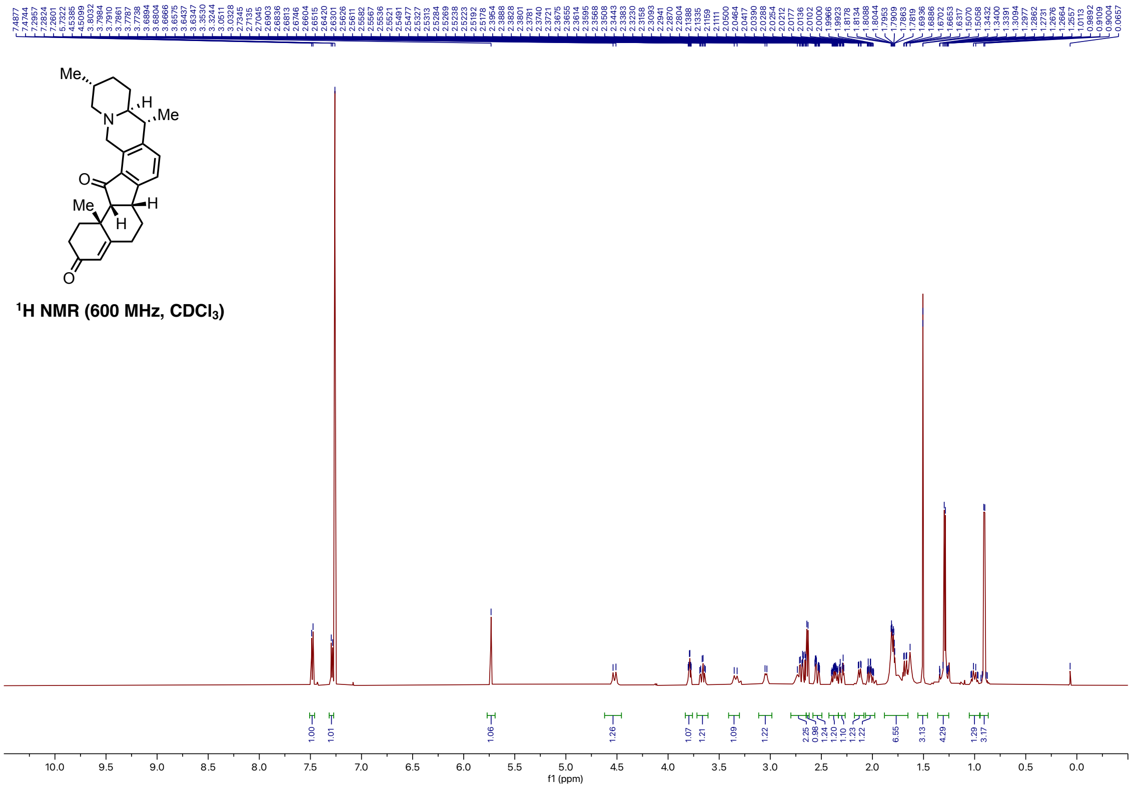

^{1}H NMR (600 MHz, $CDCl_3$)

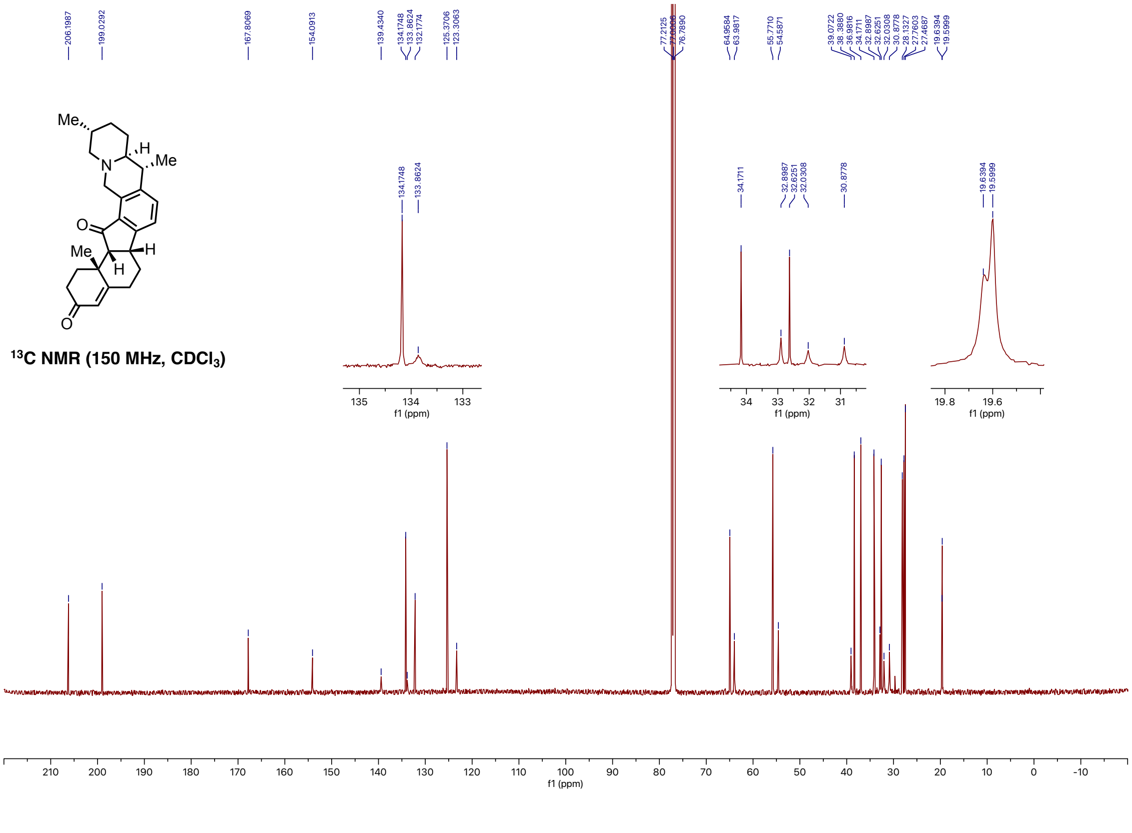

^{13}C NMR (150 MHz, $CDCl_3$)

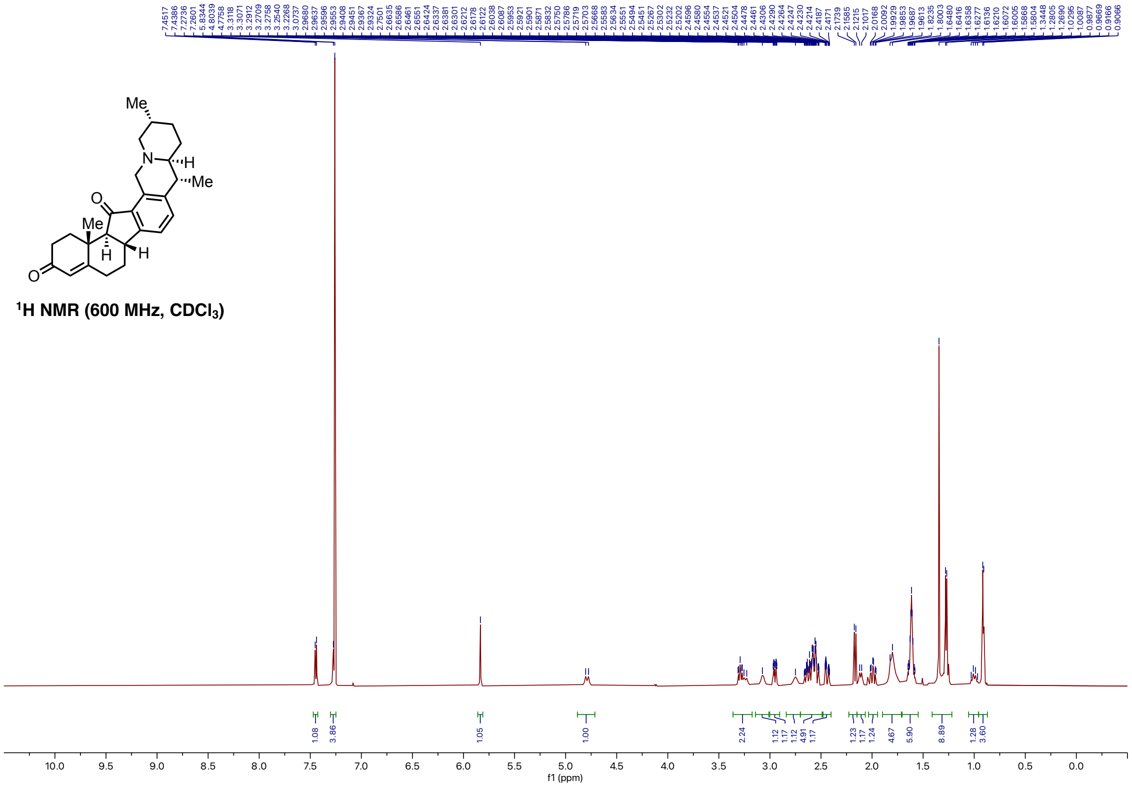

^{1}H NMR (600 MHz, $CDCl_3$)

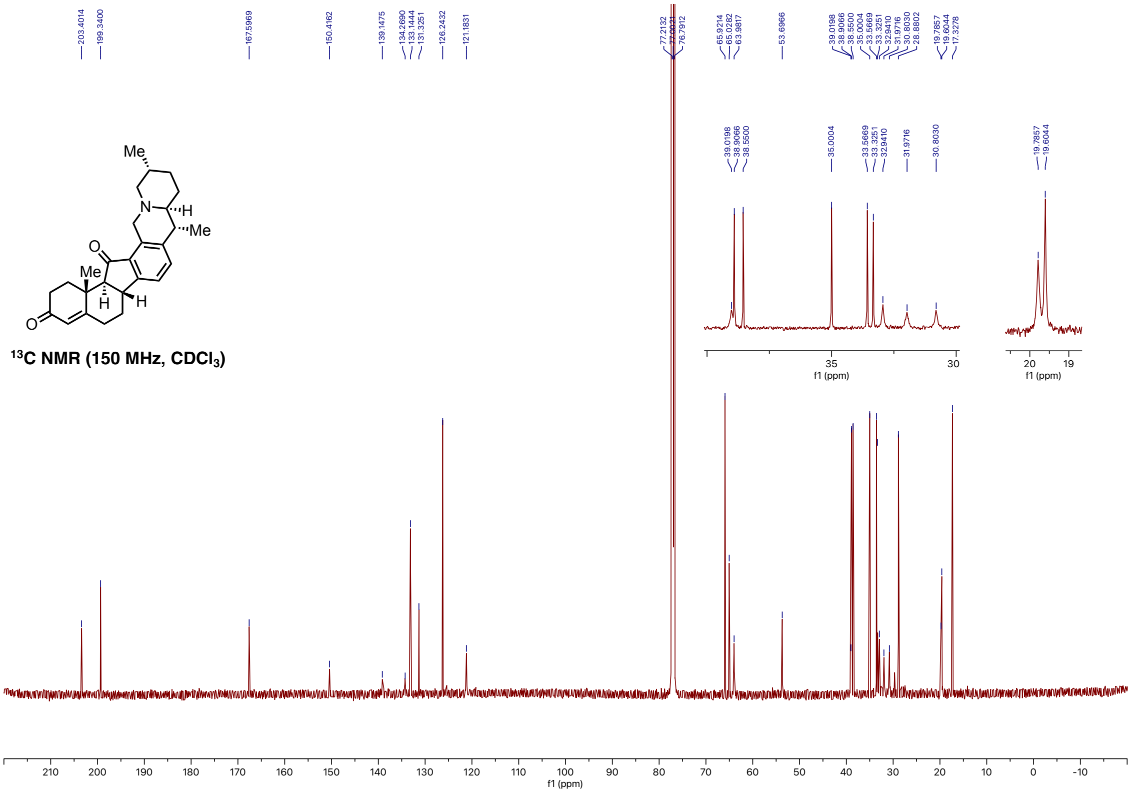

^{13}C NMR (150 MHz, $CDCl_3$)

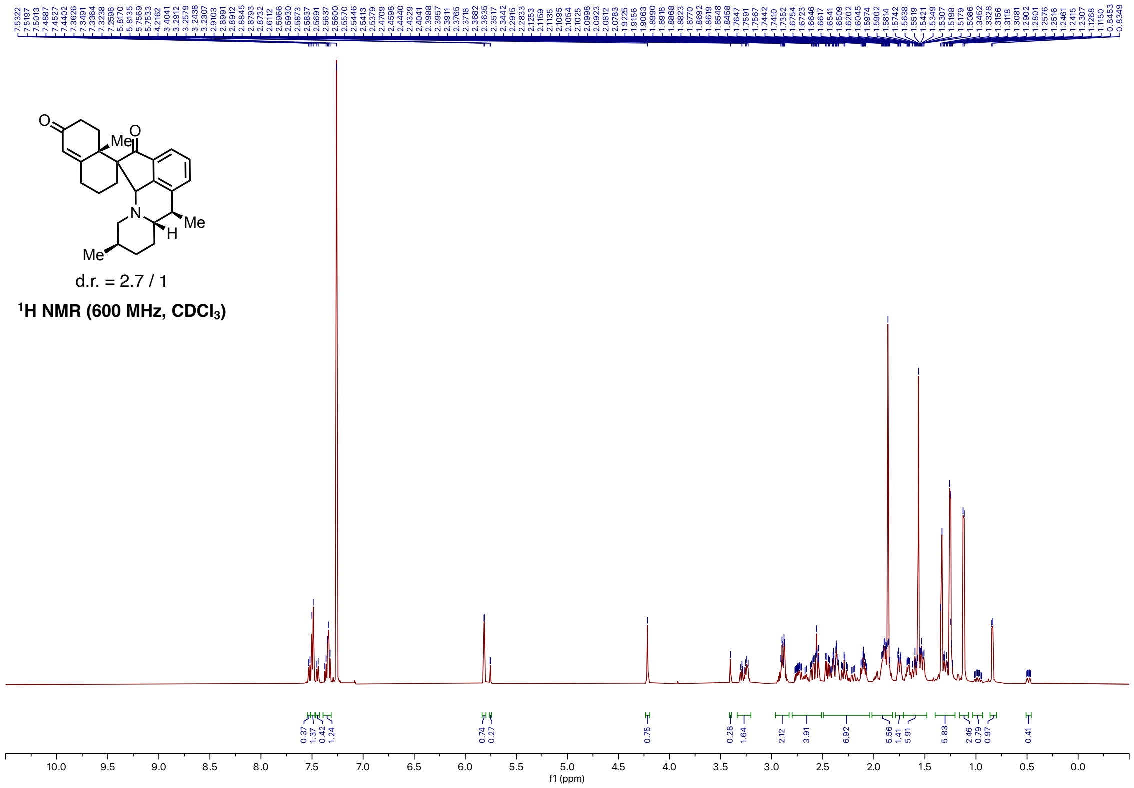
d.r. = 2.7 / 1
1H NMR (600 MHz, CDCl3)
f1 (ppm)

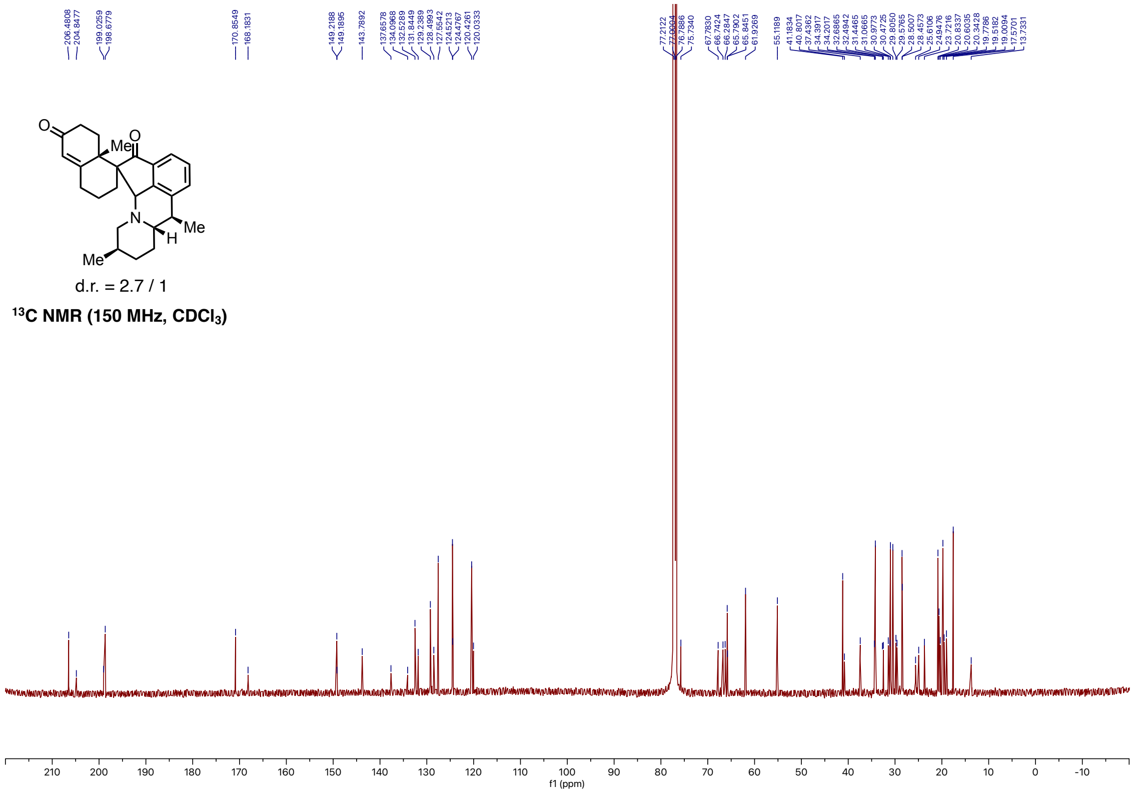
d.r. = 2.7 / 1
13C NMR (150 MHz, CDCl3)
f1 (ppm)

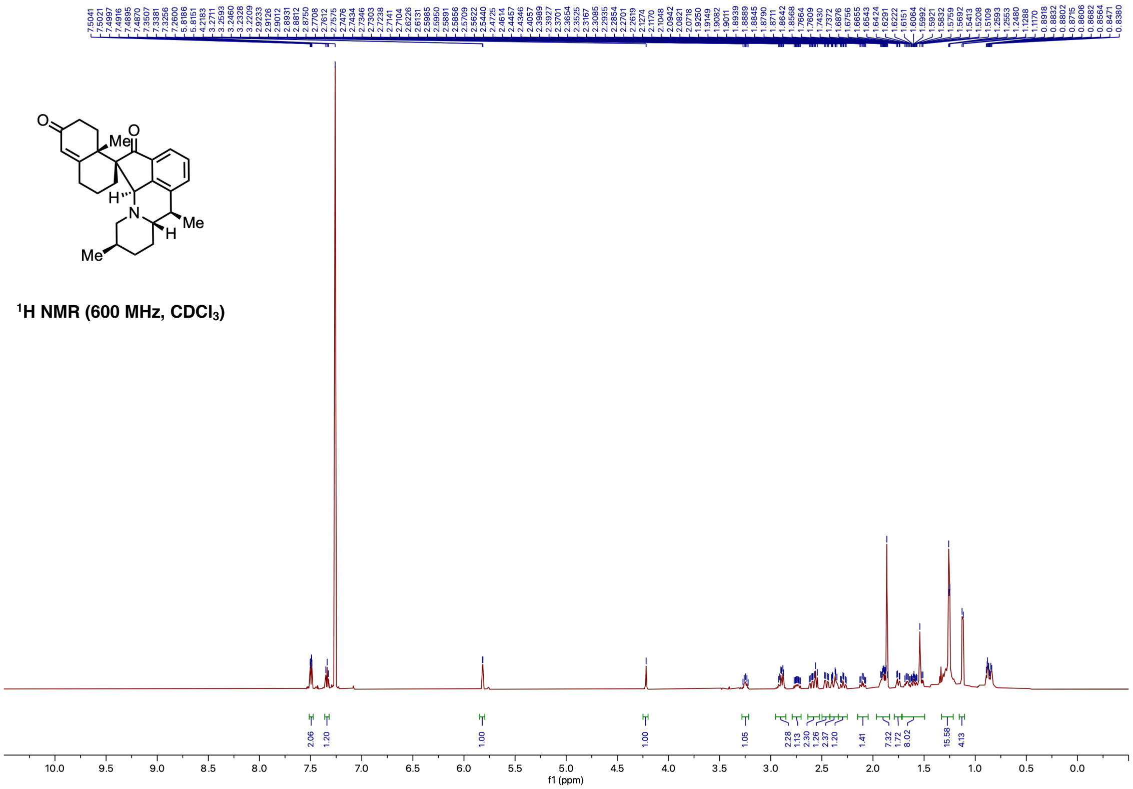

^{1}H NMR (600 MHz, $CDCl_3$)

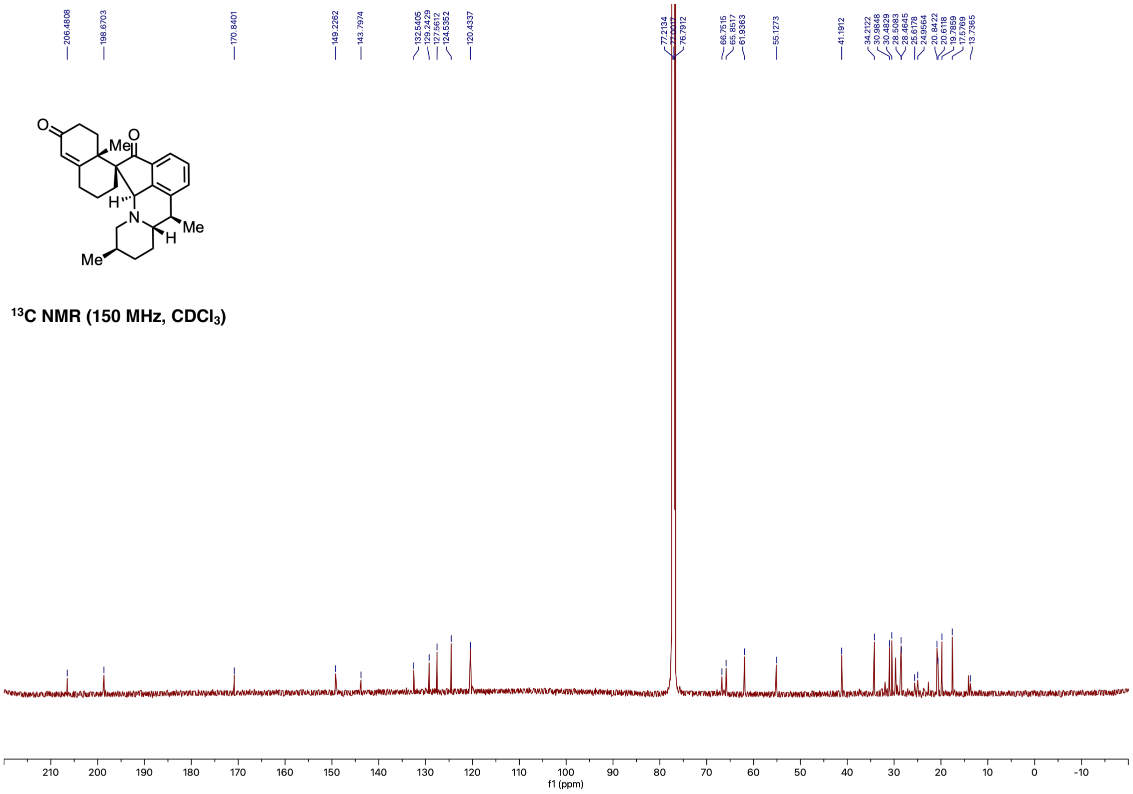

^{13}C NMR (150 MHz, $CDCl_3$)

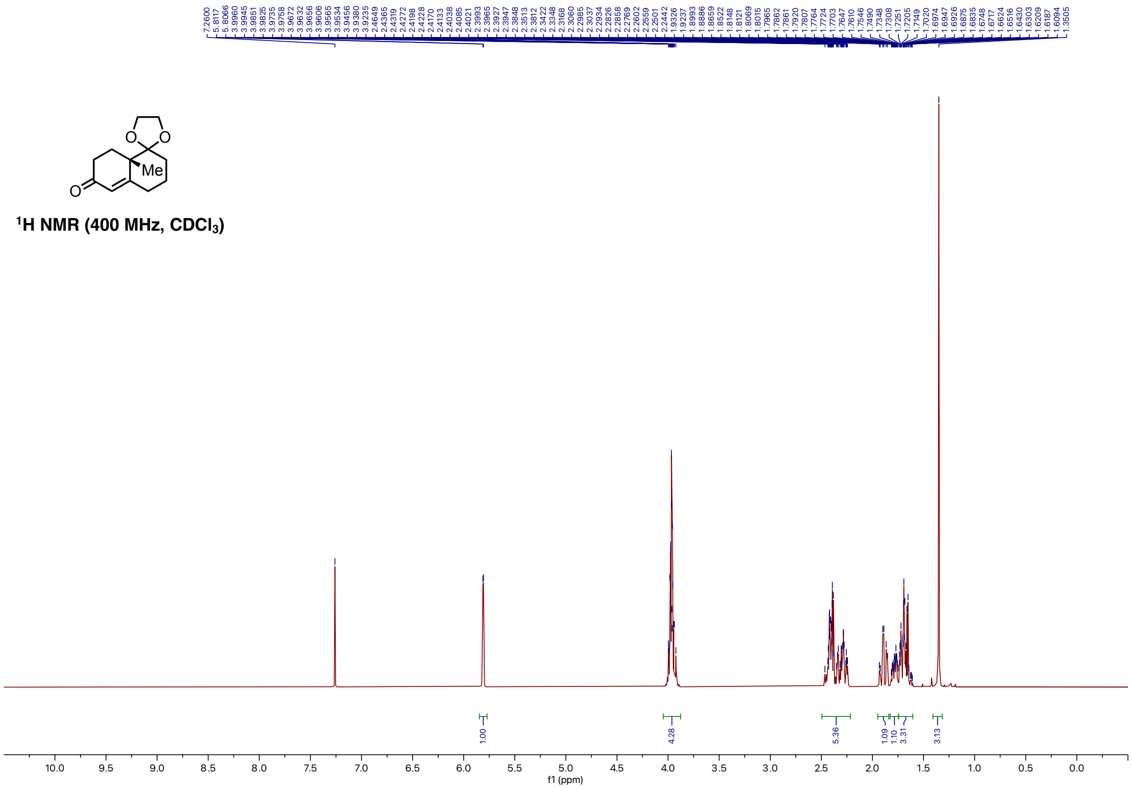

O
O
Me
O
^{1}H NMR (400 MHz, CDCl$_3$)
1.00
4.28
5.36
1.09
1.10
3.31
3.13
f1 (ppm)

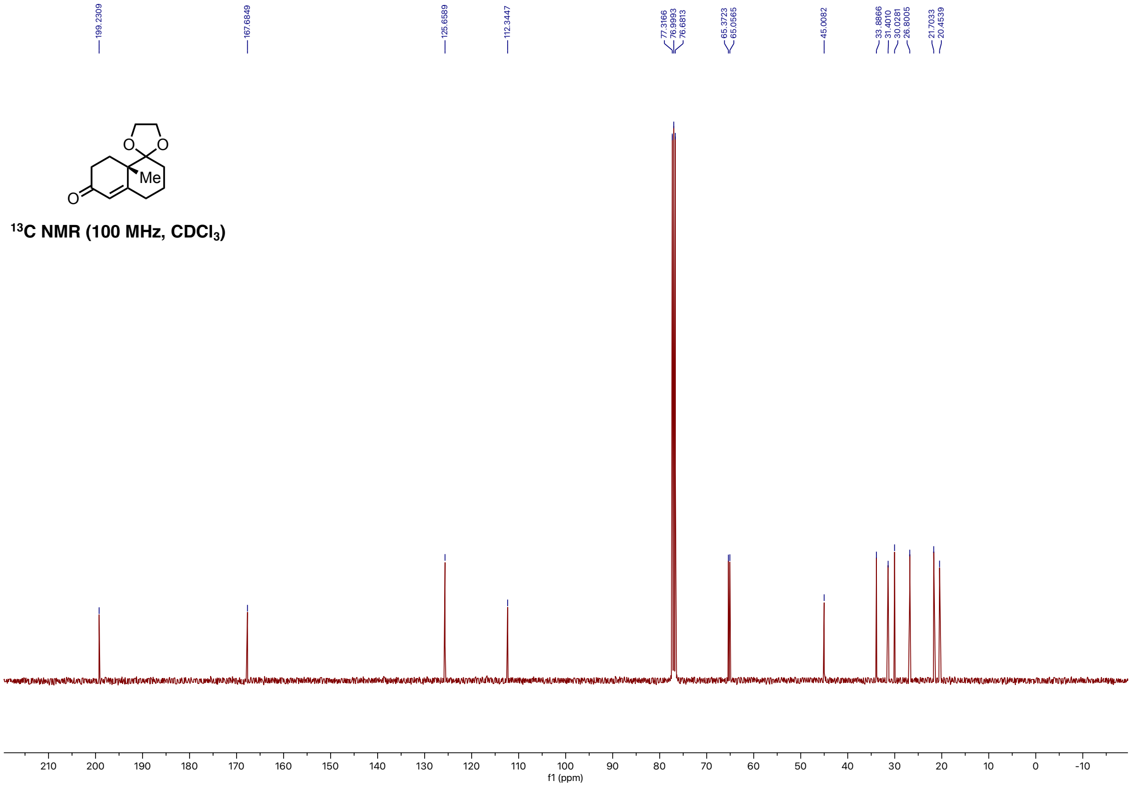

199.2309
167.6849
125.6589
112.3447
77.3166
76.9993
76.6813
65.3723
65.0565
45.0082
33.8866
31.4010
30.0281
26.8005
21.7033
20.4539
O
O
Me
O
^{13}C NMR (100 MHz, CDCl$_3$)
f1 (ppm)

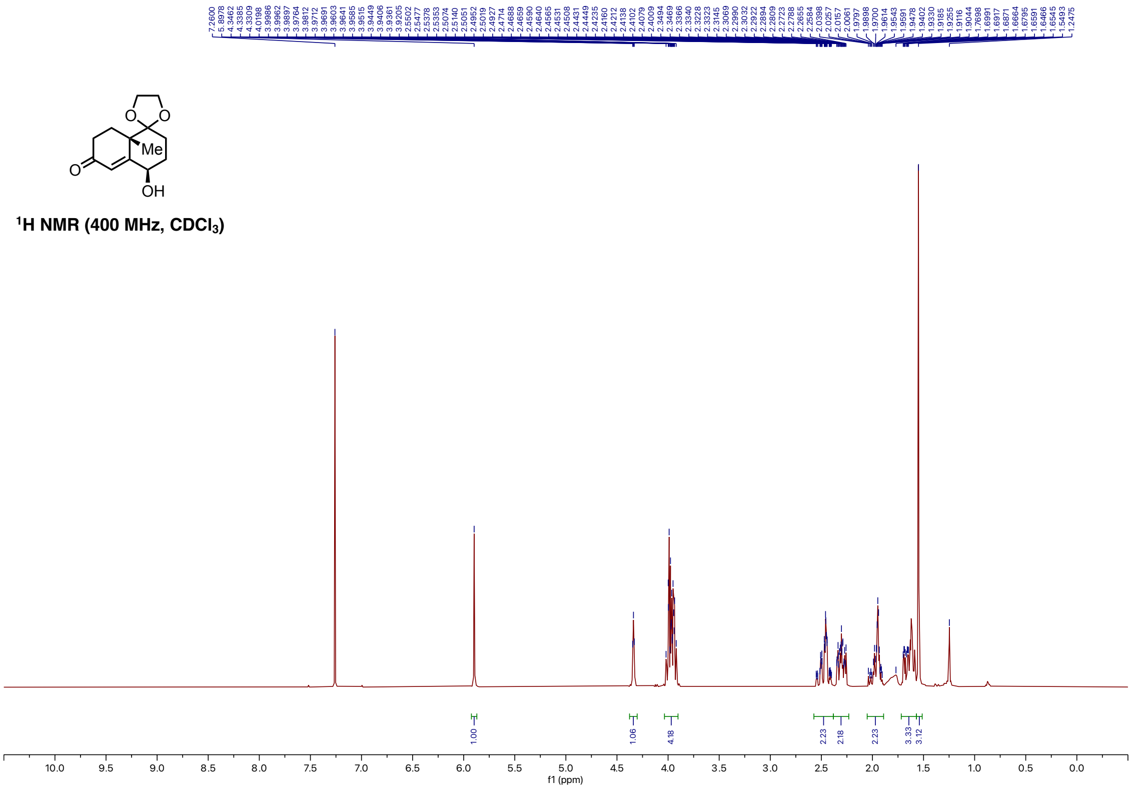

^{1}H NMR (400 MHz, $CDCl_3$)

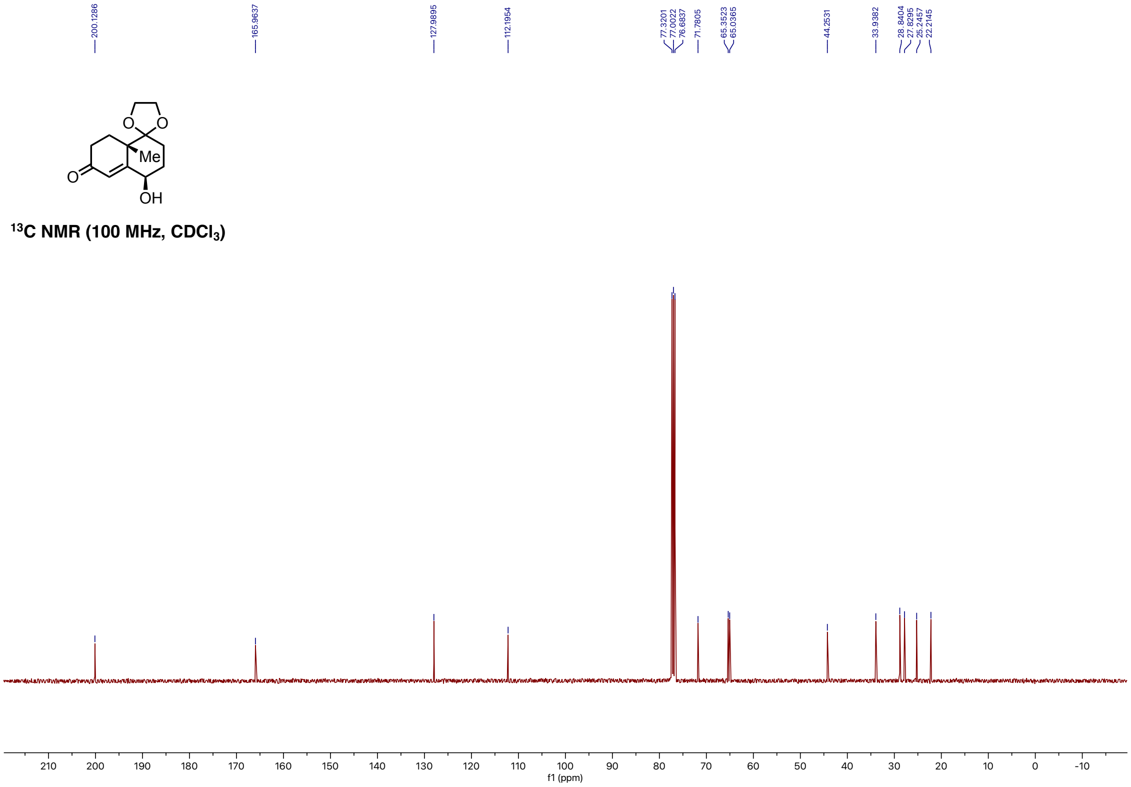

^{13}C NMR (100 MHz, $CDCl_3$)

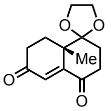

^{1}H NMR (400 MHz, $CDCl_3$)

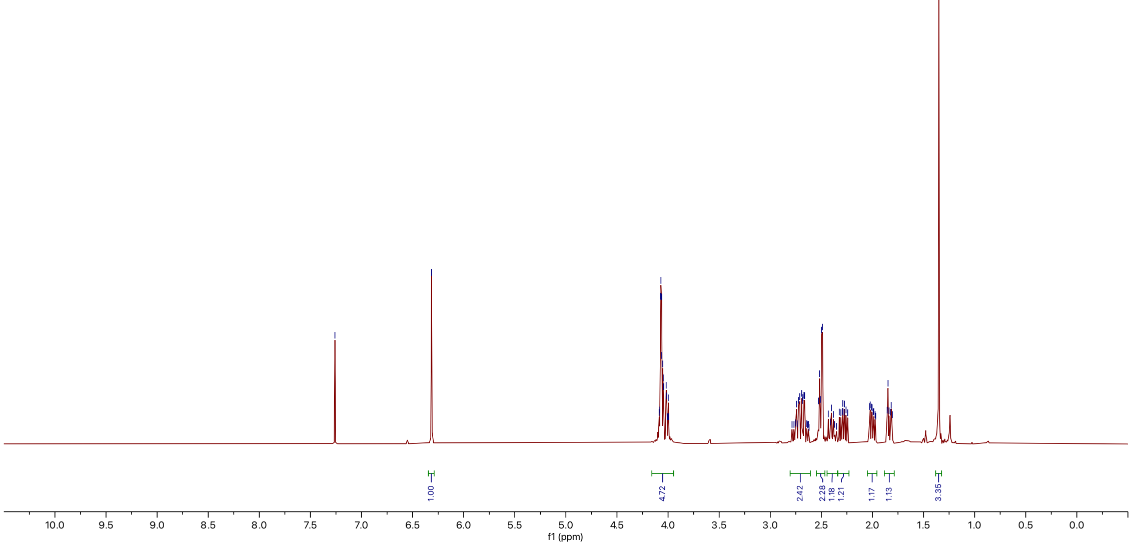

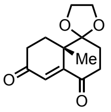

^{13}C NMR (100 MHz, $CDCl_3$)

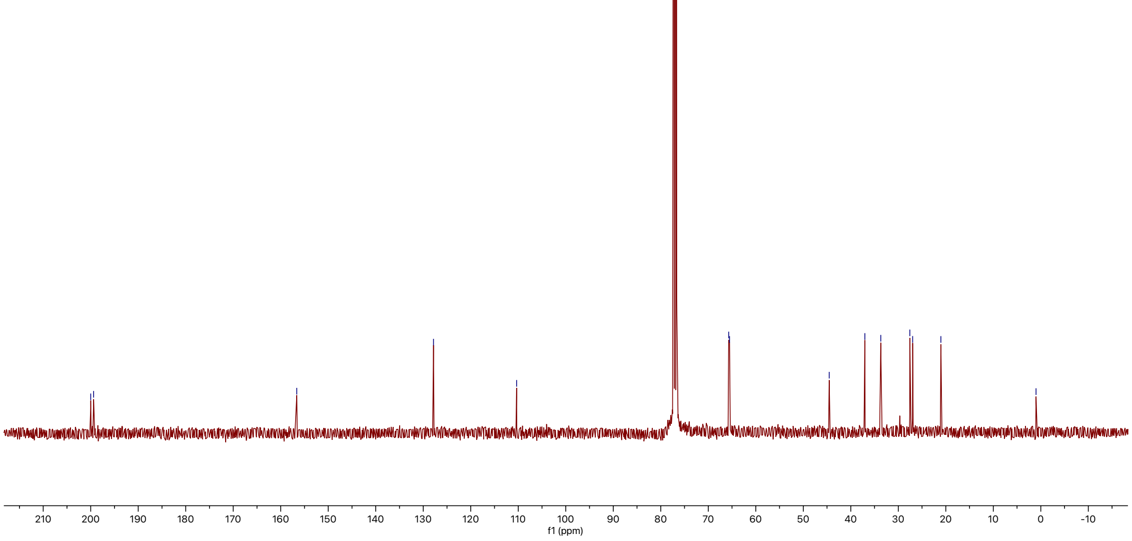

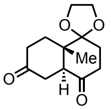

^{1}H NMR (400 MHz, $CDCl_3$)

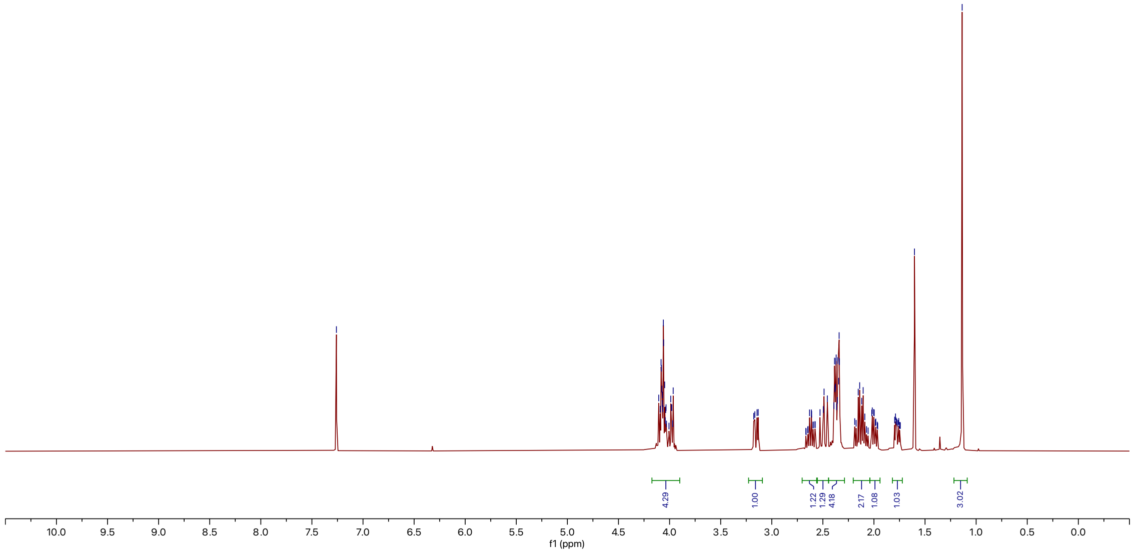

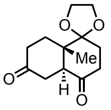

^{13}C NMR (100 MHz, $CDCl_3$)

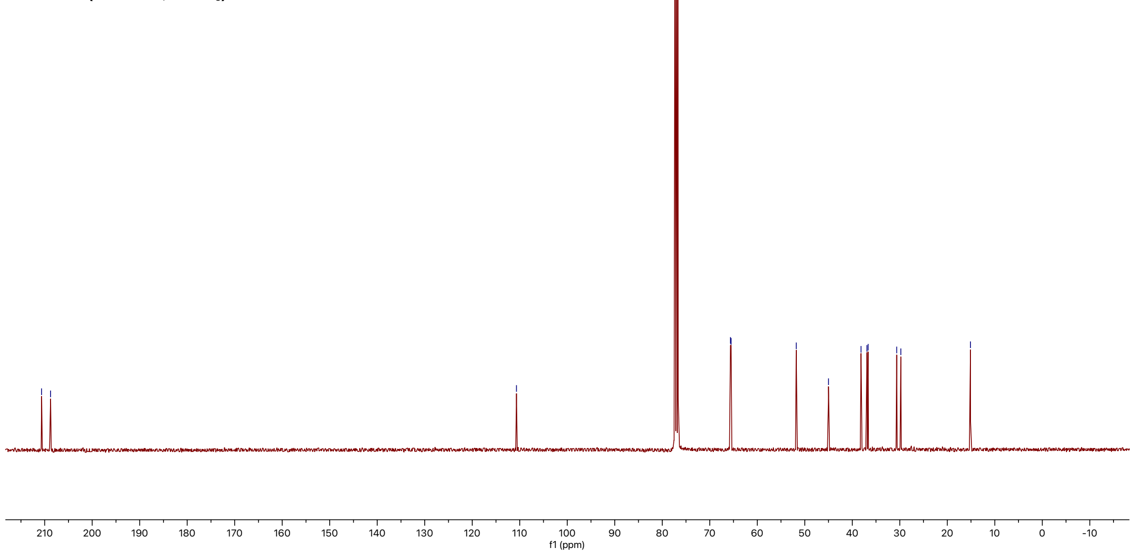

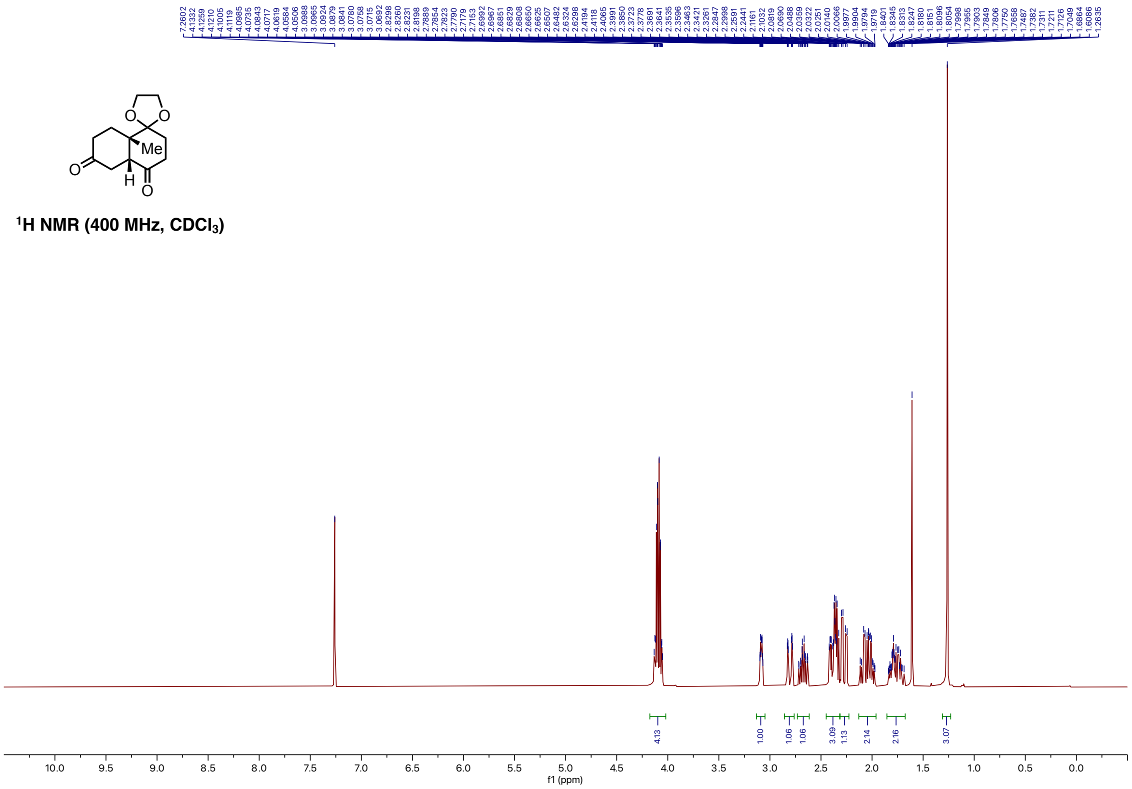

^{1}H NMR (400 MHz, $CDCl_3$)

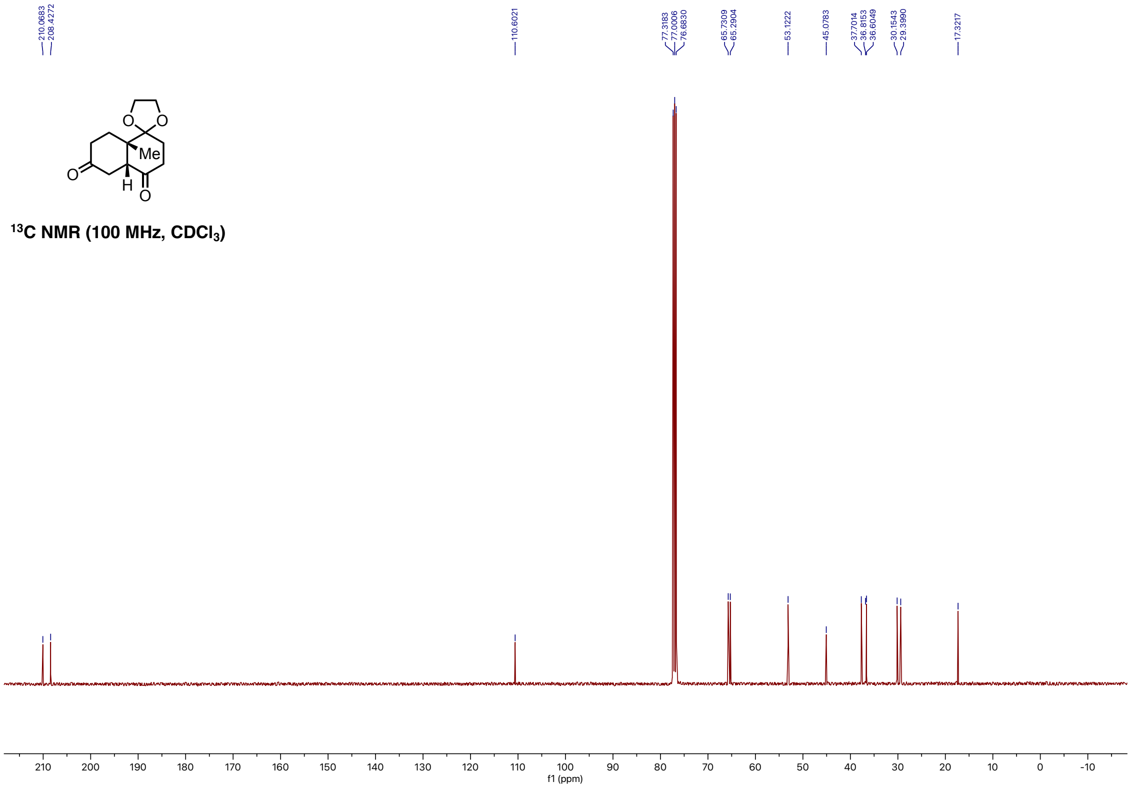

^{13}C NMR (100 MHz, $CDCl_3$)

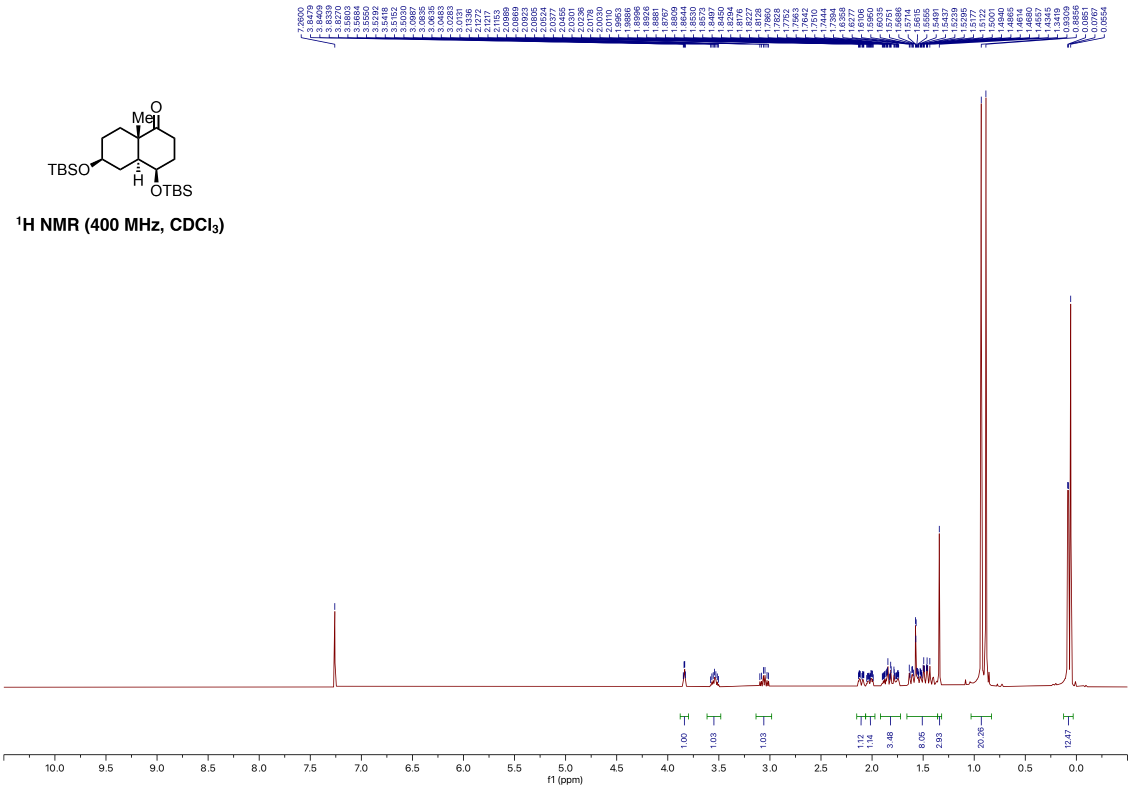

¹H NMR (400 MHz, $CDCl_3$)

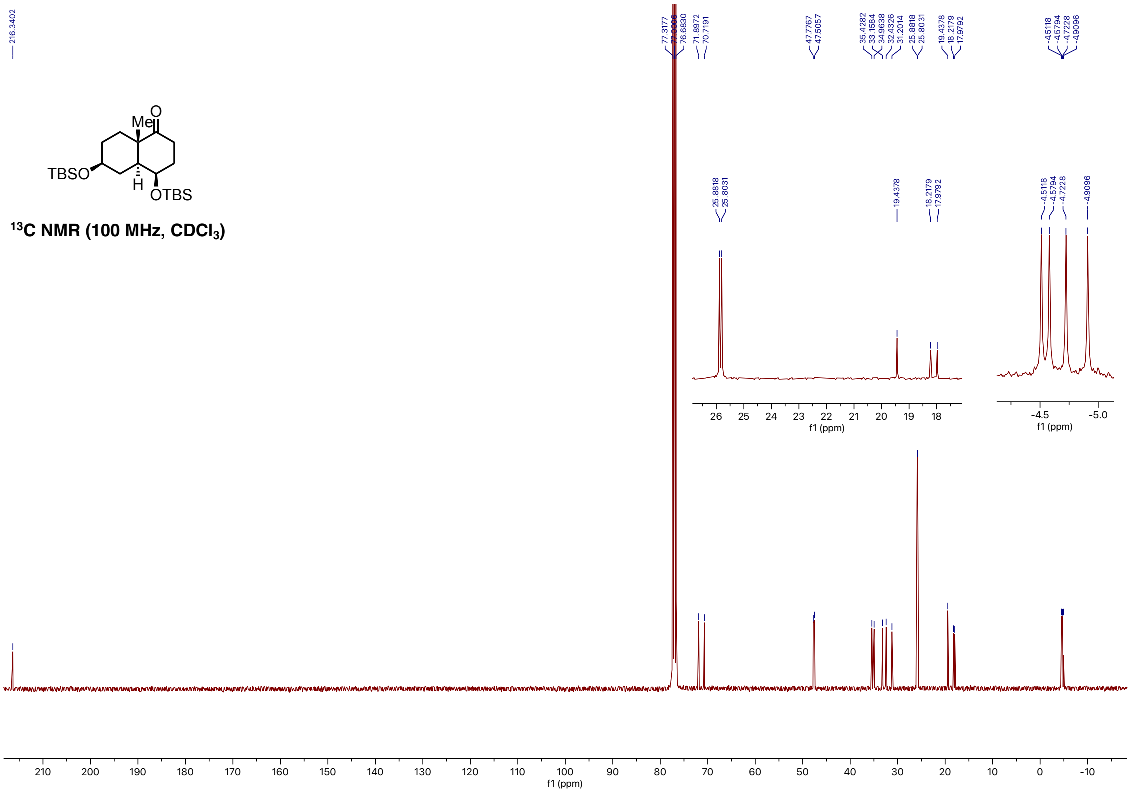

^{13}C NMR (100 MHz, $CDCl_3$)

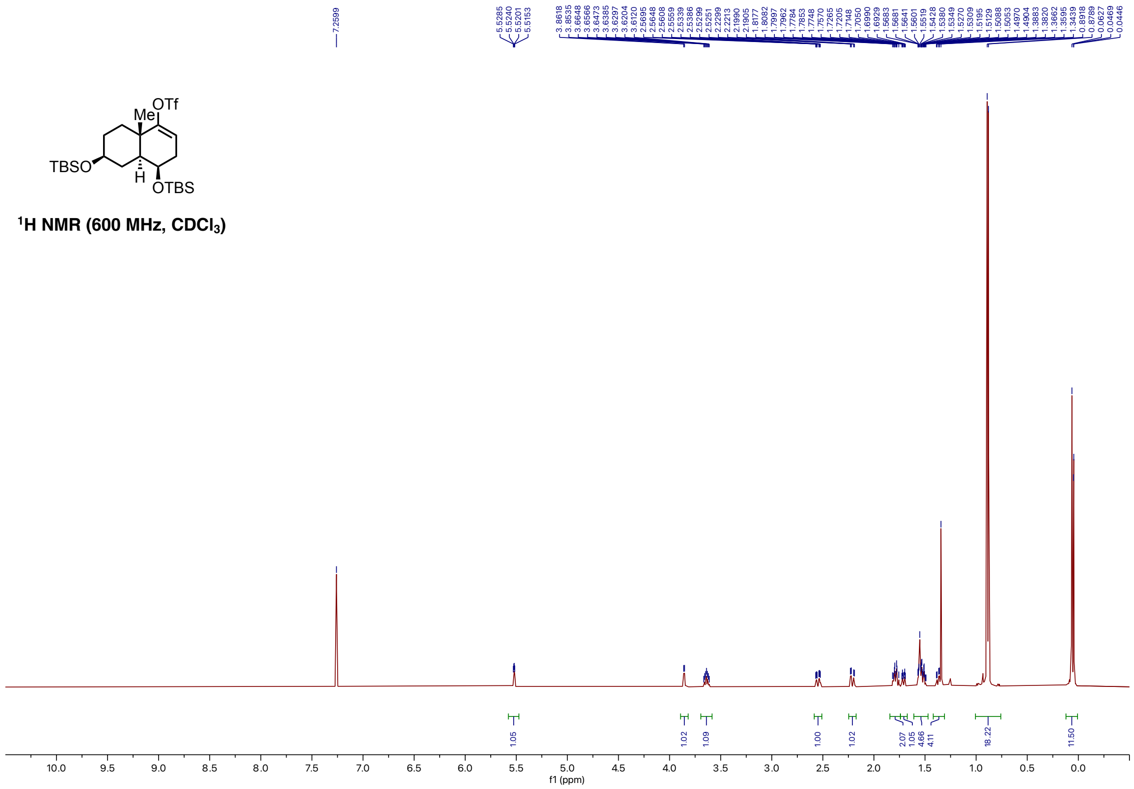

^{1}H NMR (600 MHz, $CDCl_3$)

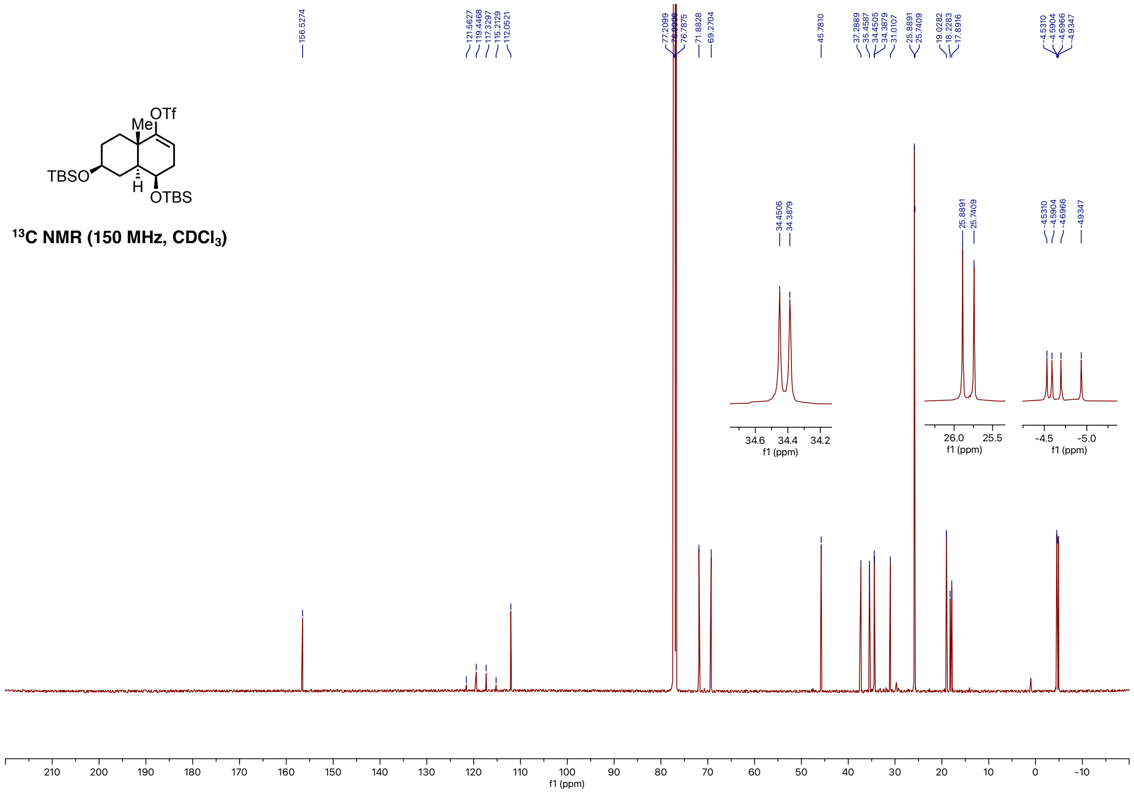

^{13}C NMR (150 MHz, $CDCl_3$)

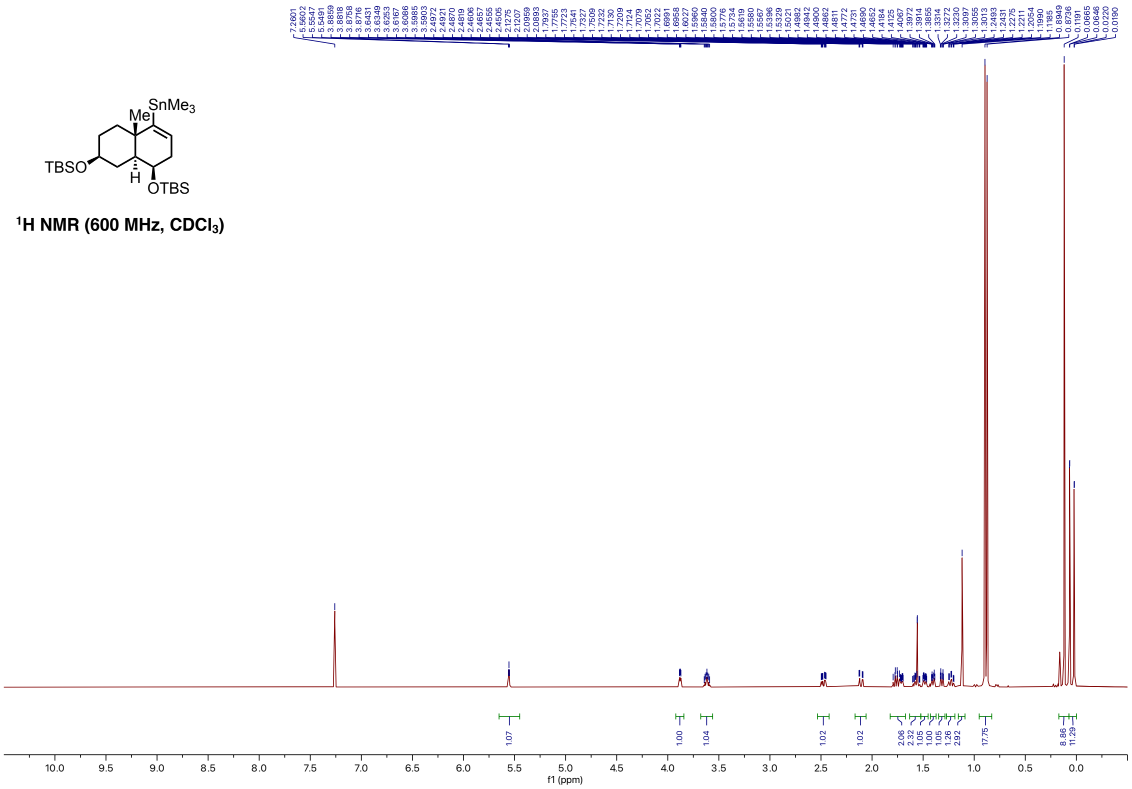

^{1}H NMR (600 MHz, $CDCl_3$)

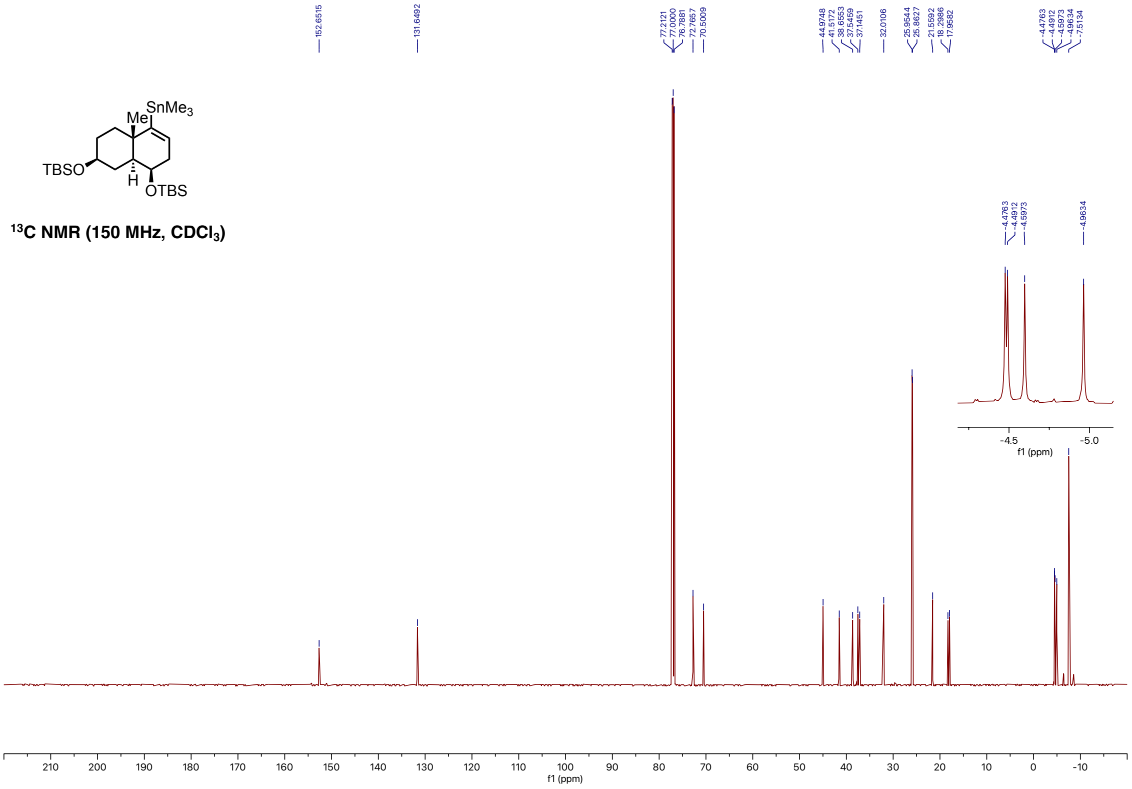

^{13}C NMR (150 MHz, $CDCl_3$)

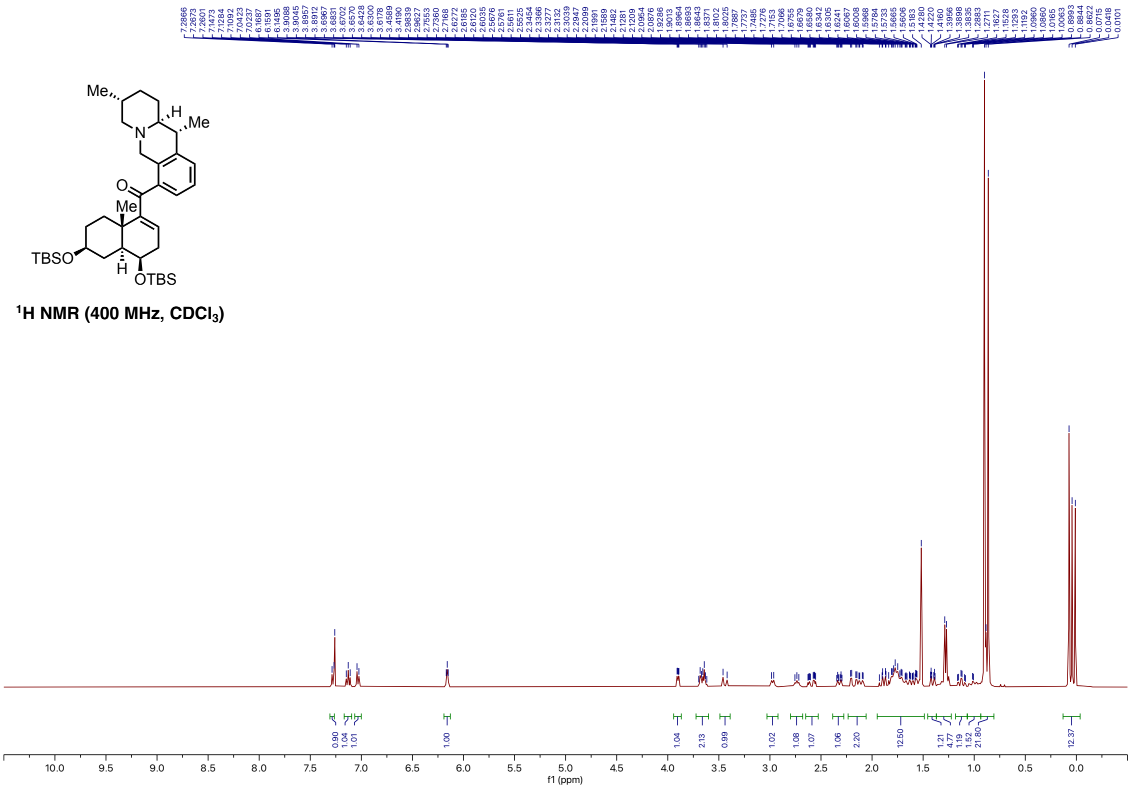

^{1}H NMR (400 MHz, CDCl$_3$)

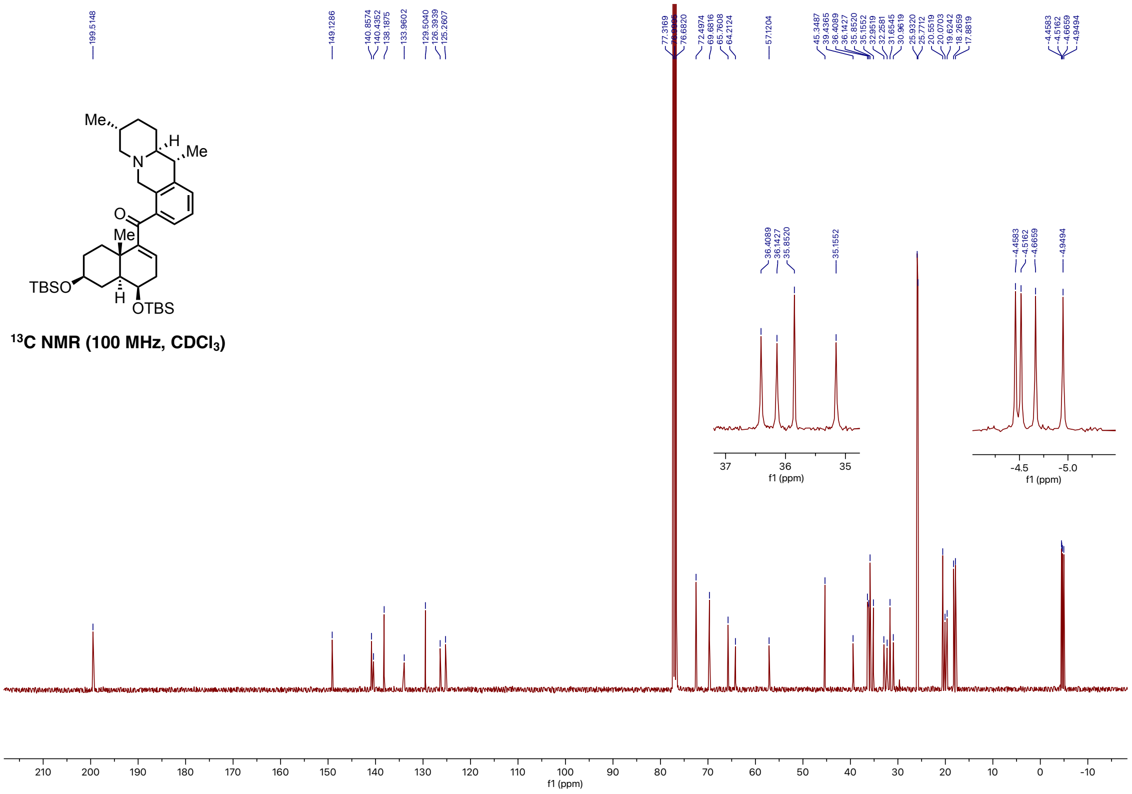

^{13}C NMR (100 MHz, CDCl$_3$)

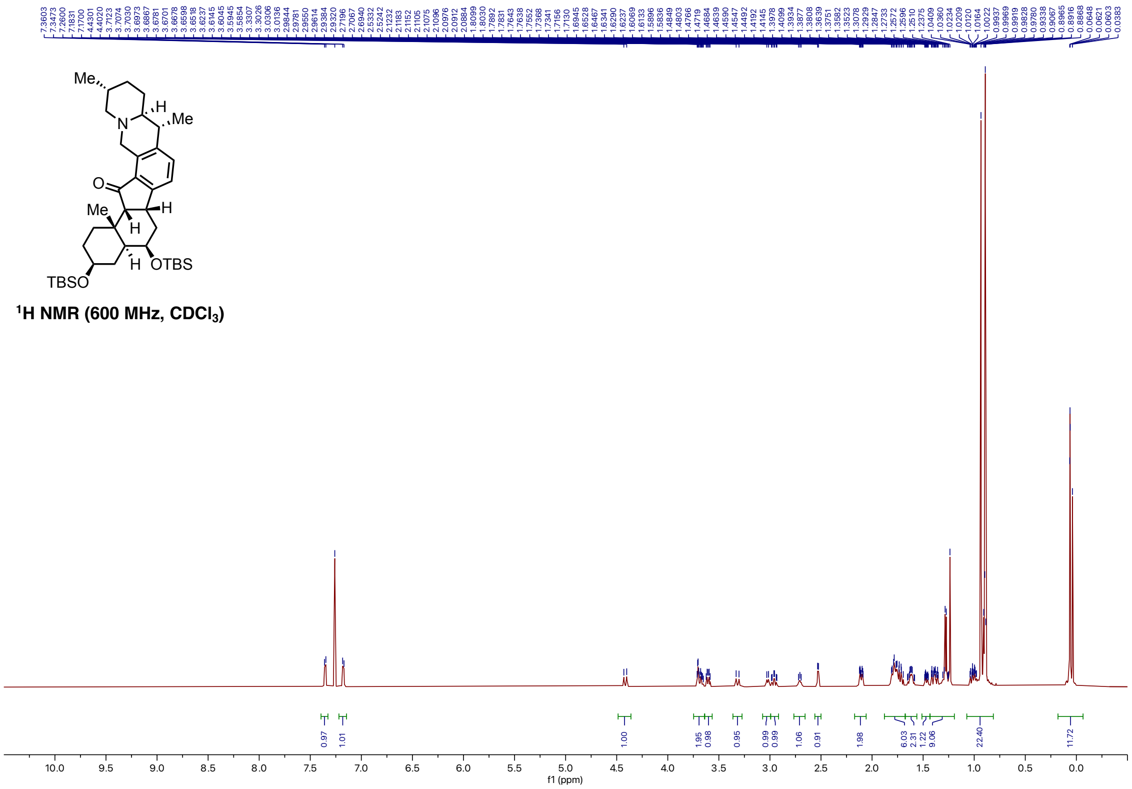

^{1}H NMR (600 MHz, $CDCl_3$)

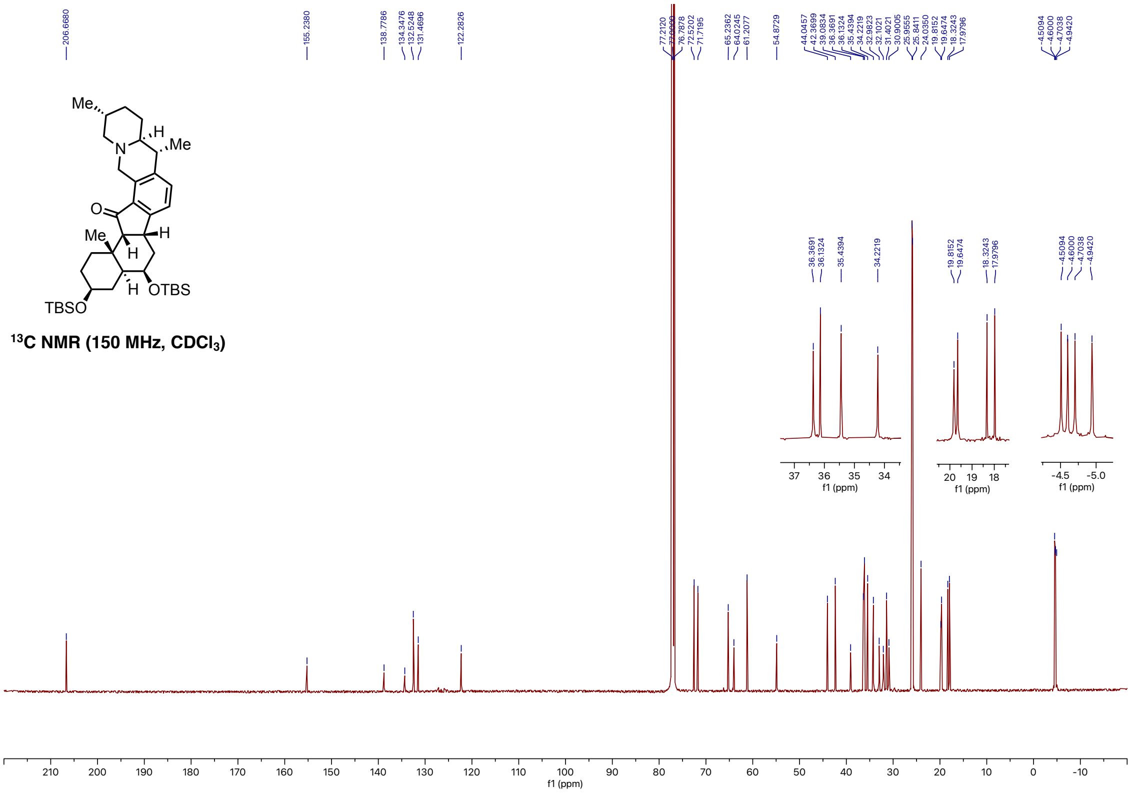

^{13}C NMR (150 MHz, $CDCl_3$)

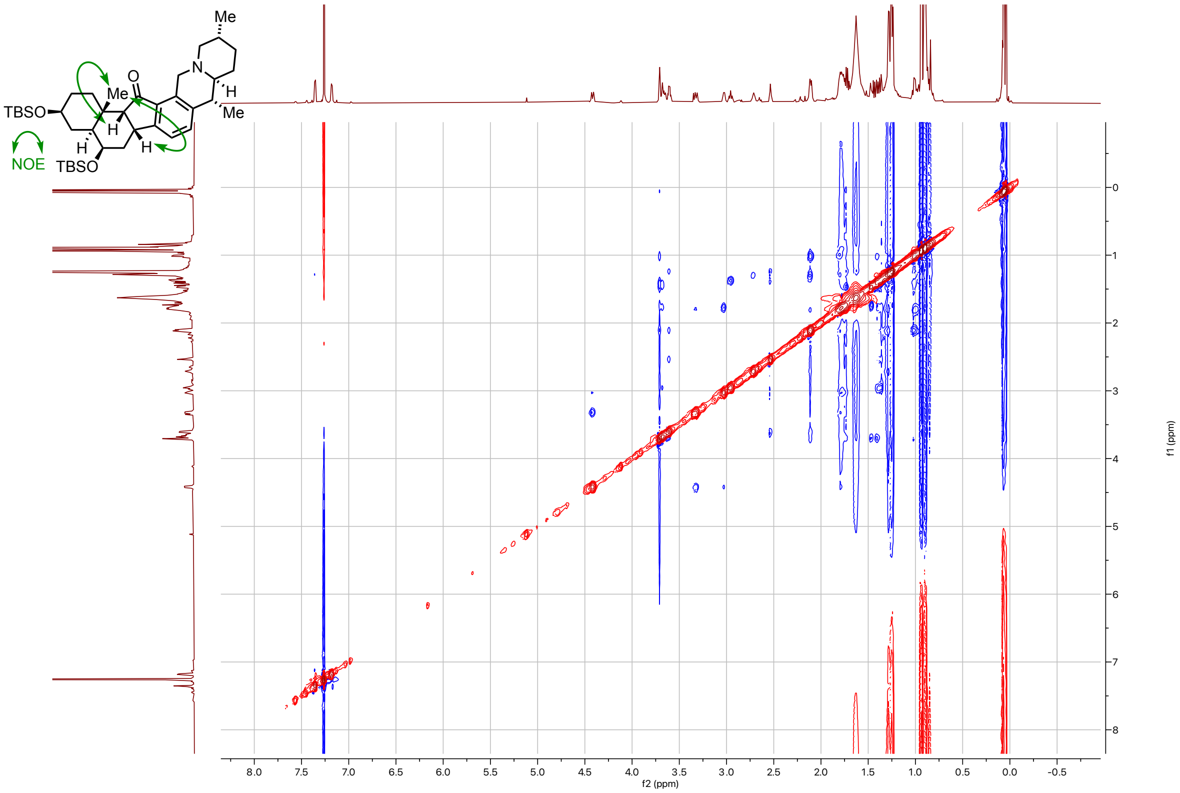
Me
O
N
Me
H
TBSO
H
H
Me
H
TBSO
NOE
f2 (ppm)
f1 (ppm)

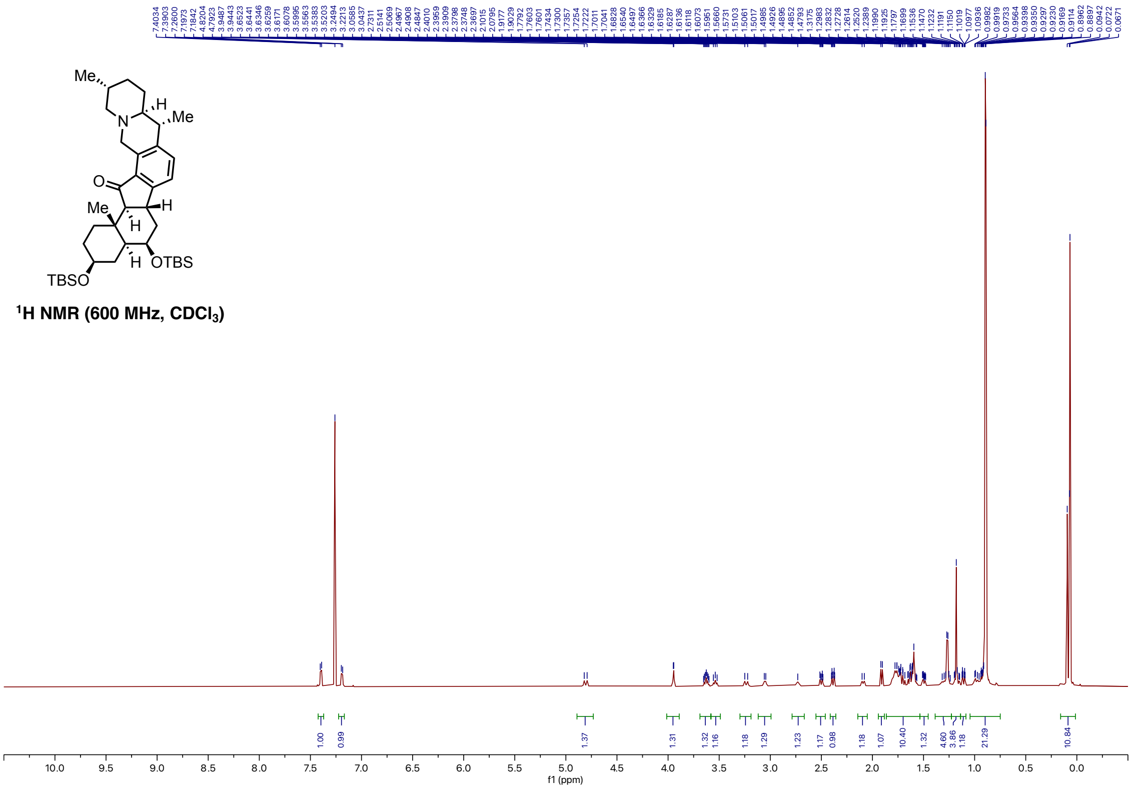

^{1}H NMR (600 MHz, CDCl$_3$)

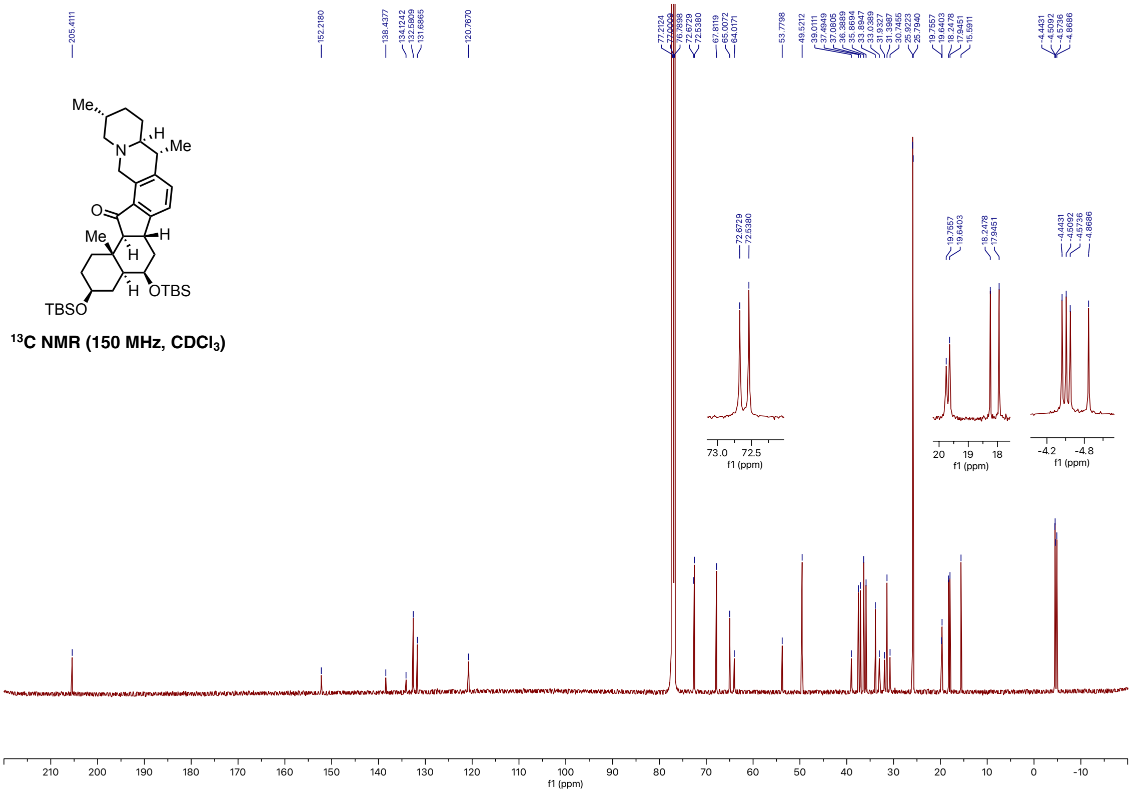

^{13}C NMR (150 MHz, CDCl$_3$)

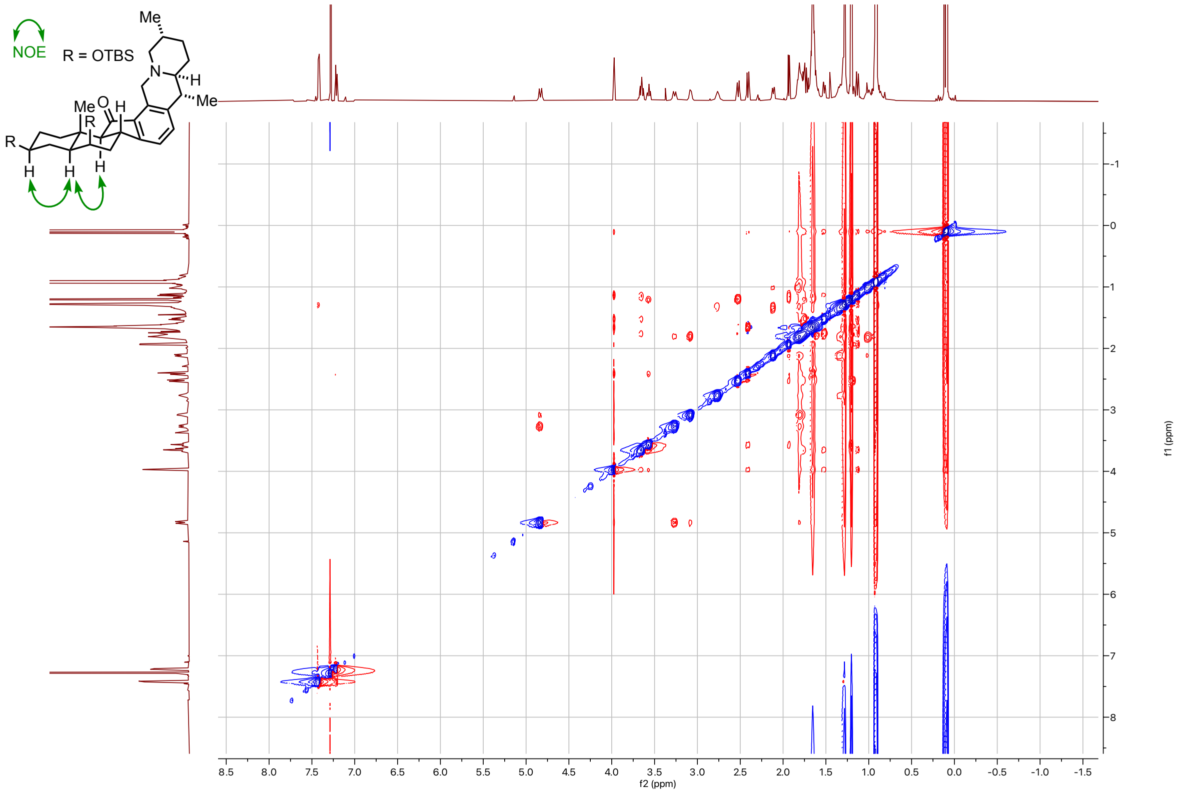
NOE
R = OTBS
Me
N
H
Me
Me
O
H
R
R
H
H
H
f2 (ppm)
f1 (ppm)

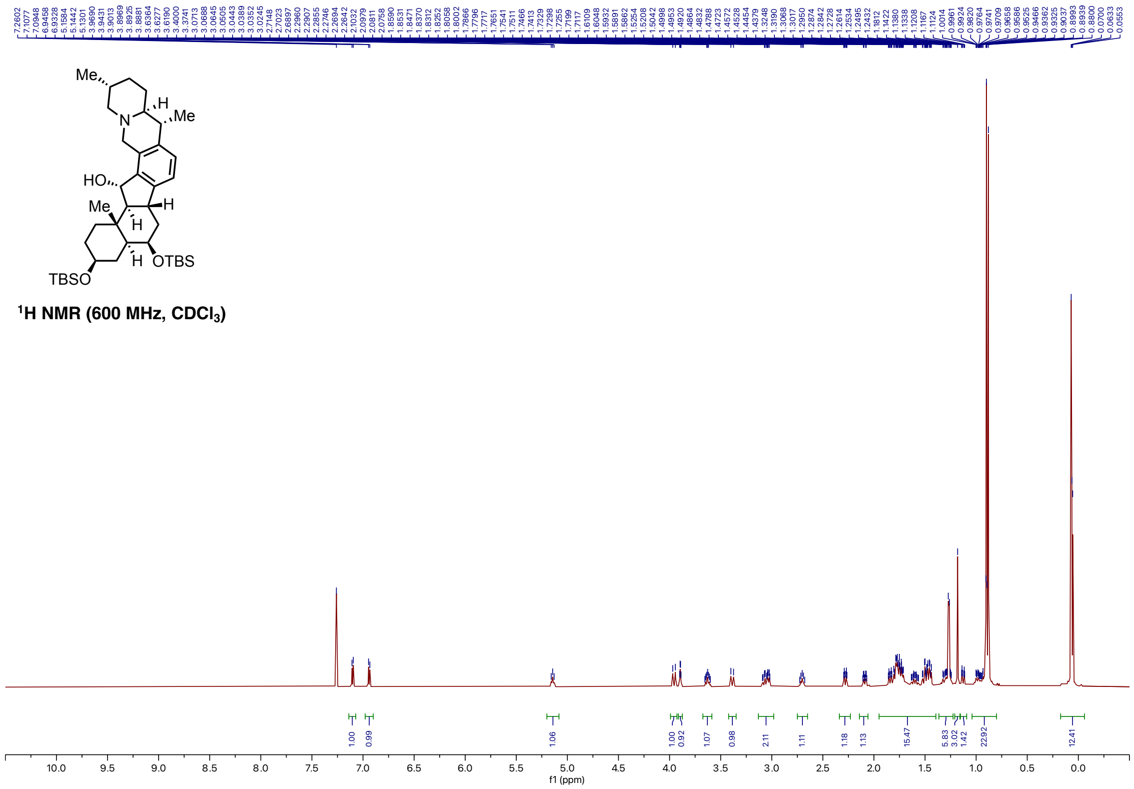

^{1}H NMR (600 MHz, $CDCl_3$)

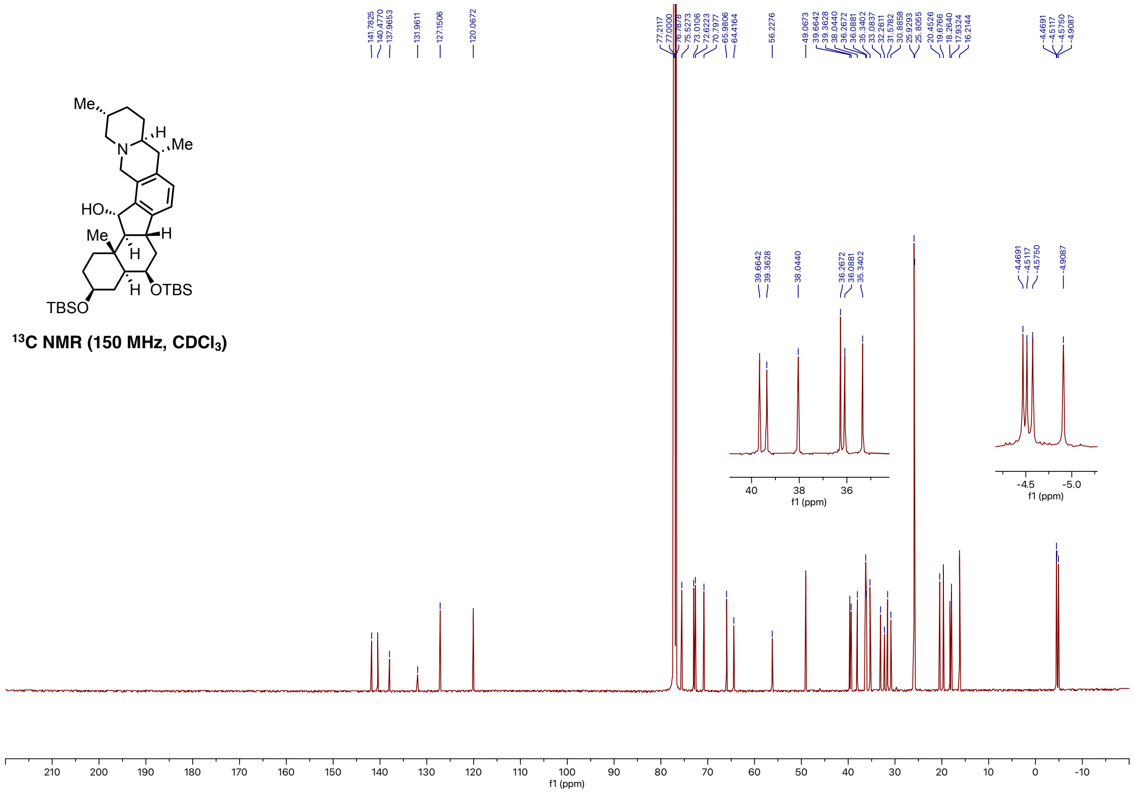

^{13}C NMR (150 MHz, $CDCl_3$)

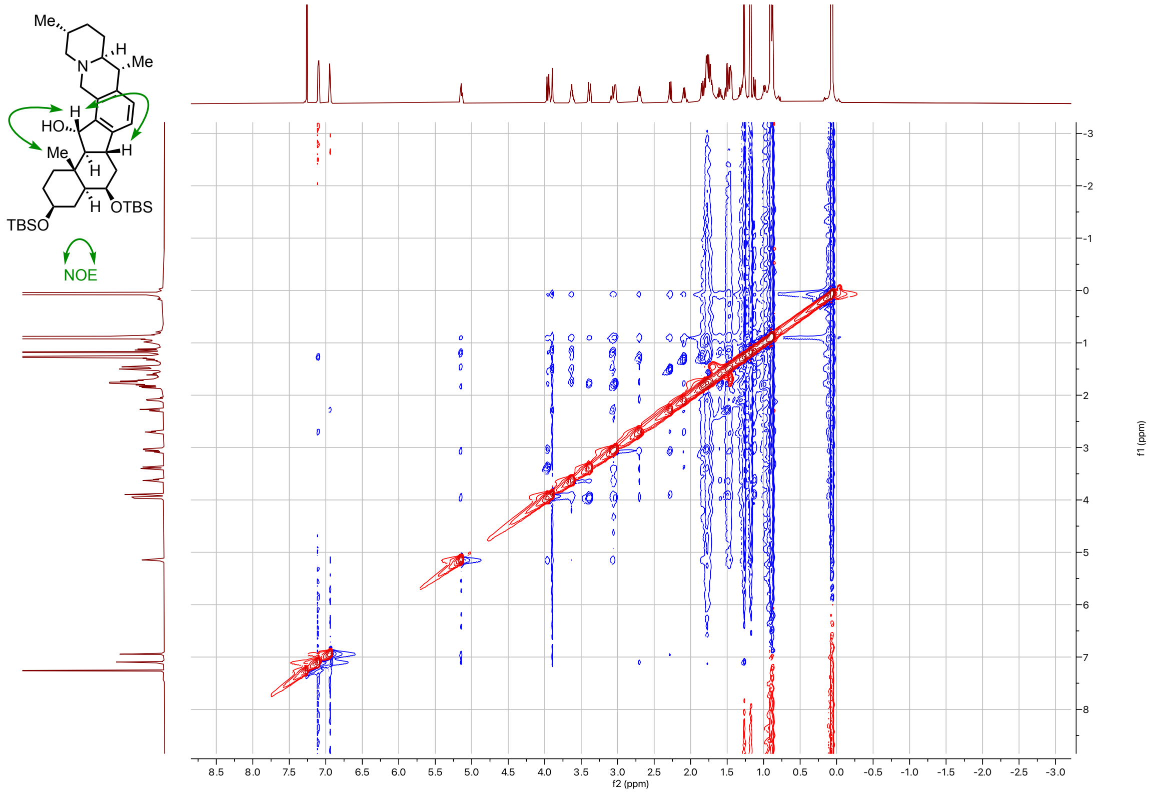
Me
H
Me
HO
H
Me
H
H
OTBS
TBSO
NOE
f2 (ppm)
f1 (ppm)

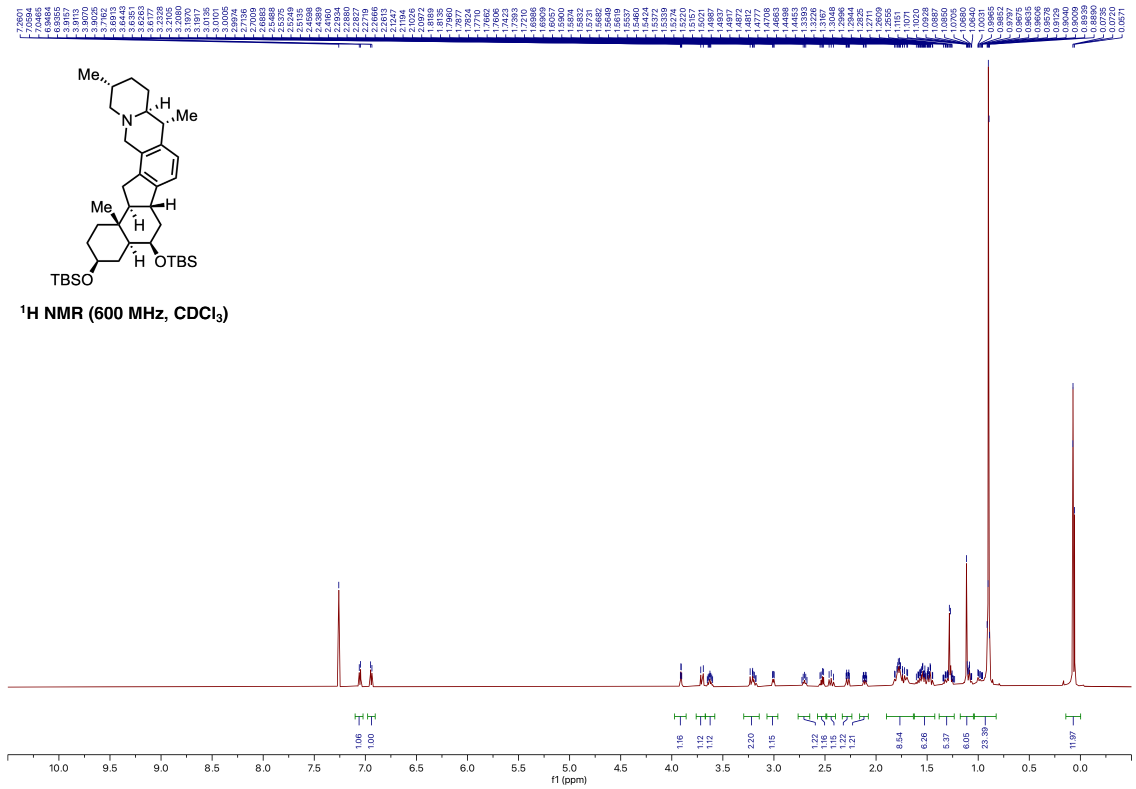

^{1}H NMR (600 MHz, $CDCl_3$)

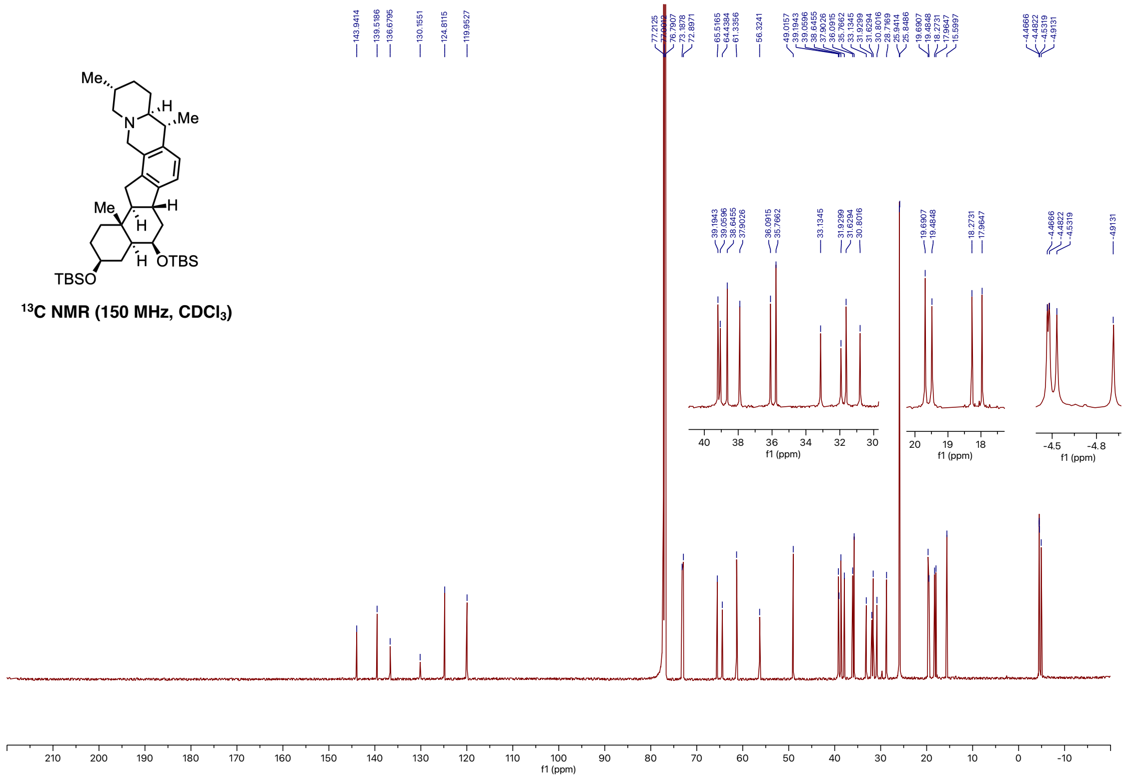

^{13}C NMR (150 MHz, $CDCl_3$)

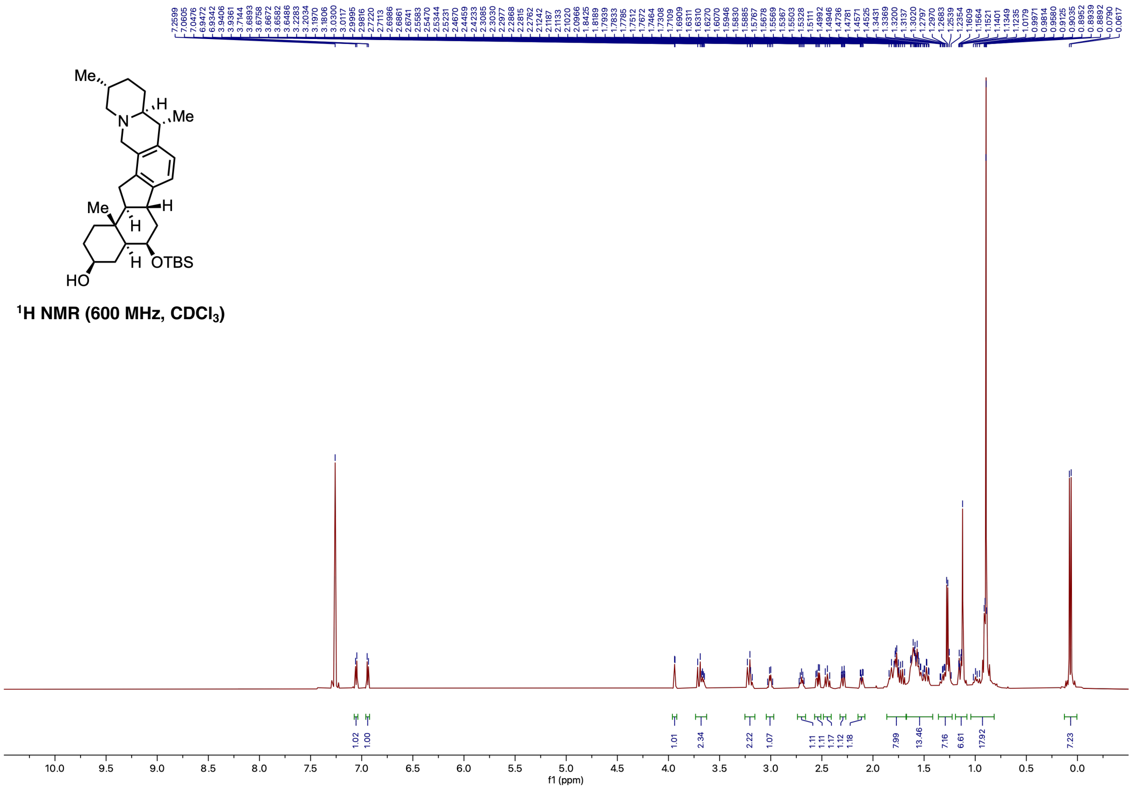
Me
H
Me
N
Me
H
H
H
OTBS
HO
1H NMR (600 MHz, CDCl3)
f1 (ppm)

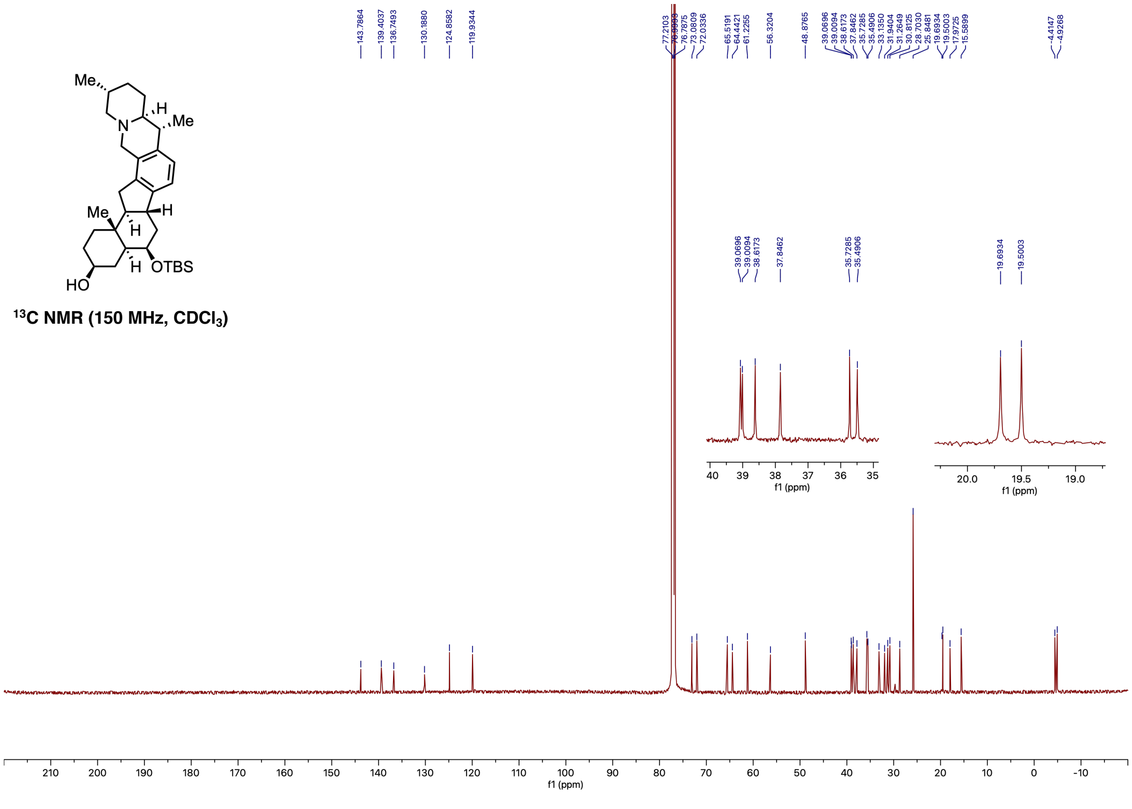
Me
H
Me
N
Me
H
H
H
OTBS
HO
13C NMR (150 MHz, CDCl3)
f1 (ppm)

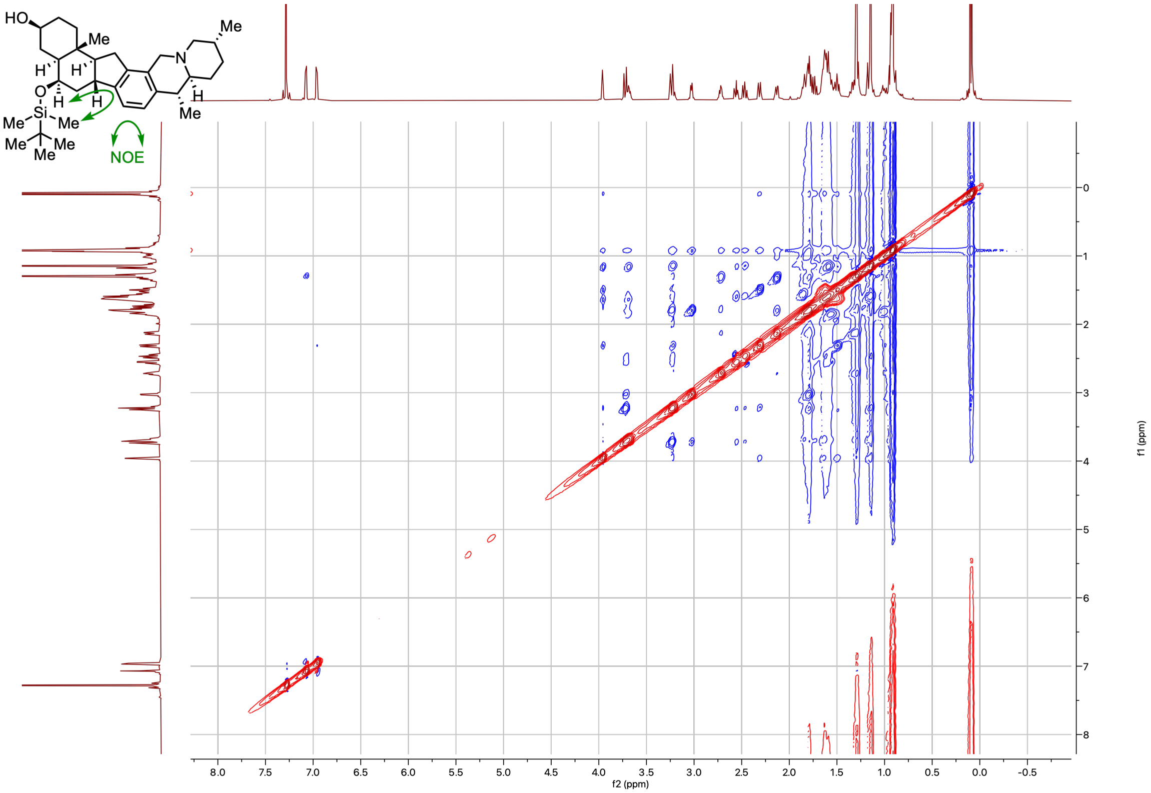
HO
Me
N
Me
H
H
O
H
H
Si
Me
Me
Me
Me
Me
Me
NOE
f2 (ppm)
f1 (ppm)

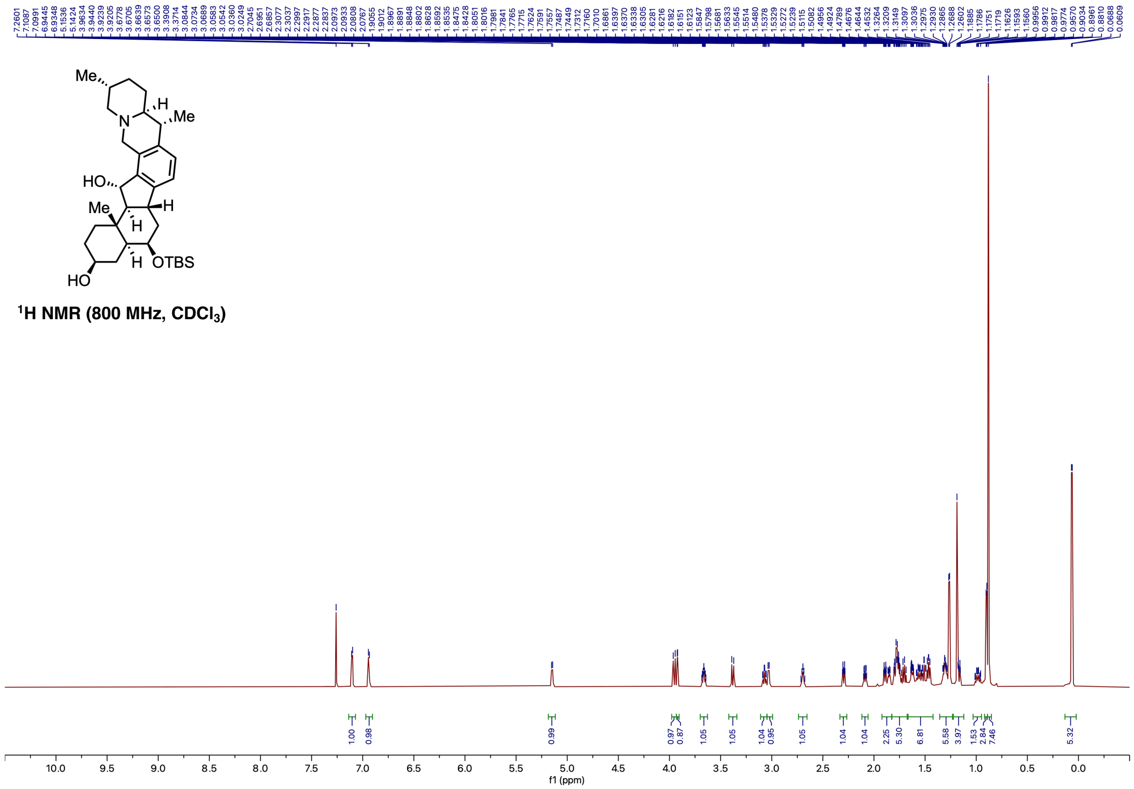

^{1}H NMR (800 MHz, $CDCl_3$)

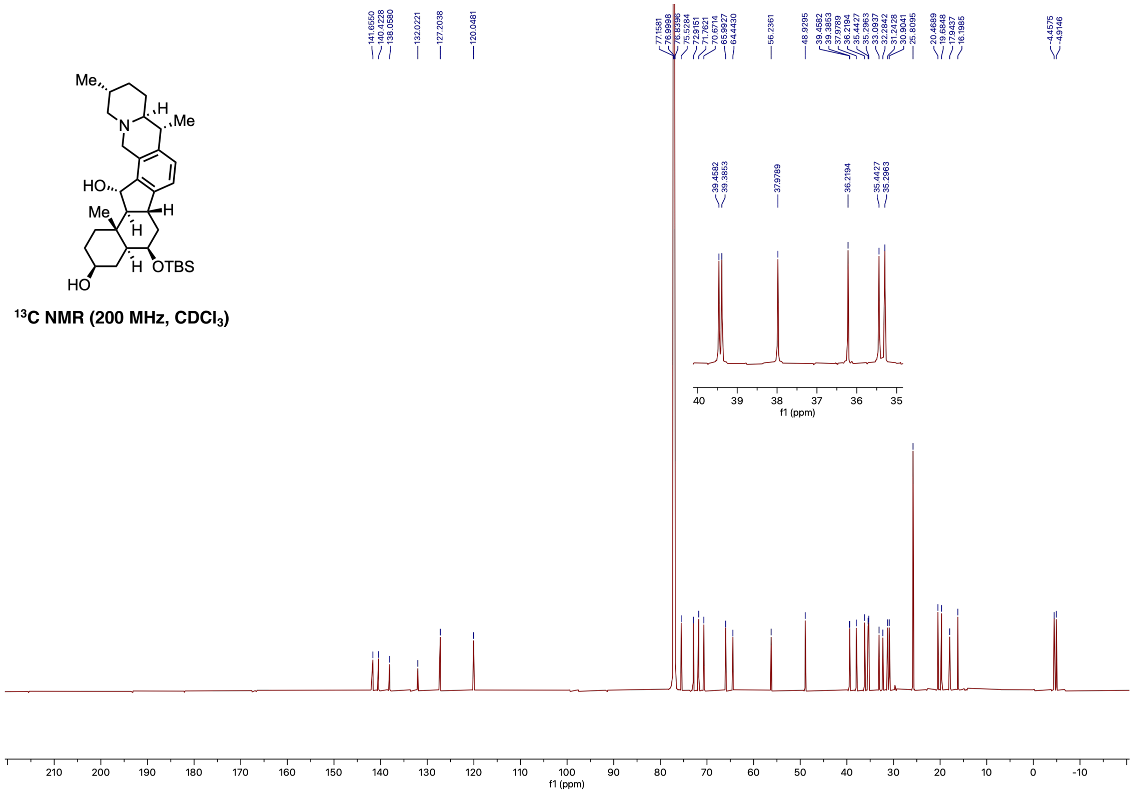

^{13}C NMR (200 MHz, $CDCl_3$)

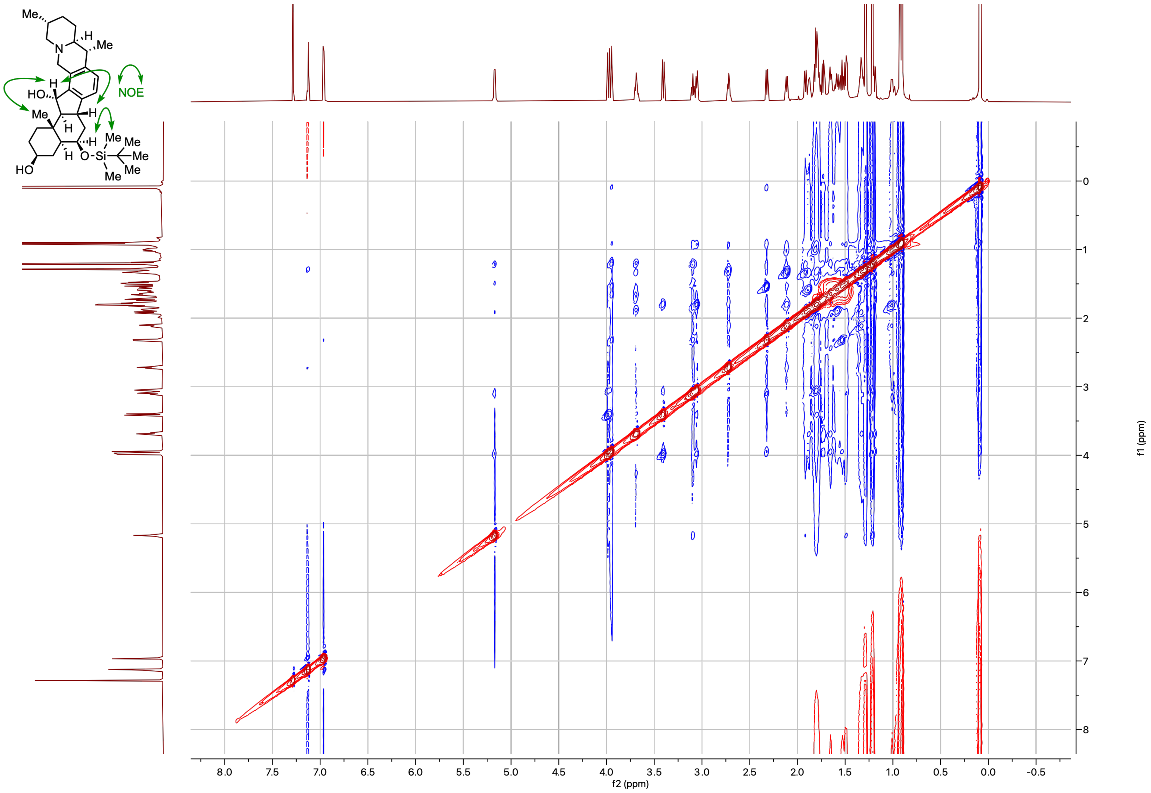

Me
H
Me
N
H
HO
NOE
Me
H
H
H
H
O
Si
Me
Me
Me
Me
Me
HO
H
f2 (ppm)
f1 (ppm)

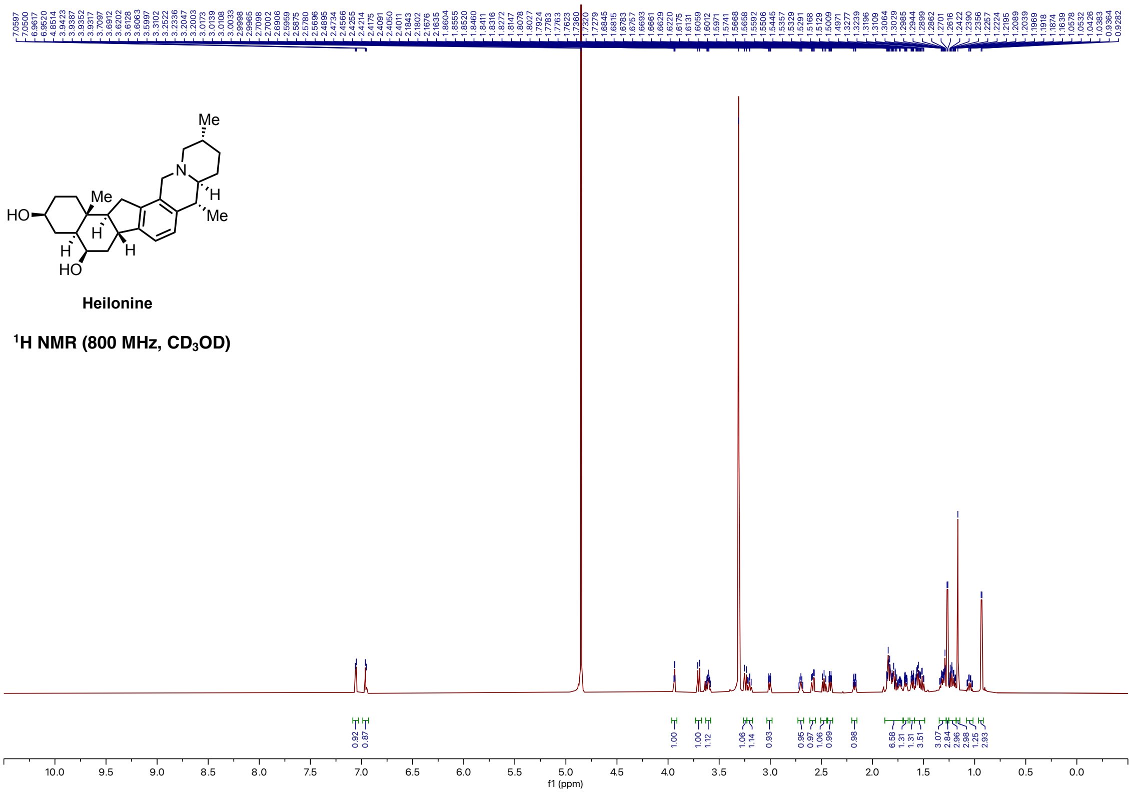

Heilonine

^{1}H NMR (800 MHz, CD_3OD)

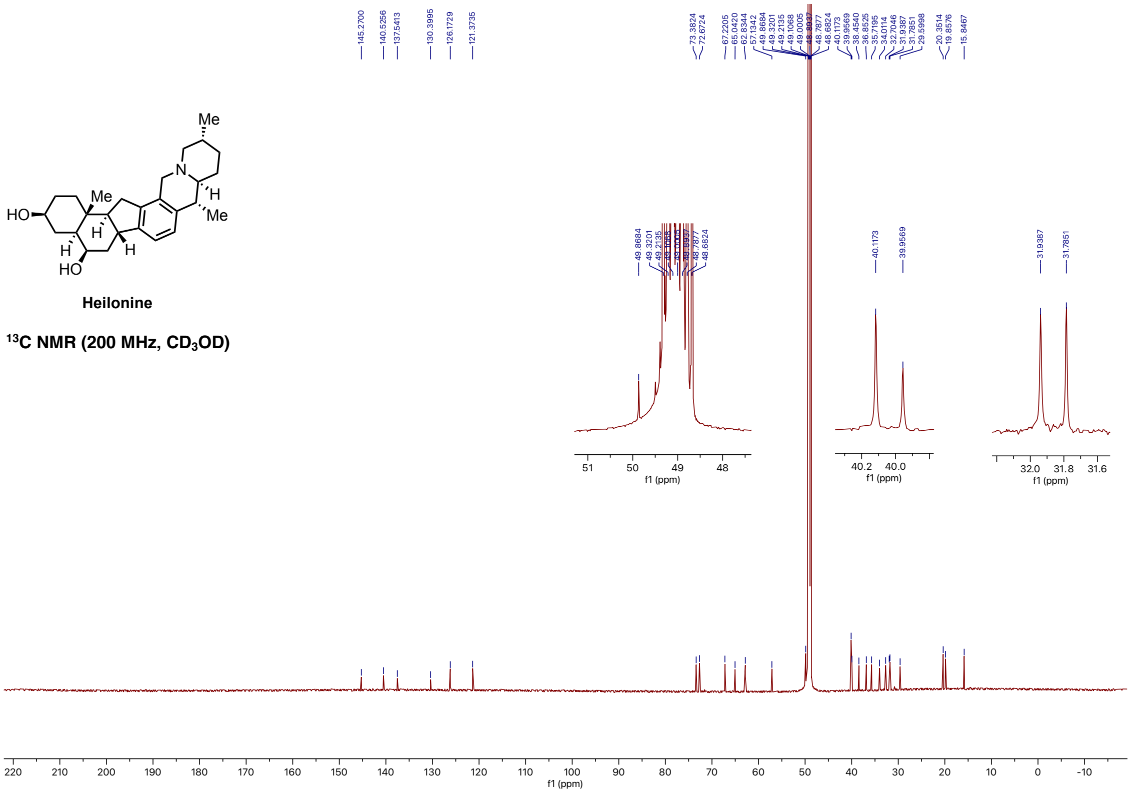

Heilonine

^{13}C NMR (200 MHz, CD_3OD)

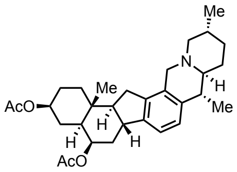

Heilonine diacetate

^{1}H NMR (600 MHz, CDCl$_3$)

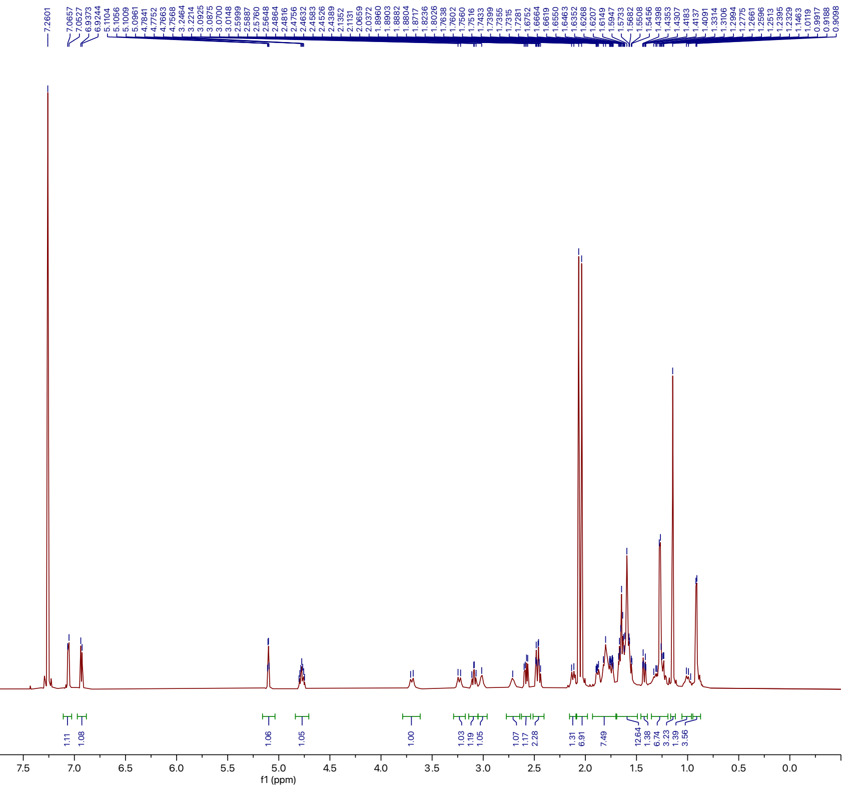

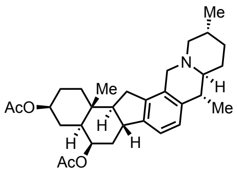

Heilonine diacetate

^{13}C NMR (150 MHz, CDCl$_3$)

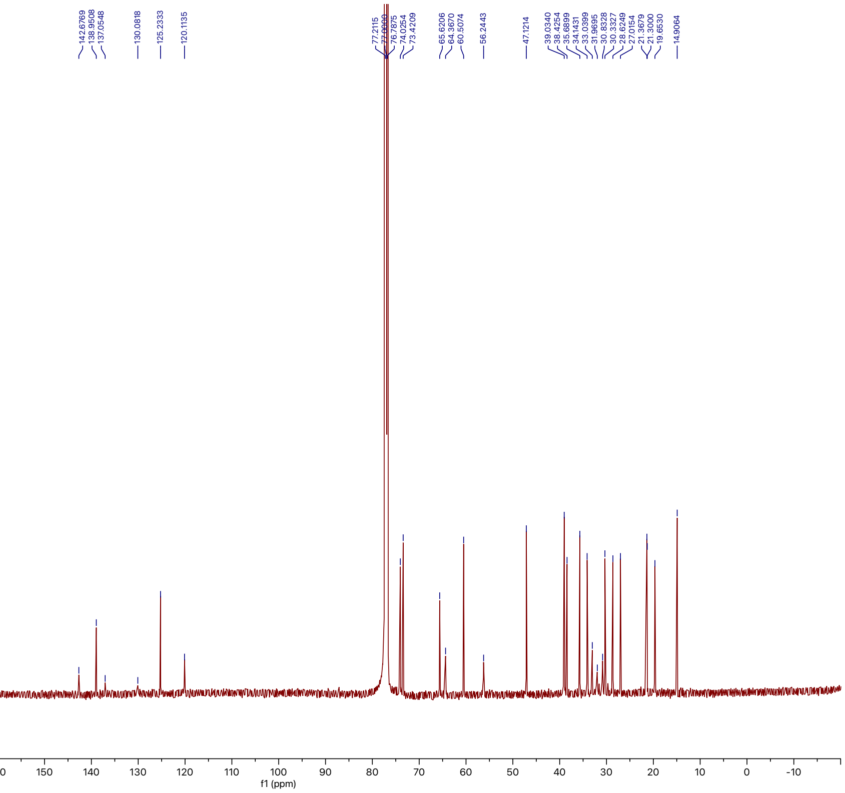

Supplement: Supplementary file 1 — ja3c13492_si_001.pdf [file ja3c13492_si_001.pdf]
